# Linking the transcriptome to physiology: response of the proteome of *Cupriavidus metallidurans* to changing metal availability

**DOI:** 10.1093/mtomcs/mfae058

**Published:** 2024-11-19

**Authors:** Diana Galea, Martin Herzberg, Dirk Dobritzsch, Matt Fuszard, Dietrich H Nies

**Affiliations:** Institute for Biology/Microbiology, Martin-Luther-University Halle-Wittenberg, 06099 Halle (Saale), Germany; Institute for Biology/Microbiology, Martin-Luther-University Halle-Wittenberg, 06099 Halle (Saale), Germany; Department of Analytical Chemistry, Helmholtz Centre for Environmental Research—UFZ, Leipzig 04318, Germany; Core Facility—Proteomic Mass Spectrometry, Charles Tanford Center, Martin-Luther-University Halle-Wittenberg, 06099 Halle (Saale), Germany; Core Facility—Proteomic Mass Spectrometry, Charles Tanford Center, Martin-Luther-University Halle-Wittenberg, 06099 Halle (Saale), Germany; Institute for Biology/Microbiology, Martin-Luther-University Halle-Wittenberg, 06099 Halle (Saale), Germany

**Keywords:** transenvelope efflux systems, zinc, metal homeostasis, *Cupriavidus metallidurans*, metal starvation, proteomics

## Abstract

*Cupriavidus metallidurans* CH34 is a metal-resistant bacterium. Its metal homeostasis is based on a flow equilibrium of metal ion uptake and efflux reactions, which adapts to changing metal concentrations within an hour. At high metal concentrations, upregulation of the genes for metal efflux systems occurs within minutes. Here, we investigate the changes in the bacterial proteome accompanying these genetic and physiological events after 1.5 cell duplications, which took 3 h. To that end, *C. metallidurans* CH34 and its plasmid-free derivative, AE104, either were challenged with a toxic metal mix or were cultivated under metal-starvation conditions, followed by bottom-up proteomics. When metal-shocked or -starved cells were compared with their respective controls, 3540 proteins changed in abundance, with 76% appearing in one, but not the other, condition; the remaining 24% were up- or downregulated. Metal-shocked *C. metallidurans* strains had adjusted their proteomes to combat metal stress. The most prominent polypeptides were the products of the plasmid-encoded metal-resistance determinants in strain CH34, particularly the CzcCBA transenvelope efflux system. Moreover, the influence of antisense transcripts on the proteome was also revealed. In one specific example, the impact of an asRNA on the abundance of gene products could be demonstrated and this yielded new insights into the function of the transmembrane efflux complex ZniCBA under conditions of metal starvation.

## Graphical Abstract

**Graphical Abstract:**
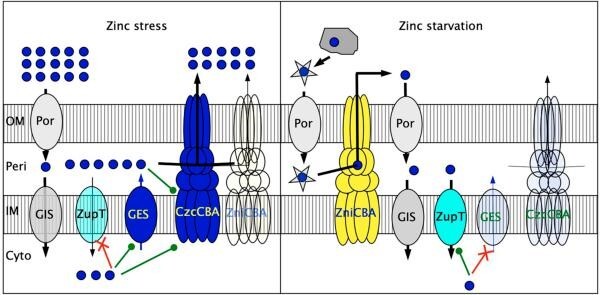
The transenvelope pump ZniCBA was upregulated under metal-starvation conditions and an antisense RNA was involved in regulation of this process. This suggests a possibly novel function of these efflux systems.

## Introduction

*Cupriavidus metallidurans* CH34 is a master in metal ion homeostasis [1–3]. In mesophilic environments, this β-proteobacterium is able to adjust its zinc ion homeostasis to external zinc concentrations from the lower nM to the mM range, as well as being capable of handling low or high concentrations of other divalent metal cations. Determinants on its chromosome are responsible for conferring this resistance and include a chromid and the two large plasmids pMOL28 and pMOL30 [4–8].

Resistance to the transition metal cations of Co(II), Zn(II), Ni(II), and Cd(II) is based on efflux by members of the P_IB_-ATPases [9, 10], CDF proteins [11], or other protein families, and takes place from the cytoplasm to the periplasm [2]. From the periplasm, large transenvelope efflux systems, such as CzcCBA or CnrCBA, export these ions out of the cell [2, 12]. While the central CzcA or related proteins are able to transport cations across a proteolipid membrane *in vitro* [13–16], biochemical and genetic studies making use of multiple deletion mutants clearly indicate that the *in vivo* function of these efflux complexes is export from the periplasm to the outside of the cell [17–24].

While at high metal concentrations redox changes plus metal efflux are the predominant homeostatic processes, a flow equilibrium of import and export processes governs metal homeostasis at more ambient metal concentrations (e.g. zinc concentrations between 150 nM and 100 µM) [25, 26]. The flow equilibrium transforms the energy used for the simultaneously occurring transport processes of numerous cations into the appropriate composition of the cytoplasmic, and presumably also the periplasmic, metal cation pools, which subsequently determine the competition between these cations for the metal-binding sites of the proteins in these compartments [27, 28]. Pulse-chase experiments revealed that *C. metallidurans* cells needed 15 min to 1 h at 30°C to adapt its physiology to changing metal availability [25].

Uptake of Zn(II) and related cations, such as Co(II) and Cd(II), is accomplished by at least 10 import systems with broad substrate specificity [29–31]. In contrast, metal ion efflux or removal by other mechanisms is a metal-specific process, which is not based on the substrate specificity of the efflux system but on the regulation of the expression of the respective gene(s). Efflux of zinc ions is mediated by the P_IB2_-type ATPase, ZntA [18, 32]. While ZntA and the related proteins CadA and PbrA export zinc and cadmium ions with similar substrate specificities [18], the respective MerT-type regulators ZntR, CadR, and PbrR are metal selective [33–40]. Similarly, metal specificity of the regulation of expression of nickel resistance is based on the discrimination between nickel and cobalt and zinc ions by the nickel sensor, CnrX [41–45].

Consequently, Zn(II) is exported by the inner membrane exporters ZntA, CdfX, CzcD, and CzcP and further from the periplasm to the outside by CzcCBA. The inner membrane efflux systems all have slightly different functions with respect to zinc export [46]. Expression of their cognate genes is controlled by ZntR or the two-component system CzcRS [18, 47], depending on the cytoplasmic or periplasmic metal ion concentration, respectively. Export of Co(II) is by DmeF to the periplasm and out of the cell by CzcCBA and CnrCBA, while export of Cd(II) is by ZntA, CadA, and CzcCBA, and that of Ni(II) by DmeF, CnrT, and CnrCBA. Copper resistance is a special case and results from an interplay of periplasmic oxidation of the more toxic Cu(I) to Cu(II), efflux of Cu(I) from the cytoplasm to the periplasm by P_IB1_-type ATPases, efflux from the cell by transenvelope protein complexes such as CusCBA, with a minor contribution by other factors [19]. Chromate resistance is also based on efflux [48–51], arsenate resistance on reduction to arsenite followed by efflux [52–54], and mercury resistance on uptake of Hg(II) and reduction to the volatile metallic Hg(0) [55]. Since the environments of *C. metallidurans* contain not one but metal mixtures with different contents of individual metals [56], this allows this bacterium to export metal cations via parallel export routes with specific rates adjusted to the content of this individual metal, which is sensed by cytoplasmic, two-component or other regulatory systems.

As anticipated, expression of the various metal-resistance determinants of *C. metallidurans* (e.g*. czc, cnr, cop1, cop2, chr, ars*, and *mer*) was upregulated when exponentially growing cells were challenged for 10 min with a toxic mixture of metal ions [7, 8, 57, 58]. Moreover, in many cases, also antisense transcripts of these determinants were changed in expression. While the physiological adaptation of the cells to changing metal concentration occurs in the time range of 15–40 min [25], gene expression peaks at 2–30 min and returns to the initial expression level after 1 h [59, 60].

This current study investigates the outcome of the physiological and transcriptomic changes of *C. metallidurans* during its adaptation to changing metal availability. We analyze the proteome of this bacterium after these processes have occurred and demonstrate that altered abundances of sense and antisense RNAs also result in changes to the proteome. One example demonstrates how an antisense RNA is involved in reassignment of the function of a transenvelope efflux complex. Consequently, this proteome study completes on a different timescale the previous investigation of metal homeostasis of *C. metallidurans* at the onset of the adaptation process by transcriptomics and during this process by pulse-chase experiments, thereby linking the transcriptome to physiology.

## Results

### Experimental strategy

Exponentially growing cells of *C. metallidurans* CH34 wild-type and its plasmid-free derivative AE104 were challenged with a strain-specific mixture of toxic metals, or EDTA (Ethylenediaminetetraacetic acid) as done for the transcriptome analysis [58]. These strain-specific mixtures contained the individual metals in the ratio of their toxicity for strains CH34 and AE104, respectively. The concentration of the mixture used was equal to the IC_50_ value of this mixture for the respective strain. Similarly, EDTA was also applied at the respective IC_50_ value for either strain. To give the cells time to produce the gene products and to possibly dilute out no longer useful gene products by growth, exponentially growing cells were incubated in the presence of their metal mix for 1.5 duplications, which took approximately 3 h in an aerobic incubation at 30°C.

All experiments were performed in triplicate, yielding six data sets after whole proteome analysis by tandem mass spectrometry (CH34_0, CH34 control; CH34_M, metal-shocked CH34; CH34_E, metal-starved CH34; and similarly AE104_0, AE104_M and AE104_E for strain AE104). The quantities of each protein in the supernatant and solubilized ultracentrifugation sediment were normalized to an overall number of 1.86 million proteins per cell as derived from the experimentally determined average protein content [61]. This gave a copy number per cell for the respective protein. For the six data sets, the mean values and deviations of the copy numbers were calculated and these values compared for CH34 cells with and without toxic metal treatment (CM0), and with EDTA (CME) treatment. The same was done for AE104 cells (AM0, AME) and a comparison between CH34 and AE104 under nonchallenging conditions was done. The full data set for the measurements under the six conditions and the five calculations is provided in the supplement, together with additional data, for instance the KEGG orthology category [62, 63] of the respective proteins. The overall results are provided as Supplementary Data Set.

Of the 6755 annotated open reading frames [1], 3502 proteins were identified at least once under one condition; however, 3253 proteins were never detected (Supplementary Data Set). From the 369 open reading frames, which were found on all four replicons with Rmet tags from Rmet_6403 and higher, annotated in a later annotation process, only 7 were found at least once as proteins. About half of the predicted proteins were identified, primarily those originally annotated. This matched the published result from a first determination of the proteome of nonchallenged cells of the plasmid-free strain AE104 [61] (Supplementary Fig. S1). The 10 proteins identified to have the highest copy numbers ranged between 18 000 and 66 500 copies per cell (Supplementary Table S1).

Furthermore, the protein abundances given in copy numbers between metal-shocked and -starved (EDTA-treated) CH34 or similarly treated AE104 cells compared to the control (Supplementary Table S2) yielded five distinct comparison categories, which were then evaluated (Table 1). Only values with Q ≥2 or Q ≤0.5 and D >1 were considered. This yielded an overall number of 3540 changes in which polypeptides were either increased or decreased in abundance (Table 1). In 2699 cases, the identified proteins were found only under one condition. In these cases, a ratio Q could not be calculated. In 841 instances (designated ‘quantitatively regulated’), proteins were found at least once under both conditions, which allowed calculation of a Q value and classification of the proteins into the groups ‘upregulated’ and ‘downregulated’, if the distance value D was >1. This indicated in the case of a single appearance of a protein under one condition that this value was outside the deviation span of the mean value of the result in the other condition. In the event of two single appearances, a D value could not be calculated because this would have been a division by zero. These comparisons were not further considered. Only in 327 instances (‘significantly regulated’), a protein could be measured at least twice under both conditions, which allowed the calculation of its up- or downregulation if D >1. Most of the measured changes of the proteome were simply appearances or disappearances of proteins under one condition. Only in 9.2% of the cases was a significant change in the copy number per cell determined (Table 1).

**Table 1.** Overview of the number of proteins up- or downregulated in the comparisons^a^

			Regulated		
Comparison	Total	(Dis)-appearing	Quantitatively	Significantly	Q_sense ≥2
CM0: up	490	346	144	53	18
CM0: down	470	315	155	76	0
CE0: up	193	79	114	61	3
CE0: down	393	320	73	27	1
0AC: up	387	339	48	14	nfc
0AC: down	330	263	67	19	nfc
AM0: up	244	190	54	25	9
AM0: down	472	375	97	29	4
AE0: up	327	296	31	10	2
AE0: down	234	176	58	13	0
Sum	3540	2699	841	327	37

^a^The total number of proteins upregulated/appearing or downregulated/disappearing is recorded in the second column. The first column lists the comparisons CM0 (CH34 metal-shocked to the control), CE0 (metal-starvation to the control), 0AC (AE104 to CH34 under nonchallenging conditions), AM0 (AE104 metal-shocked to the control), and AE0 (metal-starvation to the control). The proteins that disappeared or appeared and were not found in one of the conditions but in the other are listed in the third column. Quantitative results were defined as those with at least a single determination in each of the two conditions, with a two-fold ratio and a distance value >1. Significant results were those that came from at least two determinations in each of the two conditions, with a two-fold ratio and a distance value >1. The last column lists the significantly regulated proteins, which could be correlated with a significantly changed abundance of their sense RNA. The comparison between CH34 and AE104 control cells was not considered for correlation with the transcriptome data (nfc, not further considered).

As outlined in the Supplementary data, which details further constraints of the method used, small proteins of 100 amino acid residues or less in size (see Supplementary Fig. S2) and membrane-bound proteins without large hydrophilic domains (examined with the F_1_F_0_ ATPase subunits, Supplementary Table S3) were strongly underrepresented in this proteomic approach. The smallest number of proteins per cell that was measured at least twice under a particular condition was about 10 copies per cell. This indicated that for small proteins a copy number of about 50 should represent the lower detection limit, and for membrane-integral proteins without large hydrophilic extensions between 70 and 350 copies per cell should apply.

### Metal-challenged CH34 cells compared to the control and the products of metal-resistance determinants

In *C. metallidurans* CH34 wild-type cells treated with a toxic metal mixture, the abundance of 960 proteins changed, with 346 proteins appearing and 315 proteins disappearing in metal-treated cells compared to the control (Table 1). The 10 proteins with the highest copy numbers that appeared in metal-shocked cells (Supplementary Table S4) and the 10 up-regulated ones with the highest Q ratios (Supplementary Table S5) were in most cases products of metal-resistance determinants. Upregulation of the transcription of these genes measured after 10 min [58] showed a corresponding higher copy number of their products after 3 h under these conditions.

The *czc* determinant on plasmid pMOL30 mediated high-level resistance to cobalt, zinc, and cadmium. The RND protein CzcA and the membrane fusion protein CzcB could be found and quantified in CH34 cells under all three cultivation conditions, including EDTA-mediated overall metal starvation (Table 2). This indicated that the CzcCBA complex has an important function in metal homeostasis in *C. metallidurans*, even at low metal concentrations. Nevertheless, the copy numbers of both proteins were clearly upregulated ∼10-fold following metal shock. Unexpectedly, the copy number of the outer membrane factor CzcC was only 46% of that of CzcA (Table 2). Either CzcC was underrepresented or only half of the CzcCBA complexes contained CzcC. CzcC was also strongly upregulated (23-fold) after metal stress, it was not found after EDTA treatment, or was determined only in one set of the control cells. Thus, CzcC may be even more underrepresented in metal-starved or control cells than in metal-shocked cells.

**Table 2.** Products of the plasmid-encoded metal-resistance determinants^a^

Locus tag	Gene	CH34_0	CH34_M	CH34_E	Description
**Plasmid pMOL28:**					
**pMOL28 *mer*: not found *merR, merT, merD,* and *merE***					
Rmet_6346	*merP*	NF	3 935 ± 1757	48	Periplasmic mercury-binding protein
Rmet_6183	*merA*	NF	11 249 ± 2 250	NF	A6UXG5 Mercuric reductase
***chr:* not found *chrZ, chrP, chrF1, chrA1,* and *chrI***					
Rmet_6195	*chrY*	NF	263 ± 152	NF	Q1L9 X 2 Putative uncharacterized protein
Rmet_6197	*chrN*	NF	129 ± 77	NF	Q1L9 X 0 Putative uncharacterized protein
Rmet_6198	*chrO*	NF	727 ± 239	17	Q1L9W9 Putative uncharacterized protein
Rmet_6200	*chrE*	NF	314 ± 199	NF	Q5NUZ8 Superoxide dismutase SodM
Rmet_6201	*chrC*	104	1 318 ± 1 154 (12.7; 1.1)	NF	P17550 Superoxide dismutase (Fe)
Rmet_6203	*chrB1*	NF	649 ± 516	NF	P17552 Protein ChrB
***cnr*: not found *cnrY, cnrC,* and *cnrA***					
Rmet_6206	*cnrX*	44	388 ± 176	NF	P37975 Nickel sensor of the antisigmafactor complex
Rmet_6207	*cnrH*	67	NF	NF	P37978 RNA polymerase sigma factor CnrH
Rmet_6209	*cnrB*	207	1 235 ± 272	NF	P37973 Nickel and cobalt-resistance protein CnrB
Rmet_6211	*cnrT*	11	NF	NF	Q9L3G0 CnrT protein
**Plasmid pMOL30: neither *ncc* nor *sil* products found**					
***pbr*: not found *pbrU, pbrR, pbrA, pbrB/C,* and *pbrD***					
Rmet_5945	*pbrT*	206 ± 156	223 ± 47 (1.1; 0.1)	847 ± 353 (4.1; 1.3)	Q58AJ4 PbrT protein (iron permease FTR1)
***czc* region: not found *flgB, ompP, czcJ, czcD, czcI, czcN,* and *czcM***					
Rmet_5970	*czcP*	31 ± 19	76	NF	Q1LAJ7 Heavy metal translocating P-type ATPase
Rmet_5976	*czcE*	NF	96	NF	Q1LAJ1 Putative uncharacterized protein
Rmet_5977	*czcS*	NF	313 ± 270	NF	Q44007 Sensor protein CzcS
Rmet_5978	*czcR*	67	566 ± 539 (8.5; 0.9)	89 ± 52 (1.3; 0.4)	Q44006 Transcriptional activator protein CzcR
Rmet_5980	*czcA*	95	854 ± 489 (9.0; 1.4)	75 ± 50 (0.8; 0.2)	P13511 Cobalt–zinc–cadmium-resistance protein CzcA
Rmet_5981	*czcB*	146 ± 104	1 509 ± 607 (10.4; 1.9)	113 ± 26 (0.8; 0.2)	P13510 Cobalt–zinc–cadmium-resistance protein CzcB
Rmet_5982	*czcC*	17	391 ± 207 (22.7; 1.8)	NF	P13509 Cobalt–zinc–cadmium-resistance protein CzcC
***cop1*: not found *copV, copT, copM, copK, copD1, copJ, copG, copL, copQ, copE,* and *copW***					
Rmet_6109	*copN*	NF	112 ± 25	NF	Q1LA58 Putative uncharacterized protein
Rmet_6110	*copS1*	NF	467 ± 471	NF	Q58AD4 Sensor protein
Rmet_6111	*copR1*	21 ± 9	322 ± 125	22 ± 8 (1.0; 0.1)	Q58AD5 Two-component regulator
Rmet_6112	*copA1*	NF	1 054 ± 623	NF	Q58AD6 Copper-resistance protein CopA
Rmet_6113	*copB1*	NF	1 559 ± 870	NF	Q58AD7 CopB protein (copper-resistance B)
Rmet_6114	*copC1*	NF	704 ± 120	NF	Q1LA53 Copper-resistance protein CopC
Rmet_6116	*copI*	NF	2 274 ± 982	NF	Q58AE0 Putative oxydoreductase
Rmet_6119	*copF*	195 ± 116	768 ± 701 (3.9; 0.7)	46 ± 21 (0.2; 1.1)	Q58AE3 Heavy metal translocating P-type ATPase
Rmet_6122	*copH*	NF	5 301 ± 2 837	36	Q58AE5 CopH protein
**pMOL30 *mer1*: no products found**					
**pMOL30 *mer2*: not found *merT, merD,* and *merE***					
Rmet_6171	*merR*	117 ± 68	151 ± 102 (1.3; 0.2)	146 ± 38 (1.3; 0.3)	P69413 Mercuric-resistance operon regulatory protein
Rmet_6173	*merP*	NF	3 935 ± 1 757	48	Q58AI1 Periplasmic mercuric ion-binding protein
Rmet_6174	*merA*	NF	249 ± 147	NF	Q1L9Z3 Mercuric reductase MerA

^a^The copy numbers per cell of the products of plasmid-encoded metal-resistance determinants are given for *C. metallidurans* strain CH34 cultivated without added substance (CH34_0), metal-shocked (CH34_M) and metal-starvation conditions (CH34_E) with the mean values and deviations. Numbers without deviations indicate proteins determined just once in the respective triplicate determination. The copy numbers are followed by the ratios Q and the distance value D for the comparison of CH34_M and of CH34_E with CH34_0. These values were not provided when the respective protein could not be measured in CH34_0. NF, not found.

Among the other Czc proteins, the response regulator CzcR was also found under all conditions and was upregulated 8.5-fold following metal stress. Its associated sensory histidine kinase CzcS, the P_IB4_-type ATPase CzcP, and the periplasmic CzcE protein were also found in metal-stressed cells. Other *czc* products could not be determined (Table 2).

*Cupriavidus metallidurans* also has on its chromid an ancient and interrupted *czc_2_* paralog with the gene encoding the central zinc- and cadmium-exporting P_IB2_-type ATPase ZntA in its vicinity (Supplementary Table S2). Interestingly, the CzcC_2_ outer membrane factor was also present in metal-shocked CH34 and AE104 cells although the interrupted *czcB_2_* and the *czcA_2_* gene products could not be identified. This indicated the possibility that CzcC_2_ may also interact with CzcBA to form an alternative transenvelope efflux complex CzcC_2_BA.

Only the membrane fusion protein CnrB of the pMOL28-encoded nickel-resistance determinant *cnr* was found, while the nickel sensor CnrX, and single appearances of the sigma factor CnrH and the inner membrane efflux system CnrT were identified (Table 2). Although CnrB had a similar abundance in metal-shocked CH34 cells as CzcB, neither CnrC nor CnrA could be quantified.

Two more RND-driven transenvelope efflux systems were encoded on the chromid by the *zni/zne* region (Supplementary Table S2). Unexpectedly, the subunits of both transenvelope complexes, ZniCBA and ZneCBA, were found, or were upregulated in their synthesis in EDTA-treated CH34 cells. ZniCBA were also present in metal-shocked and CH34 control cells but were not upregulated when these two conditions were compared. With the exception of ZniA, AE104 control cells revealed that ZniCBA were also present under all conditions tested, and were indeed upregulated in EDTA-treated but not in metal-shocked AE104 cells; however, the copy numbers were lower in EDTA-treated AE104 than in EDTA-treated CH34 cells. This suggests that, unexpectedly, the ZniCBA system has a role under metal-starvation conditions rather than in dealing with metal stress, for instance being part of a cycling process required to route metal cations to their target proteins, as shown for copper [64]. The copy numbers of the Zne proteins were lower than those of the Zni proteins. Zne could possibly support or enhance the function(s) of Zni. Among the remaining components for possible transenvelope exporters of divalent metal cations, only the outer membrane factor NimC and the membrane fusion protein NimB were found, but there was no indication of any regulation in response to metal availability.

The CusCBA components, which are responsible for efflux of the monovalent cations Cu(I) and Ag(I), were only identified in metal-shocked AE104 cells (Supplementary Table S2). The SilCBA components were not found. This suggested that transenvelope efflux of Cu(I) was of lower significance in CH34 than in strain AE104. Indeed, metal-shocked CH34 cells revealed an upregulation in the synthesis of the periplasmic Cu(I) oxidases CopA_1_, encoded on plasmid pMOL30 (Table 2), and its chromid paralog CopA_2_ (Supplementary Table S2), each with similar copy numbers. In addition to CopA_1_, the other pMOL30-encoded proteins CopN, CopB_1_, CopC_1_, CopI, and CopH were also detected, or were found to be upregulated in metal-stressed CH34 cells. Moreover, CopC_2_, two-component regulatory systems CopS_1_ and CopR_1_, CopS_2_ and CopR_2_ were also present in metal-shocked CH34 and AE104 cells. Because CH34 cells contained a variety of factors supporting the synthesis and function of the two periplasmic Cu(I) oxidases, CopA_1_ and CopA_2_, CH34 cells may be able to remove Cu(I) efficiently by oxidation to the less toxic Cu(II), which decreased the need to remove periplasmic Cu(I) by Cus-mediated export. In contrast, export of Cu(I) by Cus seemed to be more important in the plasmid-free strain AE104, which contains only the Cop_2_ system [19].

Six components of the plasmid pMOL28-encoded chromate-resistance determinant were found in metal-treated CH34 cells, but not under the other conditions, with the exception of a single determination of the superoxide dismutase-like ChrC in CH34 control cells (Table 2). Two products of the second, smaller and chromid-encoded *chr_2_* determinant were found in metal-treated CH34 and AE104 cells (Supplementary Table S2). Products of the chromosomal arsenate-resistance and the various mercury-resistance proteins were also upregulated after treatment of the strains CH34 and AE104 with a toxic metal mixture.

GshA and GshB, which are required for glutathione biosynthesis, were not upregulated in synthesis following metal treatment of CH34 or AE104 cells, but proteins required for iron–sulfur cluster biosynthesis, namely IscR, IscA and IscU, were shown to increase in abundance (Supplementary Table S2). This agrees to the fact that iron–sulfur clusters are the primary intracellular targets of copper toxicity [65]. Additionally, the regulatory proteins of the phosphate response, PhoB and PhoU, and the periplasmic phosphate-binding protein PstS of the PstABC import system were upregulated under metal-stressed conditions. This indicated that the synthesis of proteins required for assembly of iron–sulfur clusters and phosphate supply were both responsive to metal stress. Phosphate-stressed polypeptides were possibly induced in response to the presence of arsenate in the challenging metal mix [52, 66, 67].

Among the metal efflux systems of the inner membrane, the chromosomal ZntA was found to be highly abundant, with 3425 ± 2300 copies, but only in metal-stressed CH34 cells (Table 3); the exception were 502 copies identified in unchallenged AE104 cells, but in a single determination (Supplementary Table S2). The cadmium exporter, CadA, could not be identified, nor was the plasmid-encoded lead efflux system PbrA found under any of the conditions tested. While lead was not a component of the toxic metal mixture, cadmium was. The plasmid-encoded P_IB4_-type ATPase CzcP, a high-rate exporter of zinc ions [18], made two single appearances in metal-treated CH34, as well as in untreated CH34 control cells. The Cu(I)–exporter CupA was upregulated in its synthesis in metal-stressed CH34 (Table 3) and in AE104 (Supplementary Table S2) cells, although the latter result was not significant due to a high deviation in the measurements made in metal-shocked AE104 cells. This was also the case for the plasmid-encoded CopF ATPase (Table 3). Like CupA, CopF was upregulated four-fold in metal-treated CH34 cells but the deviation was high. While DmeF could only be determined in CH34 control cells, no upregulation was detected in metal-treated CH34 or AE104 cells, the abundance of this cobalt-exporting CDF protein was two-fold higher in AE104 than in CH34 control cells. The Fe(II) exporter FieF was present in all cells but appeared not to be regulated. RdxI, CtpA1, CnrT, AtmA were each identified once, while CzcD was not found in either cell or under any of the conditions tested (Table 3 and Supplementary Table S2).

**Table 3.** Efflux systems of the inner membrane in *C. metallidurans* strain CH34^a^

Locus tag	Gene	CH34_0	CH34_M	CH34_E	Description
Rmet_4594	*zntA*	NF	3425 ± 2300	NF	Q1LEH0 P_IB2_-type ATPase
Rmet_5970	*czcP*	31 ± 19	76 (2.4; 2.4)	NF	Q1LAJ7 P _IB4_-type ATPase
Rmet_3524	*cupA*	309 ± 184	2640 ± 1113 (8.6; 1.8)	142 (0.5; 0.9)	Q1LHI0 P_IB1_-type ATPase
Rmet_6119	*copF*	195 ± 116	768 ± 701 (3.9; 0.7)	46 ± 21 (0.2; 1.1)	Q58AE3 P_IB1_-type ATPase
Rmet_2046	*rdxI*	155	NF	NF	Q1LLQ1 P_IB1_-type ATPase
Rmet_0198	*dmeF*	221 ± 129	NF	NF	Q1LRZ2 CDF protein
Rmet_3406	*fieF*	209 ± 93	258 ± 150 (1.2; 0.2)	201 ± 116 (1.0; 0.0)	Q1LHU8 CDF protein
Rmet_6211	*cnrT*	11	NF	NF	Q9L3G0 CnrT protein
Rmet_0391	*atmA*	54	NF	NF	Q1LRE9 ABC-type transporter

^a^The copy numbers per cell of the products of various metal-resistance determinants are given for *C. metallidurans* strain CH34 cultivated without added substance (CH34_0), metal-shocked (CH34_M) and metal-starvation conditions (CH34_E) with the mean values and deviations. Numbers without deviations indicate proteins determined just once in the respective triplicate determination. The copy numbers are followed by the ratios Q and the distance value D for the comparison of CH34_M and of CH34_E with CH34_0. These values were not provided when the respective protein could not be measured in CH34_0. NF, not found. Not found in any of the cells were the products of the genes *cadA, pbrA,* and *czcD.* The copper-exporting P_IB1_-type ATPase CtpA1 (Rmet_2379) was found only once with 917 copies in nonchallenged AE104 cells.

Genes with upregulated transcription following metal treatment generally showed a correlation with increased copy numbers of their respective products. Since metal-resistance determinants often encoded membrane-bound products such as metal efflux systems, the low detection efficiency of membrane-bound proteins together with a possible low copy number of these proteins limited their successful determination in many cases. Nevertheless, the proteome of *C. metallidurans* was clearly changed to combat the effects of metal toxicity. Upregulated transcription of genes involved in metal resistance resulted in most cases to an upregulated copy number of the respective gene products.

### Downregulated gene products following metal stress

Following metal shock, *C. metallidurans* not only upregulated the expression of many genes of metal-resistance determinants, but also downregulated expression of many genes encoding ribosomal proteins, proteins involved in the initiation and elongation of translation, transcription, motility, synthesis of hydrogenases, and the components of the F_1_F_0_ ATPase [58]. The corresponding proteins involved in hydrogenase synthesis were only found in CH34 control cells and not in metal-shocked or -starved CH34 cells (Supplementary Table S2). Among the chemotaxis proteins, 26 were not found at all and only 12 were identified in CH34-untreated control cells, half of which were only detected in a single sample. These six proteins were either not downregulated in abundance in metal-treated CH34 cells or appeared only as single measurements under this condition. The levels of the components of the F_1_F_0_ ATPase were not regulated under any of the conditions or in either strain tested (Supplementary Table S3). The components of the RNA polymerase, including the various sigma factors, were also not regulated in CH34 cells after metal shock, with the exception of a 50% reduction in RpoB and a 40% reduction in the termination factor Rho; however, the anticipated upregulation of the sigma factor associated with *cnr* expression, CnrH, was noted. The abundance of the starvation sigma RpoS did not change under these any of the conditions. The total number of ribosomal proteins was 444 000 ± 159 000 in CH34 control cells and 80% of this number, 356 000 ± 141 000, was still found in metal-shocked cells (Supplementary Table S2). This demonstrated that the downregulated transcriptional activity of these genes after 10 min did not manifest as a measurable decrease in the amount of the gene products after 3 h. The cells were able to adapt to the altered conditions within 1.5 cell duplications.

### Metal starvation

About 200 proteins were either upregulated in their abundance or appeared in metal-starved CH34 cells, and about 400 were down-regulated or were no longer detectable (Table 1). The numbers of upregulated proteins or proteins making an appearance in metal-starved AE104 cells were ∼300 and 200, respectively. Among the proteins making an appearance in metal-starved CH34 cells, and which had the highest copy number, were the TonB-dependent siderophore receptor Rmet_0837, the periplasmic binding protein HmuT of an ABC-type importer, and Rmet_1115 involved in siderophore biosynthesis (Supplementary Table S4). These proteins also appeared in metal-starved AE104 cells. In CH34 cells, the proteins that were no longer detectable included those involved in the synthesis of the soluble hydrogenase, while in strain AE104 included were the three systems ZntA, DmeF, and CtpA1 for Zn, Co, and Cu ions efflux, respectively (Supplementary Table S4); however, it should be emphasized that all three were only identified in one of the AE104 control cell samples.

The most strongly upregulated proteins in metal-starved CH34 cells were the Zni and Zne components, the cysteine synthase CysK, other components involved in siderophore biosynthesis and ExbB1, which was needed to drive TonB-dependent transport processes (Supplementary Table S5). CysK, a TonB-dependent outer membrane receptor, and ZniC were also upregulated in strain AE104 under the same condition.

These results indicated that uptake of iron was primarily affected in EDTA-treated CH34 cells. Indeed, the proteins involved in siderophore biosynthesis were significantly upregulated or appeared in metal-starved CH34 and AE104 cells (Supplementary Table S2). The TonB-dependent outer membrane receptor Rmet_0123 was downregulated in metal-shocked but upregulated in both strains upon metal starvation. The sigma factor RpoI, which controlled expression of the siderophore biosynthesis cluster, appeared in metal-starved CH34 cells, as did its membrane-bound anti-sigma factor, RsiA. Moreover, the anti-sigma factors RsjA and RskA of the sigma factors RpoJ and RpoK, respectively, both of which are related to RpoI, were also identified in metal-starved CH34 cells, although their cognate sigma factors were not found. The membrane-bound iron importer FeoB was significantly upregulated in metal-starved CH34 cells, but was also present in metal-shocked CH34 as well as the untreated control cells (Table 4). FeoB was also present in AE104 cells in slightly higher copy numbers in comparison to CH34 cells under the same conditions, but the differences were not significant (Table 4). The associated small (100 aa) FeoA protein could also be quantified in metal-starved CH34 and AE104 cells (Table 4). In contrast to the proteomic response measured after 3 h, transcription of the respective genes was not upregulated after 10 min [58].

**Table 4.** Metal uptake systems^a^

Locus tag	Gene	Control (Q, D)	Metal-shocked (Q, D)	Metal-starved (Q, D)	Description
**Strain CH34**					
Rmet_3052	*corA1*	394 ± 242	NF	NF	Q1LIV2 Mg and Co transport protein CorA1
Rmet_0036	*corA2*	391	NF	NF	Q1LSF4 Mg and Co transport protein CorA2
Rmet_3287	*corA3*	162 ± 96	180; (1.1; 0.2)	108 ± 63 (0.7; 0.3)	Q1LI67 Mg and Co transport protein CorA3
Rmet_1973	*pitA*	NF	NF	NF	Q1LLX4 Phosphate transporter
Rmet_5396	*mgtA*	29 ± 17	32 (1.1; 0.2)	26 ± 15 (0.9; 0.1)	Q1LC71 ATPase, E1–E2 type
Rmet_0549	*zntB*	82 ± 22	79 (1.0; 0.2)	68 ± 19 (0.8; 0.3)	Q1LQZ1 Mg and Co transport protein ZntB
Rmet_5890	*feoB*	212 ± 69	292 ± 75 (1.4; 0.6)	**1176 ± 699 (5.6; 1.3)**	Q1LAS7 Ferrous iron transport protein FeoB
Rmet_5891	*feoA*	194	NF	246 ± 142 (1.3; 0.4)	Q1LAS6 Ferrous iron transport protein FeoA
**Strain AE104**					
Rmet_3052	*corA1*	371	NF	NF	Q1LIV2 Mg and Co transport protein CorA1
Rmet_0036	*corA2*	195 ± 128 (0.5; 1.5)	NF	229 (1.2; 0.3)	Q1LSF4 Mg and Co transport protein CorA2
Rmet_3287	*corA3*	67 (0.4; 1.0)	41 ± 26 (0.6; 1.0)	127 ± 77 (1.9; 0.8)	Q1LI67 Mg and Co transport protein CorA3
Rmet_1973	*pitA*	209	NF	NF	Q1LLX4 Phosphate transporter
Rmet_5396	*mgtA*	22 (0.8; 0.4)	NF	28 ± 17 (1.3; 0.4)	Q1LC71 ATPase, E1–E2 type
Rmet_0549	*zntB*	80 ± 52 (1.0; 0.0)	**186 (2.3; 2.0)**	51 ± 30 (0.6; 0.3)	Q1LQZ1 Mg and Co transport protein ZntB
Rmet_5890	*feoB*	311 ± 118 (1.5; 0.5)	443 ± 432 (1.4; 0.2)	588 ± 216 (1.9; 0.8)	Q1LAS7 Ferrous iron transport protein FeoB
Rmet_5891	*feoA*	NF	178	125 ± 72	Q1LAS6 Ferrous iron transport protein FeoA

^a^The number of the products per cell of the respective gene with deviations, plus the comparisons CH34 with metal mix to without, and CH34 with EDTA to without, same for strain AE104 and for nonchallenged control cells AE104/CH34. The ratios Q and the distance values follow these numbers in parentheses. Single values indicate a result only in one out of the three determinations. NF is ‘not found’ in any of the three replicates. Comparisons to NF values were not done. Bold-faced Q ratios with D >1 and at least two-fold up- und downregulation of the abundance.

**Table 5.** Transcriptomic data for the gene-encoded proteins with different changes in the sense-to-antisense ratios^a^

		Before the change			After the change			Comparison			
Locus tag	Gene	Mean	Mean_AST	S/AS	MEAN	Mean_AST	S/AS	Comp	Q_sense	Q_AST	QS_AS
**Data with QS_AS ratios >50**											
Rmet_3525	*cupC*	184 ± 37	334 ± 45	0.55	7 912 ± 912	255 ± 28	31.0	CM0+	42.9	0.77	56.1
Rmet_0333	*arsR*	47 ± 4	3 ± 2	14.2	17 327 ± 1 563	20 ± 3	852	AM0+	366	6.10	60.0
Rmet_0333	*arsR*	61 ± 3	5 ± 3	13.0	20 485 ± 2 242	23 ± 2	878	CM0+	338	5.00	67.5
Rmet_0331	*arsC2*	11 ± 1	3 ± 1	3.40	5 969 ± 277	20 ± 3	294	AM0+	527	6.10	86.3
Rmet_3525	*cupC*	145 ± 19	306 ± 38	0.48	6 767 ± 1 554	153 ± 17	44.3	AM0+	46.6	0.50	93.2
Rmet_3620	*degP*	28 ± 1	105 ± 10	0.26	3 680 ± 424	116 ± 8	31.7	CM0+	133	1.10	121
Rmet_0123		6 ± 1	102 ± 12	0.06	132 ± 20	1.7 ± 1.1	79.0	AE0+	23.2	0.02	1 417
**Data with QS_AS ratios between 1.2 and 20 (selected data points)**											
Rmet_5319	*zniA*	57 ± 3	4 ± 1	13.1	147 ± 3	3 ± 1	49	CE0+	2.6	0.69	3.7
Rmet_5320	*zniB*	116 ± 8	29 ± 3	3.94	291 ± 56	44 ± 4	6.6	CE0+	2.5	1.49	1.7
**Data with QS_AS ratios <0.25**											
Rmet_5673	*copS2*	5 ± 1	Not found	39 ± 3	43 ± 4	0.91	CM0+	7.38			
Rmet_5321	*zniC*	46 ± 4	1 ± 1	46.3	110 ± 7	156 ± 9	0.71	AE0+	2.37	157	0.02
Rmet_1195	*bfrB*	315 ± 5	157 ± 20	2.01	110 ± 9	681 ± 93	0.16	AM0−	0.35^b^	4.35	0.08
Rmet_1026	*iscU*	545 ± 36	2.3 ± 0.6	233	1 724 ± 72	65 ± 9	26.5	AM0+	3.17	27.9	0.11
Rmet_0506	*purK*	125 ± 24	0.3 ± 0.6	375	62 ± 9	1.3 ± 0.6	46.2	AM0−	0.49 ^b^	4.00	0.12
Rmet_0970	*ggt*	235 ± 17	2.3 ± 0.6	101	37 ± 3	2.7 ± 1.1	14.0	AM0−	0.16 ^b^	1.14	0.14
Rmet_1026	*iscU*	506 ± 17	4 ± 0	127	1 728 ± 146	63 ± 5	27.3	CM0+	3.41	15.8	0.22
Rmet_1027	*iscA*	556 ± 59	4 ± 0	139	2 015 ± 421	63 ± 5	31.8	CM0+	3.62	15.8	0.23

^a^The published transcriptomic data [58] for genes with strongly increased (>50) or decreased (<0.25) sense-to-antisense RNA ratios in the comparisons CH34 metal-shocked to the control (CM0; CMO+ up- and CM0− down-regulated), same for AE104 (AM0), metal-starved CH34 cells to the control (CE0) and the same for strain AE104 (AE0). The abundances as NPKM values of the RNAs before and after application of the changed condition are listed, Mean for sense RNA, Mean_AST for antisense RNA, S/AS for the ratio of sense-to-antisense RNA plus the changes of the sense, antisense abundances and that of the sense-to-antisense ratios.

^b^The ratios Q are given while the associated figures are using the 1/Q values. Down-regulated genes on a gray field.

Of the other metal uptake systems, ZupT, HoxN, and MgtB were not found and the metal inorganic phosphate importer PitA was identified just once in AE104 control cells (Table 4). The MgtA P-type Mg/Ca importer was present under five of the six conditions, but its synthesis was not regulated in response to metals or a lack thereof. The four representatives of the CorA-type uptake systems for Mg(II) and other divalent cations were found in some of the cells but could not be detected in all of them. Only ZntB, which might be an importer or exporter of Zn ions, showed a 2.3-fold upregulation in metal-shocked AE104 cells, but this result was based upon only a single determination in these cells. Otherwise, FeoB was the only upregulated metal uptake system.

Proteins involved in either uptake of phosphate by the PstABC importer or synthesis or degradation of polyphosphate were not changed in abundance under any condition (Supplementary Table S2), nor were GshA and GshB, which are required for glutathione biosynthesis. Concerning the biosynthesis of iron–sulfur clusters, the abundance of the Isc proteins did not change under metal starvation conditions, with a downregulation of 40% for the cysteine desulfurase IscS being detected in metal-starved CH34 cells. While the important zinc importer, ZupT, could not be found, the remaining proteins produced under control of the zinc uptake regulator Zur were identified, including Zur. CobW2 and CobW3 were present under all conditions. The abundance of CobW3 was not changed—a 2.2-fold higher level of the zinc storage protein CobW2 was noted in metal-starved CH34 cells. The copy number of CobW2 in metal-starved AE104 cells was also slightly higher than in AE104 and in CH34 control cells but the differences were not significant. The third CobW protein, CobW1, which is produced only under strong zinc starvation conditions, was identified in metal-starved CH34 and AE104 cells, and this was also true for the product of the second gene of the *cobW1* operon, the GTP (Guanosine triphosphate) cyclohydrolase FolE_IB2, which is a metal-promiscuous enzyme [68]. The two paralogs of FolE_IB2, the metal-promiscuous FolE_IB1 and the zinc-dependent FolE_IA, were present under all conditions but showed no upregulation of their abundance, except for a 2.1-fold increase in FolE_IB1 levels in metal-treated AE104 cells.

These findings demonstrated that *C. metallidurans* primarily reacted to EDTA-mediated metal starvation by upregulation of the capacity to import iron ions directly, or associated with its siderophore. This seemed to be sufficient to supply iron to the Isc iron–sulfur cluster biogenesis apparatus. An upregulation of the storage capacity for zinc and for a Zn-independent GTP hydrolase involved in folate biosynthesis were additional adjustments of the proteome to EDTA-mediated zinc starvation conditions.

### Relationship between protein abundance and associated transcripts

To determine the relationship between protein abundance and the expression levels of its associated sense transcript mRNA and antisense asRNAs, we measured the abundance in proteins per cell encoded by a gene and matched this with the abundance of the respective mRNA in NPKM (nucleotide activities per kilobase of exon model per million mapped reads, a measure of RNA abundance), as published [58]. This was done for all six conditions (strain CH34 or AE104, control, metal-shocked, and -starved cells). In a second step, the proteome mRNA data points were sorted into six groups, which now depended on the abundance of the asRNA, given in NPKM as published [58]. These groups included AST0, which were genes/transcripts with no associated asRNA, as the first group, whereas the groups AST1 and AST5 were between or above the boundaries when NPKM = 3, 10, 30, or 100 (groups AST1 to AST5).

For all conditions, the asRNA-grouped protein abundances were plotted against that of their mRNA in a double-log_10_ plot (Supplementary Fig. S3). Although the distribution of the data points was scattered, the protein abundances increased with measured increases of their cognate sense RNA. In all comparisons, the data points of the group AST1, associated with low asRNA abundances (green in S3), represented genes expressed on a low level and subsequently a low copy number of the corresponding gene product. On the other hand, a group with a high, but not the highest, asRNA abundance (AST4, red data points in S3, 30 < NPKM ≤ 100) was associated with strong expression events and subsequently high copy numbers of those gene products. This demonstrated that a change in the transcriptome at the onset of a cellular adaptation process indeed resulted in a subsequently altered proteome composition.

**Figure 1.**
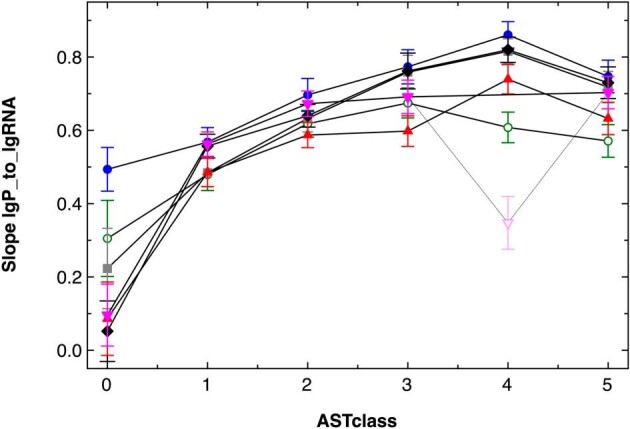
The abundance of antisense RNAs influenced the protein yield from the associated sense RNA. The abundance of proteins (copy number per cell) was plotted as decadic logarithm against that of the abundance of its transcript (NPKM values as published [58]). Closed circles are CH34 control cells, open circles metal-, squares EDTA-treated cells of strain CH34. Diamonds are AE104 control cells, triangles metal- and inverted triangles EDTA-treated AE104 cells. Data points were grouped according to the abundance (NPKM values as published [58]) of the respective asRNA into six groups: no asRNA (0), NPKM ≤3 (1), 3 < NPKM ≤ 10 (2), 10 < NPKM ≤ 30 (3), 30 < NPKM ≤ 100 (4), and NPKM > 100 (5). A linear curve fit was performed for the six groups (Supplementary Fig. S3) to the function lg_10_(protein) = lg_10_(a) + lg_10_(b) × lg_10_(senseRNA). Shown here is the slope lg_10_(b) for the six asRNA groups per condition. The color code has no meaning and was used for contrast. The pale inverted triangle was a slope outside of the remaining data.

For all asRNA groups and conditions, a linear curve fit was employed, which usually had low regression coefficients ∼50% due to the large scattering of the data points. When the resulting functions were plotted (Fig. 1, Supplementary Fig. S3, and Supplementary Table S6), the slopes of the functions increased with the asRNA abundances. The functions had the form lg_10_(protein) = lg_10_(a) + lg_10_(b) × lg_10_(senseRNA) so that the protein abundance P = a × b^lg(sRNA)^. The abundance of the sense RNA was between 1 and 10 000, so that lg_10_(sRNA) was between 0 and 4. For all conditions, the a values decreased from class AST0 to AST4 whereas the slope (b values) increased. This indicates that the influence of the mRNA abundance on the protein abundance increased with the asRNA abundance. Exceptions were the data points in class AST5 with NPKM >100 for the asRNA. They displayed higher a but lower b values again, so that here a high asRNA abundance negatively affected the protein-to-mRNA ratios. These data indicated a global positive effect of asRNAs on gene expression at low asRNA abundances, but a negative effect at high values. On a global scale, asRNAs enhanced the copy number of the gene products at low abundances but seem to have a destabilizing function at high asRNA abundances. Antisense transcripts thus influenced the proteome composition. 

### Influence of transcriptome on proteome changes

**Figure 2.**
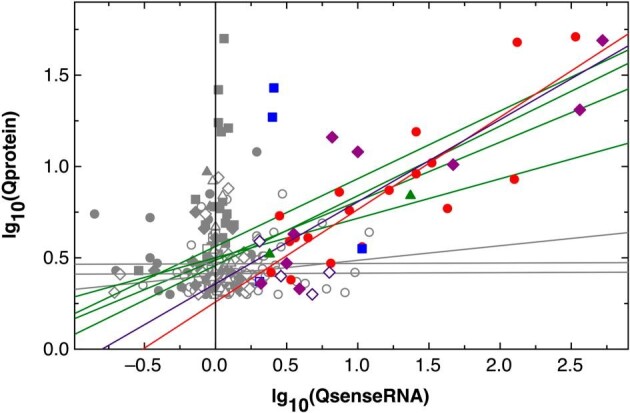
Changes in abundance of proteins correlated with changes in abundance of the associated sense RNA. The 327 proteins with a significantly changed abundance following metal-stressed or starvation in CH34 and AE104 cells were plotted against the changes of the associated sense RNA. Filled symbols represent upregulated protein copy numbers, open symbols the inverse ratio Q of downregulated proteins. Circles are the comparison of metal-shocked CH34 to the control (CM0), squares metal-starved CH34 cells to the control (CE0), diamonds metal-shocked AE104 cells to the control (AM0), and triangles metal-starved AE104 cells (AE0). Colors indicate data points with significant changes (Q ≥2 for up- and 1/Q ≥2 for downregulated RNAs, D >1). CM0 red, CE0 blue, AM0 purple, and the two values for AE0 in green. Gray symbols are the results with significant changes in the proteome but not the transcriptome. The lines are linear curve fittings for the subsequent functions: CM0+, all data: lg10(Qprotein) = 0.487 ± 0.034 + 0.323 ± 0.042 × lg10(QsenseRNA), *R*^2^ = 74.8% **CM0+, Qs ≥2: lg10(Qprotein) = 0.258 ± 0.100 + 0.506 ± 0.077 × lg10(QsenseRNA), *R*^2^ = 75.5%** *CM0−, all data: lg10(Qprotein) = 0.406 ± 0.017 + 0.079 ± 0.049* × *lg10(QsenseRNA), R^2^ = 19.0%* CE0+, all data: lg10(Qprotein) = 0.562 ± 0.042 + 0.371 ± 0.233 × lg10(QsenseRNA), *R*^2^ = 20.4% *CE0−, all data: lg10(Qprotein) = 0.413 ± 0.017 + 0.003 ± 0.246* × *lg10(QsenseRNA), R^2^ = 2.2%* AM0+, all data: lg10(Qprotein) = 0.459 ± 0.044 + 0.381 ± 0.049 × lg10(QsenseRNA), *R*^2^ = 85.0% **AM0+, Qs ≥ 2: lg10(Qprotein) = 0.358 ± 0.143 + 0.449 ± 0.098** × **lg10(QsenseRNA), R^2^ = 86.7%** *AM0−, all data: lg10(Qprotein) = 0.467 ± 0.032 + 0.002 ± 0.102* × *lg10(QsenseRNA), R^2^ = 0%* AE0+, all data: lg10(Qprotein) = 0.500 ± 0.079 + 0.261 ± 0.176 × lg10(QsenseRNA), *R*^2^ = 39.8% Upregulated items in the comparisons are indicated by a ‘+’ and green lines, downregulated (1/Q values) by a ‘*−*’ and gray lines. For AE0*−, R*^2^ was <null and this function is not given. Functions in italic letters have regression coefficient <20%. The bold-faced equations are those for the data points with QsenseRNA ≥2 with CM0+ in red and AMO+ in purple.

The data points resulting from the plot of a protein abundance to that of its sense RNA were scattered (Supplementary Fig. S3). This may mirror the fact that the abundance of a protein depends on many factors, for instance that of the associated mRNA, translation initiation and elongation efficiency, degradation and dilution by growth of the cell. A comparison of the ratios Q of the up- or downregulation of the abundance of a protein and its associated sense RNA may negate (or nullify) the influence of the other factors and highlight that of the sense RNA abundance. Moreover, great changes in asRNA abundance should be reflected in linearly correlated changes in inhibition rate of the sense RNA [69]. Thus, the Q values for the regulation of the significantly regulated 327 proteins (Table 1) were plotted against the changes of their associated sense RNAs, using 1/Q values for downregulated proteins (Fig. 2, gray and colored symbols). The comparison of control cells of AE104 with those from strain CH34 was omitted from this analysis because asRNA-dependent regulatory events vary between bacterial strains [70].

**Figure 3.**
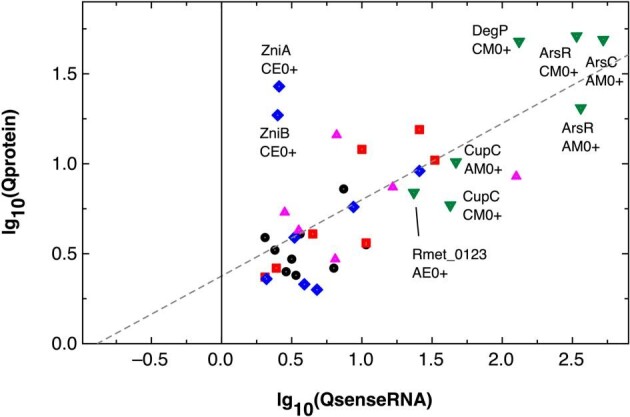
Changes in abundance of proteins correlated with changes in abundance of the associated sense RNA. The figure shows the same data points as that in Fig. 1 but uses another symbol and color code to group these points according to the associated changes in the sense-to-antisense ratios QS_AS. Group 1, no QS_AS ratio or QS_AS <0.25 (black circles); group 2, 0.5 < QS_AS < 1.2 (red squares); group 3, 1.2 ≤ QS_AS < 4 (blue diamonds); group 4, 4 ≤ QS_AS < 20 (magenta triangles); and group 5, QS_AS > 50 (green inverted triangles). Dashed lines come from the fitting of all values (small gray dot within the symbols), lg_10_(Qprotein) = 0.376 ± 0.084 + 0.424 ± 0.069 × lg_10_(QsenseRNA), *R*^2^ = 72.1%, or Qprotein = 2.38 × 2.66^QsenseRNA^. Data points for group 5 and two *zni* data points from other groups are labelled with the protein and the respective comparison.

Seven proteins were upregulated, despite showing no or only a very low upregulation of their sense RNA (gray filled squares in parallel with the vertical zero line in Fig. 2) but only for two proteins was the upregulation of the sense RNA abundance significant (blue filled squares). These were ZniA and ZniB with ∼2.5-fold upregulation of the sense RNA resulting in a >10-fold higher protein abundance in EDTA-treated CH34 cells (Fig. 3). This highlighted the importance of the ZniCBA transenvelope complex in handling metal starvation conditions.

For all data points, the double-log_10_ plots followed linear functions with regression coefficients of 75% and 85% for genes upregulated following metal stress in strain CH34 or AE104, respectively (Fig. 2). The regression coefficients for upregulated genes in metal-starved CH34 or AE104 cells were 20% and 40%, and much lower than those for upregulated metal-stressed cells, for both strains. For downregulated genes, the regression coefficients were 19% for metal-stressed CH34 cells, and close to, at or below zero for genes down-regulated in metal-starved or unchallenged CH34 cells. However, the lower abundance of the downregulated proteins did not correlate with the downregulation of the associated mRNAs.

To analyze the effect of antisense RNAs on the effect of changed sense RNA abundances and consequently altered protein abundances, only the data points with both significantly changed protein and sense RNA abundances were further considered (Table 1 and Fig. 2, colored data points). This resulted in 37 data points, mostly from upregulated metal-shocked CH34 and AE104 cells. The curve fittings of these double-log plots gave regression coefficients similar to those from the plot using all data points. This was the consequence of the strong influence of highly upregulated protein abundance on the sense RNA plots and the fact that all data points with Q values below 2 (log_10_(2) = 0.301) were below the threshold so that all functions started in the region of the data point 0.3/0.3 (Fig. 2).

The 37 data points were subsequently regrouped into 5 clusters with increasing ratios of the sense to the associated antisense RNAs (Fig. 3, Table 5). Two clusters of data points were separated from each other. A strong increase (>50-fold) of the sense RNA compared to its antisense RNA represented highly upregulated sense RNAs, leading to an increase in the abundance of the gene products (Fig. 3, green inverted triangles). A second group included data points with decreased sense-to-antisense ratios (≤0.23-fold) and upregulation of the sense RNAs and their product on a low level (Fig. 3, black closed circles). The other groups, which contained events with no change in the sense-to-antisense ratios (red squares), a small (blue diamonds) or medium (magenta triangles) increase in the sense-to-antisense ratios, fell between these two groupings.

Despite the differences in the changes of the sense-to-antisense ratios, the data points could be fitted to a linear function with a regression coefficient of 72% (Fig. 3). This means that the abundance of the sense mRNA had a stronger effect on the subsequent increase or decrease of the protein copy number than that of the sense-to-antisense ratios. Group 1 of these data points contained gene products without asRNA or with a >four-fold decrease of the sense-to-antisense ratios, meaning much more asRNA relative to the associated sense RNA (Fig. 3, black dots). A stronger impact of the asRNA abundance would lead to the appearance of two additional clusters in these data points, one generated in the absence of asRNA, in which no stabilizing effect could be exerted by the asRNA molecule on the already destabilizing sense RNA, and the second, in which there is an increasing effect of the asRNA on its sense RNA, whether one of stabilization or destabilization. These data sets with a decrease in the sense-to-antisense ratios (Fig. 3, black circles) contained data points associated with downregulation of three genes: *purK* encoding a subunit of the phosphoribosylaminoimidazole carboxylase, *ggt* encoding a γ-glutamyl transferase, and *bfrB* encoding a bacterioferritin (Table 5). The upregulated genes encoded the chromid-located copper-sensing histidine kinase CopS_2_, ZniC for the ZinCBA transenvelope efflux system and components of the Isc system for the synthesis of iron–sulfur clusters.

Some of the data points were simultaneously associated with the strongest increase in the sense-to-antisense ratios, an increase in abundance of sense RNA and of the copy numbers of the respective gene product (Fig 3, green inverted triangles). Found in metal-shocked conditions were the regulator of arsenate-resistance ArsR, appearing in CH34 as well as AE104 cells, while the arsenate reductase ArsC appeared only in AE104, but the copper chaperone CupC was present again in both strains. The protease DegP was only found in metal-shocked CH34 cells and representative for EDTA-starved AE104 cells was an outer membrane TonB-dependent receptor Rmet_0123 that is encoded downstream of the *zur-cobW2-cobW3* genes in the same operon Op0032r.

Two data points stood out that were above the function that represented the double-log_10_ plot (Fig. 3, blue diamonds). They showed a higher abundance of their copy number in comparison with that of the sense RNA. These were ZniA and ZniB in EDTA-treated CH34 cells. Such an effect may be due to a stabilizing effect, for instance by an asRNA.

## Discussion

### Changes in the proteome of *C. metallidurans* following metal stress

Metal resistance in *C. metallidurans* CH34 wild type is mediated by resistance determinants on the two plasmids pMOL28 and pMOL30 [71], resulting in upregulation of these genes following metal shock, and indeed, the gene products can be found in the proteome of this bacterium. Prominent are the components of the transenvelope efflux complexes CzcCBA (cobalt, zinc, and cadmium resistance) and CnrCBA (cobalt and nickel resistance), and proteins involved in copper and chromate resistance. The CzcCBA proteins were also present in control cells cultivated without added metals so that Czc has also a function in metal homeostasis in the absence of high metal concentrations.

**Figure 4.**
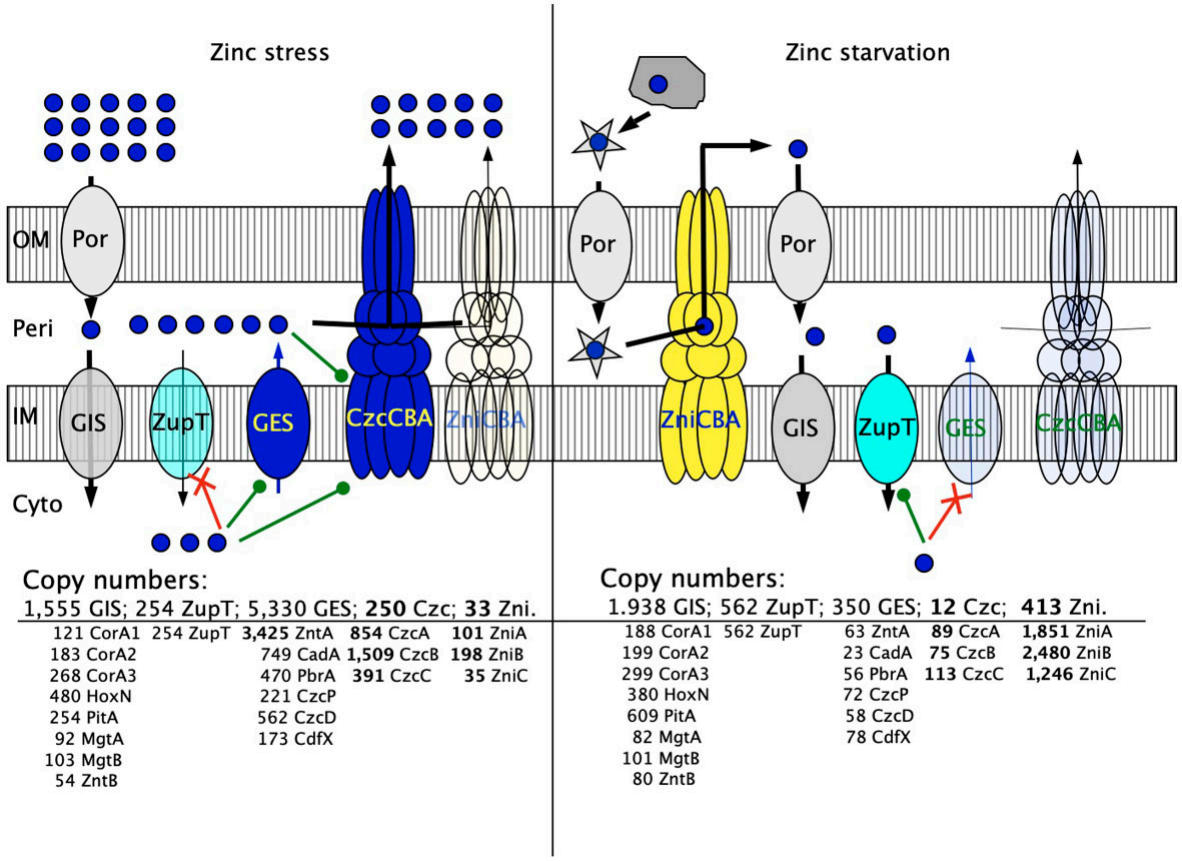
Model of Zn(II) transport under stress and starvation conditions. Under conditions of zinc (small circles) stress, Zn(II) is imported into the periplasm by porins and either immediately exported by 250 CzcCBA systems to the outside, or imported by 1555 general import systems (GIS) or 254 ZupT into the cytoplasm. Due to the flow equilibrium [25], Zn(II) is exported back into the periplasm by 5330 general export systems (GES) such as ZntA. Under zinc-starvation conditions (right hand), hypothetical zinc-containing particles (gray irregular field) might generate zinc-containing fragments or species (stars), which are also imported into the periplasm. A total of 413 ZniCBA complexes might release the zinc ion from these fragments by export to the outside. Subsequently, Zn(II) is reimported into the periplasm and further on into the cytoplasm. Below, the copy numbers of the transport proteins are shown, bold-faced when measured, otherwise the numbers are generated from the transcript abundance using the functions generated in Supplementary Fig. S3. The arrows indicate transport, lines with crosses possible downregulatory, lines with balls upregulatory effects of transport activities. Lower intensities of the colors of the transport systems indicate a lower abundance of the respective proteins.

Transenvelope efflux complexes are composed of an RND (resistance, nodulation, cell division protein family [72, 73]) inner membrane protein that extends into the periplasm, an outer membrane factor OMF that also reaches into the periplasm [74], and a membrane fusion MFP or adaptor protein [72, 75, 76], named CzcA, CzcC, and CzcB, respectively. For the CusCBA copper-exporting system from *Escherichia coli*, a subunit composition CusC_3_B_6_A_3_ was determined [15]. This subunit ratio was prominent among the RND-driven transenvelope efflux systems for metals and organic substances [77]. In our data set, the ratio of CzcA to CzcB was 1:1.8 so that a CzcA_3_B_6_ complex structure could be concluded but the OMF CzcC was only present in metal-challenged CH34 cells in a ratio of CzcA:CzcC = 1:0.46 (Table 2, Fig. 4). Since membrane-integral proteins were not efficiently quantified in the proteomic approach used, this could be the consequence of an underrepresentation of the OMF with its membrane-integral hydrophobic β-barrel. The related OMF CnrC was not even detectable in metal-shocked CH34 cells, whereas CnrB was present in a similar abundance as CzcB, 1235 ± 272 versus 1509 ± 607 copies per cell, respectively, suggesting potentially 200 copies of the CnrC_3_B_6_A_3_ and 250 copies of the CzcC_3_B_6_A_3_ complexes per cell.

The chromid-encoded transenvelope efflux system ZniCBA was interestingly found with increased abundance in EDTA-treated CH34 cells, indicating some role in metal ion supply and not resistance (Fig. 4). By looking at the abundance of the three proteins, a subunit composition of ZniC_3_B_6_A_4.5_ with ∼413 copies per cell could be derived from the number of the B subunits. Adjacent to the *zni* region on the chromid is the *zne* region. The related ZneCBA complex was also upregulated under metal-starvation rather than under metal-resistance conditions. The subunit ratio of ZneA:ZneB was 1:1.5, with ZneC being present in lower copy numbers as determined from the result of a single determination in these cells. The total number of ZneCBA complexes was 77 per cell and thus much lower than that of the ZniCBA complexes. Abundance of ZniCBA and ZneCBA complexes was also upregulated in metal-starved AE104 cells, leading to ∼76 and 3 complexes, respectively (Supplementary Table S2). Zni and Zne could have a specific function in periplasmic metal homeostasis under metal-starvation conditions in CH34 cells with their sophisticated periplasmic metal-resistance components.

Appearance of CzcCBA and an even 12-fold upregulation of ZniCBA in metal-starved cells was not expected (Fig. 4). One explanation would be that CzcCBA is continuously present to counteract any sudden increase in Co(II), Zn(II), and Cd(II) concentrations, but this does not explain the upregulation of ZniCBA. ZniA is an export system of divalent transition metal cations as judged by a conserved motif (‘DFG—D—EN’) in the membrane-integral α-helix, which is essential for import of protons into the cytoplasm and subsequently proton-driven export of metal cations from the periplasm to the outside [12, 13]. ZniCBA is thus a transenvelope protein complex for export of divalent transition metal cations. The difference between CzcCBA and ZniCBA is the presence of additional metal-binding sites in CzcA and CzcB, which may allow a flux control of the efflux activity of CzcCBA driven by the cytoplasmic or periplasmic Zn(II) or Co(II) concentrations [12, 20, 78], with an additional control of this activity by the periplasmic protein CzcI [33]. Under certain metal-starvation conditions, CzcCBA may export its substrate metal cations only with a low rate and ZniCBA may be needed to compensate for the loss of transport activity by CzcCBA.

In marine environments, zinc is found in inorganic pools that include biogenic silica, clays, and various metal oxides [79]. *Cupriavidus metallidurans* most likely encounters such zinc speciations also in its environment. Such particular zinc-containing substances may generate fragments or species able to cross the outer membrane, for instance driven by TonB-dependent outer membrane proteins. It could be hypothesized that CzcCBA, ZniCBA, and other RND-driven transenvelope systems may have a second function in addition to mediating metal resistance: mobilization of essential transition metal cations from various inorganic and organic complexes (Fig. 4). The cation would be sequestered by the efflux system, exported to the outside, able to re-enter the periplasm as ‘free’ ions just complexed by water, and subsequently imported into the cytoplasm. That way, a cycling of metal ions occurs that may assist their allocation to a target protein [64]. Such a function of CzcCBA or ZniCBA as a substitute in release of Zn(II) or Co(II) from organic or inorganic complexes would explain the upregulation of the copy numbers of the ZniCBA components under those metal-starvation conditions that lead to downregulated activity of CzcCBA by flux control [78].

Metal-shocked *C. metallidurans* cells induced a large variety of metal-resistance genes, and for many of them, the gene products could be found in the proteome (Supplementary Table S2). Following metal shock, *C. metallidurans* also downregulated genes for ribosomal proteins, proteins involved in the initiation and elongation of translation, transcription, motility, synthesis of hydrogenases, and the components of the F_1_F_0_ ATPase [58]. A downregulation of the respective gene products after 1.5 cell duplications, however, was not measured (Supplementary Table S2). The abundance of a protein depends not only on *de novo* synthesis but also on degradation and dilution by growth. With a doubling time of *C. metallidurans* of ∼4 h and a period of 3 h between challenge of the cells and their harvest, growth of the cell should dilute the abundance of a protein down to 60%. To keep the abundance of a protein constant during growth and reduce its subsequent dilution, ∼40% have to be newly synthesized during the period of 3 h. A decrease in the abundance of the mRNA by half would consequently mean that only 20% of a given protein would have been synthesized, resulting in a coverage of 80% on the protein abundance after the experimental period of 3 h compared to time zero. Indeed, 80% of the total number of ribosomal proteins were found in metal-challenged CH34 cells compared to the control, but with respect of a general deviation of 0.4-fold of the proteome determination, this difference is most likely purely a coincidence. This means that the downregulation of all these genes at the onset of a metal shock did not ultimately lead to a lower protein content, indicating that the cells had adjusted to the high metal concentration, reached metal homeostasis again, and proceeded with growth after 1.5 cell duplications.

These results demonstrated that upregulated genes in metal-challenged *C. metallidurans* cells indeed led to an increased abundance of proteins involved in metal resistance. Moreover, these increased abundances were interrelated at the transcriptional and translational levels. This could be anticipated but now has been demonstrated.

### Connecting the proteome and the handling of zinc cations in *C. metallidurans*

Pulse-chase experiments demonstrated continuous import and export of zinc ions into *C. metallidurans* cells [25]. In standard Tris-buffered mineral salt medium containing 200 nM Zn(II), the cells rapidly accumulated radioactive ^65^Zn as well as isotope-enriched stable ^67^Zn, and exported these ions quickly when chased with 100 µM nonradioactive or nonenriched zinc. In the wild-type *C. metallidurans* CH34, which contained the plasmid-encoded *czc* determinant, zinc import was strongly reduced, especially when the cells were preincubated in the presence of 100 µM Zn(II) [25]. In agreement with previous results [80, 81], it was concluded that CzcCBA was responsible for this decreased accumulation of zinc. The proteomic data supported this conclusion. As judged from the number of CzcB subunits, nonchallenged CH34 cells contained on average 24 CzcCBA complexes, metal-induced cells 250, and metal-starved cells only 12 complexes (Table 2, Fig. 4). The plasmid-free strain AE104 does not contain *czc* [71] and consequently produces no CzcCBA complexes. None of the chromosomal or chromid-encoded paralogs of the RND protein CzcA were found in strain AE104 (Supplementary Table S2) and, with the exception of *zniA* and *zneA*, the respective genes were inactivated [7, 8, 82]. From the number of B-proteins, 22 ZniCBA complexes could be present in nonchallenged AE104 cells, so that Zni might have had some influence on the flow equilibrium of zinc under the condition that ZniA had also been produced in strain AE104. Unchallenged CH34 cells contained ZniA and even ZneA leading to 22 ZniCBA and 3 ZneCBA, in addition to the 24 CzcCBA complexes. However, the strong differences between zinc import into CH34 and AE104 cells during the uptake period of the pulse-chase experiment [25] indicated that the presence of CzcCBA had the strongest influence on zinc accumulation, especially when *czc* was upregulated under metal stress.

While the CzcCBA components could be quantified in the proteome of *C. metallidurans*, most of the other zinc transport systems were not, or at least not under all conditions (Tables 3 and 4). This was probably the result of the underrepresentation of membrane proteins in the proteomic analysis, as demonstrated by the subunits of the F_1_F_0_ ATPase (Supplementary Table S3), which was present in about ∼1500 copies per cell as judged from the α-subunit. The functions that describe the dependence of the protein abundance under the six conditions from the abundance of the sense and antisense transcripts (Supplementary Table. S6) allow a rough estimation of the numbers of these proteins (Fig. 4). These numbers were in the same range as the determined copy numbers, when these data were available. Nonchallenged CH34 cells, for instance, contained 394 ± 242 CorA_1_ (estimate 170), 391 CorA_2_ as single result (estimate 195), 162 ± 96 CorA_3_ (estimate 293), 29 ± 17 MgtA (estimate 71), and 82 ± 22 ZntB (estimate 73, Table 4 and Supplementary Table S6). These data demonstrated that a wide range of import systems [29–31, 83], mainly for the macro-bioelements phosphate and magnesium, did not change much in abundance under varying conditions. These systems are able to transport the ionic or phosphate-bound forms of various transition metal cations including Zn(II), leaving it to the subsequently acting efflux systems to adjust the cytoplasmic concentration and composition of these ions [2, 25].

The determined and estimated copy numbers of general metal import systems did not change much between metal-stressed and -starved *C. metallidurans* cells (Fig. 4), with the exception of a predicted two-fold upregulation of ZupT, which was in agreement with previous results [31, 83–85]. ZupT was responsible for 42% of the initial zinc import rate in nonchallenged cells (68 ^65^Zn cell^−1^ s^−1^) and 70% in zinc-starved cells (160 ^65^Zn cell^−1^ s^−1^) [25], which agrees with a duplication of the copy numbers.

In comparison to the zinc uptake system, the number of those able to export zinc ions across the inner membrane was strongly upregulated, from 350 general export systems for zinc (all estimated numbers) to 5330 (3425 ZntA measured, others estimated, Fig. 4). The numbers clearly emphasized the importance of efflux systems for metal homeostasis, which supports conclusions from biochemical, gene deletion and physiological studies [18, 25, 32, 33, 86, 87]. In total, the proteomic data close the knowledge gap between these studies and the investigation of the transcriptome of *C. metallidurans*.

### Role of the asRNAs as shown for the *zniBA* genes

**Figure 5.**
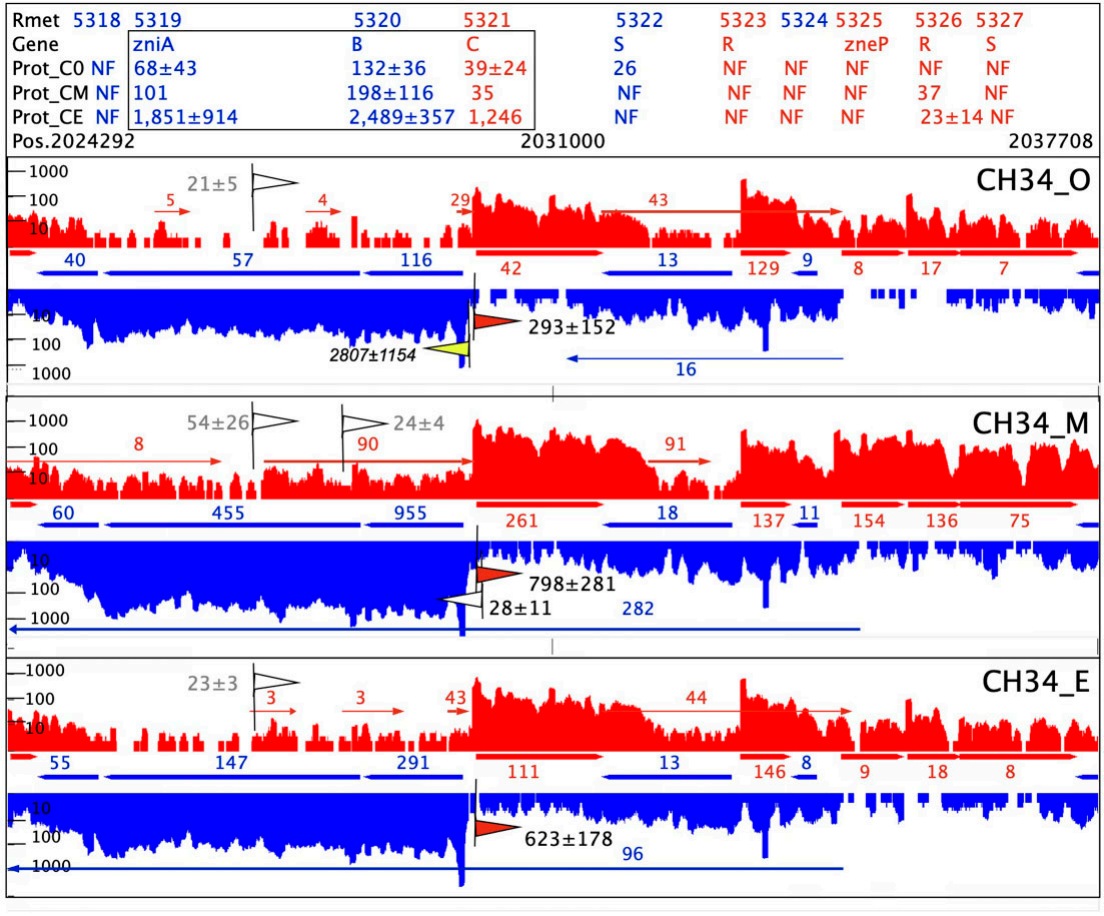
Dependence of the protein copy number on the transcriptional activities. The figure shows the RNASeq results of the chromid region ∼2 012 000 base pairs in metal-challenged (CH34_M), -starved (CH34_E), or control cells (CH34_0), transcripts of the forward strand in red, in reverse direction in blue. Arrows between the abundance plots show the position of the open reading frames and the NPKM values of the gene-specific sense RNA transcripts. Above or below the abundance plots are the position of the annotated asRNAs and their NPKM values. The header on the top gives the Rmet locus number, the gene name, the copy number of the gene product under the three conditions plus a position marker. Flags indicate transcriptional start sites (TSS), white not associated to a sigma factor yet, and red RpoD-dependent. The yellow flag indicates the published TSS *zniBp* [8], which was, however, no longer found in recent experiments. The TSS signal score is given adjacent to the flags, the no longer found *zniBp* in italics. The genes *zniB, zniC*, their 5′ untranslated regions and TSSs are strongly overlapping. Coordinates annotated to position 2 030 000 of the chromid sequence are from the left to the right −165 (*zniC-*5′UTR), −105 (3′ of *zniB)*, −77 (old *zniBp*), +10 (*zniCp*), +16 (previously published *zniC-*5′UTR), +59 (new found *zniBp*), +64 (5′ of *zniC*) and +109 (*zniB-*5′UTR). This indicates a lively interaction between transcription initiation of *zniC* and *zniB*.

In contrast to *czcCBA*, these *zni* genes are oriented as *zniBA* on one DNA strand and *zniC* on the other, both operons starting at the same promoter regions (Fig. 5). A strong transcriptional interference occurs at the divergent *zniB–zniC* region. The 5′ untranslated regions (5′UTRs) of both genes overlap, with each other and extend even into the opposing gene. The promoter *zniCp* depends on the main sigma factor RpoD, as found in a published and recently performed determination of the transcriptional start sites (TSS) in *C. metallidurans* [8]. In contrast, the additional RpoD-dependent and published *zniBp* [8] was no longer found in the recent measurement. Instead, the 5′UTR of *zniC* extended more deeply into *zniB* as in the previous measurements (Fig. 5). This is all in agreement with a heavy transcriptional interference in the *zniB–zniC* region, based on RNAP competition and asRNA effects by overlapping 5′UTRs [88, 89]. This interaction may be required to express *zniB* and *zniC* in such a way that the resulting subunit ratio is 2:1.

Moreover, the location of the genes of the *zni–zne* region switches between both DNA strands, resulting in an excludon effect [90]. Most intriguingly, and indicated by the position of ZniB and ZniA in Fig. 3, transcription of *zniBA* was upregulated eight-fold in metal-challenged *C. metallidurans* CH34 cells compared to the control, but this results only in a meager and not significant 50% increase in the copy number of the respective gene products (Fig. 5). Obviously, Zni might not be required in metal-stressed *C. metallidurans* CH34 cells with their active Czc system.

On the other hand, expression of *zniBA* was upregulated 2.5-fold in metal-starved CH34 cells but the protein copy number increased 10-fold. In control and metal-starved cells, a TSS in the middle of *zniA* resulted in one or two short asRNAs, respectively, of *zniBA*. These might represent RNAse III-mediated degradation products of double-stranded RNA [91]. But in metal-challenged cells with their comparably low protein synthesis yield despite an eight-fold upregulation of transcription, the activity of this promoter doubles, which leads to an asRNA with a much higher abundance that starts in the middle of *zniA* and continues into the already busy *zniB–zniC* divergent region. Half way, yet another TSS appears [58]. This asRNA seems to be responsible for the comparably low translation yield of ZniCBA. It seems as if during evolution of *C. metallidurans* and following acquisition of the *czc* determinant, *zni–zne* became subordinate. It turned from a zinc-exporting transenvelope system into one that only mediates zinc export under conditions when CzcCBA is not able to function, namely under metal-starvation conditions. An asRNA appears to have been central in mediating this subordination.

## Experimental

### Bacterial strains and growth conditions

*Cupriavidus metallidurans* strains used in this study were CH34 (wild type) and its plasmid-free derivative AE104, which lacks pMOL28 and pMOL30 [71]. Tris-buffered mineral salt medium [71] containing 2-g sodium gluconate/L (TMM) was used to cultivate these strains aerobically with shaking at 30°C. Analytical grade salts of cation chlorides, potassium chromate, and potassium arsenate were used to prepare 1 M stock solutions, which were sterilized by filtration. Tris-buffered media were solidified by incorporating 20-g agar/L.

To challenge *C. metallidurans* CH34 and AE104 simultaneously with several transition metal cations, a MultiTox metal mix optimized for the transcriptome determination [58] was employed. The CH34-specific MultiTox metal mix was 3.35 mM, composed of 461 µM Zn(II), 241 µM Cu(II), 1503 µM arsenate, 0.37 µM Hg(II), 19 µM chromate, 15 µM Cd(II), 761 µM Ni(II), and 347 µM Co(II). Strain AE104 was challenged with 1000 µM of its specific MultiTox mixture comprising 5 µM Zn(II), 120 µM Cu(II), 849 µM arsenate, 0.18 µM Hg(II), 7 µM chromate, 3 µM Cd(II), 9 µM Ni(II), and 7 µM Co(II). These MultiTox metal mixes should lead to a comparable toxicity of each metal in the cells of the strains CH34 and in AE104. To obtain general metal starvation conditions, CH34 was treated with 1576 µM EDTA and AE104 was treated with 306 µM EDTA, which were the respective IC_50_ values of EDTA for these strains.

### Quantitative proteome analysis

For the quantitative proteome analysis, the strains were cultivated to the mid-exponential phase, challenged at 100 Klett units with MultiTox or EDTA, harvested at 150 Klett units, and crude extract was prepared by ultra-sonication as published [92]. After low-touring centrifugation (30 min, 4500 × *g*), ultracentrifugation (1 h, 540 000 × *g*) was used to separate soluble and membrane proteins. Protein concentration was determined using the BCA Protein Assay Kit (Sigma-Aldrich, Germany). Protein digestion was performed using the SP3 protocol [93]. Briefly, 100-µg proteins of both fractions were incubated with Sera-Mag Speedbeads Carboxylate-Modified Magnetic Particles (1 µM GE Healthcare) for subsequent protein binding, washing, and in solution digestion. Protein binding on the magnetic beads was induced using 70% ACN (v/v, acetonitrile), followed by 20-min incubation at RT (room temperature) with shaking at 400 rpm in a ThermoMixer C (Eppendorf). Two additional washing steps with 70% EtOH were performed, followed by resuspension of the magnetic beads in 100% ACN, and complete lyophilization of the beads. Afterwards, the beads were resuspended in 50-µl 50 mM ammonium bicarbonate. The reduction reaction was carried out by using 10 mM DTT (Dithiothreitol) at 80°C while shaking for 15 min. The alkylation of the cysteine residues was done using 20 mM CAA (chloracetamide) at room temperature in the dark. The supernatant was discarded and 50-µl 50 mM ammonium bicarbonate was added to resuspend the magnetic beads. Protein digestion was carried out with 2-µg trypsin/Lys-C Mix (Promega, USA) overnight at 37°C; 10-µg peptide solution was further purified using the Pierce C18 spin tips (Thermo Fisher Scientific, USA). The peptide solution was adjusted to a pH below 4 using 2.5% trifluoroacetic acid and scaled up to 20 µl using 0.1% trifluoroacetic acid. Prior use, the tips were first wetted using 20-µl 0.1% trifluoroacetic acid in 80% ACN, and then equilibrated only with 0.1% trifluoroacetic acid, three times. After each step, the tips were centrifuged for 1 min at 1000 × *g*; 20 µl of the peptide solution was applied to the C18 spin tips followed by centrifugation, and the same centrifugation conditions were applied throughout the purification procedure. Washing of the bound peptides was done using 20-µl 0.1% trifluoroacetic acid twice, and elution was achieved with 20-µl 0.1% trifluoroacetic acid in 80% ACN, performed twice to elute all bound peptides. Last, the peptides were dried with a SpeedVac vacuum concentrator. Peptides were resuspended in 25-µl 0.1% fluoroacetic acid and sonicated in a water bath for 15 min. Nano-ESI-LC-MS-MS (nano electrospray ionisation liquid chromatography tandem mass spectrometry) was performed downstream for whole proteome analysis.>

### Liquid chromatography-coupled mass spectrometry

Approximately 200 ng of peptides for each sample and replicate were initially trapped (PepMap100 5 µm, 3 × 5 mm Thermo Scientific #160454) and separated on a Waters M-Class C18 25-cm analytical column (Acquity UPLC® M-Class Peptide BEH 130 Å, 1.7 µm, 75 µm × 250 mm, Waters #186007484) over 180 min with an increasing gradient of ACN (3%–22%) at 240 nl/min on a Dionex UltiMate 3000 RSCLnano System before being injected in to a Thermo Scientific Orbitrap Exploris 480 mass spectrometer. Peptides were ionized in positive mode with 1800 V and a transfer capillary temperature of 300°C. Samples were subjected to further separation with FAIMS Pro with three compensation voltages (CVs: −40, −55, and −65) resulting in three separate MS experiments within one data file. Each experiment had the following settings: MS resolution of 120 000 at 200 m/z, a scan range of 350–1400 m/z, MS AGC (automatic gain control) target of 300% for max IT (injection time) of 50 ms; MSMS (tandem mass spectrometry) of all the most intense peaks for a total cycle time of 1 s with the following settings: isolation window of 2-m/z normalized collision energy of 30%, resolution of 15 000 with an AGC target of 100%, and a max 30-ms IT. Every fragmented precursor within ±10 ppm was immediately excluded from reanalysis for 45 s.

### Data analysis

Raw files were analyzed by MaxQuant software [94] version 2.0.3.0 and searched against the *C. metallidurans* Uniprost FASTA database (UniProt ID: UP000002429). Generally, the already set parameters were kept. *N*-terminal acetylation and methionine oxidation were added as variable modification. False discovery rate was set to 0.01 for peptides (minimum length of seven amino acids). For protein identification, unique and razor peptides were set to 1. Group-specific parameters for the instrument were set to Orbitrap and 2-ppm peptide search tolerance. Match between runs was enabled and protein quantification was set to LFQ (label-free quantification), with classic normalization type.

### Statistical analysis, reference protein mixtures, and data normalization

The accumulated LFQ intensity for the identified protein in each of the three technical repeats was used to calculate the mean values and deviations of the protein amounts per biological repeat and bacterial strain. These values were not normalized to 1 million proteins per bacterial cell but instead to 1.88 million proteins per cell. This number was calculated from the number of bacterial cells before harvest and the protein concentration in the crude extract, which gave an average protein content of 115 fg/cell. This number was divided by the average protein mass of 37.2 kDa of the synthesized proteome, ∑{(fmol(protein_i_)/∑fmol(allproteins)^.^mass(protein_i_)}, yielding 3.088 amol protein per cell, which was multiplied with the Avogadro number. The resulting 1.86 million proteins per cell were lower than the number of 2.6 million proteins per *E. coli* cell, although *E. coli* has a 1.5-fold lower cell volume and should have a lower dry mass [86, 95–97].

The resulting cellular numbers and deviations for each protein per biological repeat and bacterial strain were used to calculate the mean protein numbers per bacterial cell and strain. The deviation was the mean value of the deviations of the biological repeats. Finally, the protein numbers of the strains were compared. A protein number was judged as up- or downregulated if the quantities were at least two-fold lower or higher in the mutant-derived extract, respectively. Additionally, a ‘D’ value was calculated as a measure of the significance of the difference. The ‘D’ value gives the difference in the protein numbers, mutant minus parent, divided by the sum of the deviation of these mean values. If D >1, the deviation bars of the mean values do not overlap, leading to a significant result (>95%) if *n* >3 as published [98]. If a protein was up- or downregulated and the difference between the protein numbers was significant (D ≥1), the value was judged as significantly different. All values are provided in the Supplementary Data Set.

To verify compatibility and reproducibility of the method used for the determination of the proteome, the protein content of unchallenged cells of *C. metallidurans* strain AE104 was plotted against the already published data [61] (Supplementary Fig. S1). The data points were on a line with a regression coefficient of 100% and a slope of 1.0000 ± 3.16×10^−16^. A number of 2816 proteins were found in both data sets.

For the six data sets, the mean values and deviations of the copy numbers were calculated if a protein was quantified in two or three of the samples of one data set. Proteins found only once in a data set were not excluded but noted with a ‘0’ as deviation, which served as a flag for such a result. From the six data sets, the ratios Q of the copy numbers for each protein in the comparisons CH34 cells with compared to without toxic metals (CM0), with or without EDTA (CME), and the same for AE104 cells (AM0, AME) plus the comparison AE104 with CH34 (0AC) and the distance value D (= absolute[value1 − value2]/[deviation1 + deviation2]) as a measure of significance, were calculated. Comparisons that included only one result in a data point give a D value of 0 or a calculation error, indicating that a respective comparison was not a significant result. Proteins not found were annotated with a copy number of 1 per cell to allow subsequent mathematical operations, for instance calculation of the ratio Q of the copy numbers of two conditions. The full data set is provided as the Supplementary Data Set.

## Supplementary Material

mfae058_Supplementary_Files

## Data Availability

The mass spectrometry proteomics data have been deposited to the ProteomeXchange Consortium via the PRIDE (www.ebi.ac.uk/pride) partner repository with the data set identifier PXD057750.
