# Supplementary material for: Linking the transcriptome to physiology: response of the proteome of Cupriavidus metallidurans to changing metal availability
Source: Metallomics. 2024 Nov 19;16(12):mfae058. doi: 10.1093/mtomcs/mfae058 (PMC11647595; doi:10.1093/mtomcs/mfae058)
Supplement: mfae058_Supplementary_Files [file mfae058_supplementary_files.zip › Suppl_data_Supplementary_Material.pdf]

Supplementary Material

Supplementary information. Constraints and limitations of the proteomic approach used. When the copy numbers of the new measurement of unchallenged strain AE104 cells were compared with the published values, the double-log plot (Suppl. Fig. S1) yielded a linear function with a slope of 1.0000 ± 3.16*10^-16 and regression coefficient of 100%. The proteome determination was reproducible and comparable to the published value.

The top-ten of the proteins with the highest copy numbers ranged between 18,000 and 66,500 copies per cell (Suppl. Table S1). These were periplasmic binding proteins, ribosomal proteins and proteins associated with translation, chaperones, a component of the RNA polymerase, of the TCA cycle, the outer membrane, and the iron-containing superoxide dismutase SodB in one example. The deviation of the measurements of the three determinations per condition was between 4% and 121% with a mean value of 40%, which was similar to the median value. As a consequence of this result, only ratios larger than two between the copy number of two conditions were taken into consideration, in addition to a distance value > 1 (D is the absolute difference of the mean values divided by the sum of the deviations. At n ≥ 3, D > 1 means > 95% confidence).

The ribosomal proteins were used to identify the size bias of the proteome determination (Suppl. Table S2). The protein with the highest copy number among the ribosomal components was RplD with between 22,000 and 28,000 copies per cell in *C. metallidurans* CH34 and 47,000 copies per cell in metal-challenged AE104 cells, and RpsA with 36,000 or 40,000 copies per cell in non-challenged and metal-starved AE104 cells. Assuming each ribosomal protein appears in one copy per ribosome, the mean copy numbers of the ribosomal proteins in the six conditions were normalized to RplD (Suppl. Fig. S2). Proteins with sizes up to 100 aa were found with an efficiency of only 20%. Large proteins yielded better efficiencies than smaller ones, probably because they created more specific trypsin fragments than small proteins.

Another bias concerned membrane-bound proteins. This was demonstrated with the components of the F_1F_0 ATPase (Suppl. Table S3). The subunit composition of the complex is $Atp(A_3D_3CGH\text{-}BF_2E_{10})$ [1]. Ten c subunits per c rotor were assumed here but this number has not been determined for *C. metallidurans*. With respect to the number of components per complex, the gamma subunit AtpG yielded the highest copy numbers (Table 2). Since no difference between the copy numbers in the various conditions could be observed, the mean value of the gamma subunits per cell of 2,574 ± 788 approximated the number of F_1F_0 complexes per *C. metallidurans* cell. There was also no difference between the copy numbers for the other subunits under the six conditions, although these numbers were below the 3-fold (AtpA, AtpD), two-fold (AtpF) and 10-fold (AtpE) numbers of AtpG. For the F1 components and the b subunit of the F0 complex, these numbers were between 49% and 79% of the

determination yield of the gamma subunit, when the respective portion of a subunit of the F1F0 complex was included. The exception here was the delta subunit AtpH, which was strongly under-represented (6% of AtpG). The remaining F_0 subunits a and c were measured with a yield of 17% and 11%, respectively. This indicated that membrane-bound proteins without extensions into the periplasm or cytoplasm were under-determined or under-represented with a yield of only about 14%.

Supplementary Table S1. Proteins with the highest copy numbers in *C. metallidurans* cells[a].

CH34_0	CH34_M	CH34_E	AE104_0	AE104_M	AE104_E
Bug; 55,923±38,451	Bug; 51,599±62,391	Bug; 66,449±30,906	RpsA; 40,263±6,777	RplD; 46,686±28516	Bug; 50,627±20,733
Tuf; 29,841±14,614	Bug2; 47,603±31,526	OmpA; 35,597±9,999	RpoA; 39,210±8,089	RpsA; 41,184±22486	RpsA; 35,539±18,079
RplD; 28,245±2,178	OmpA; 46,396±29,065	RpoA; 33,710±5,802	Bug; 37,918±22,955	Bug; 35,957±23417	Tuf; 34,057±9,028
OmpA; 26,166±5,649	LivK1; 40,331±11,022	LivK1; 30,238±8,039	Tuf; 30,728±7,035	LivK1; 34,320±12268	RpoA; 32,079±5,609
LivK1; 24,099±7,757	GroL; 33,489±16,622	Bug2; 29,691	RplD; 30,713±12,541	RplI; 29,012±20295	GroL; 29,410±12,391
GroL; 23,744±1,979	GltI; 32,548±15,369	GltI; 27,496±9,338	LivK1; 27,394±10,203	Tuf; 28,871±6391	Pnp; 29,153±25,385
RplF; 21,869±4,167	RpoA; 31,130±7,883	Tuf; 26,946±10,195	Rm4742; 25,075±3,683	RplS; 27,768±16178	RplD; 26,245±15,047
RpsA; 20,078±8,677	Tuf; 26,177±8,758	GroL; 26,256±3,506	RplA; 23,675±11,593	RpsD; 27,163±16455	LivK1; 25,814±7,524
RplP; 18,477±1,983	PstS; 25,695±2,896	Mdh; 21,646±9,878	RpsD; 22,950±7,242	RpsN; 26,478±15530	Bug2; 23,693±13,870
RpsG; 18,279±736	RplD; 23,173±10,602	RpsN; 21,576	SodB; 22,008±1,285	GltI; 26,276±7912	RpsN; 21,280±10,558

[a]The top-ten of proteins yielding the highest copy numbers in *C. metallidurans* wild type CH34 under non-challenging conditions (CH34_0), under metal stress (CH34_M) and EDTA-generated metal starvation stress (CH34_E), and of the plasmid-free derivative strain AE104 under comparable conditions are listed. The colors of the cells of the table are just for easier comparisons within the rows. Bug, Bug2, PstS and LivK1 are periplasmic binding proteins, Tuf is the elongation factor Tu, GroEL a chaperone, Rpl and Rps are ribosomal components, Mdh is the malate dehydrogenase of the TCA cycle, RpoA the alpha-subunit of the RNA polymerase, SodB the iron-containing superoxide dismutase and OmpA an outer membrane protein. The mean value plus deviation is given, n = 3. If found only once, no deviation is given.

Supplementary Table S2. Abundance of selected gene products in *C. metallidurans* CH34 wild type and its plasmid-free derivative strain AE104 under metal-shocked, -starved and control conditions.
The locus tag is shown, the gene name and the abundances with deviations with "±0" indicating a single appearance among the triplicate determination. NF, not found.
The abundances are followed by (Q; D) indicating the ratio Q and the distance values of the comparison metal-shocked and -starved cells to the control, plus AE104 to CH34 control cells.

locus tag	gene	CH34_0	CH34_M	CH34_E	AE104_0	AE104_M	AE104_E	Description
**czc region: not found *flgB, ompP, czcJ, czcD, czcI, czcN, czcM*.**								
Rmet_5970	*czcP*	31±19	76; (2.4; 2.4)	NF				Q1LAJ7 Heavy metal translocating P-type ATPase
Rmet_5976	*czcE*	NF	96; (96.2; 95.2)	NF				Q1LAJ1 Putative uncharacterized protein
Rmet_5977	*czcS*	NF	313±270; (313.1; 1.2)	NF				Q44007 Sensor protein czcS
Rmet_5978	*czcR*	67	566±539; (8.5; 0.9)	89±52; (1.3; 0.4)				Q44006 Transcriptional activator protein czcR
Rmet_5980	*czcA*	95±40	854±489; (9.0; 1.4)	75±50; (0.8; 0.2)				P13511 Cobalt-zinc-cadmium resistance protein czcA
Rmet_5981	*czcB*	146±104	1509±607; (10.4; 1.9)	113±26; (0.8; 0.2)				P13510 Cobalt-zinc-cadmium resistance protein czcB
Rmet_5982	*czcC*	17	391±207; (22.7; 1.8)	NF				P13509 Cobalt-zinc-cadmium resistance protein czcC
**czc2 regions: not found *czcB2'; czcR2, czcS2, czcA2, czcB2''*.**								
Rmet_4594	*zntA*	NF	3425±2300; (3424.7; 1.5)	NF	502; (502.3; 501.3)		NF	Q1LEH0 Heavy metal translocating P-type ATPase
Rmet_4595	*czcI2*	NF	NF	NF	NF	NF	NF	Q1LEG9 Putative uncharacterized protein
Rmet_4596	*czcC2*	NF	82; (81.8; 80.8)	NF	NF	892±583; (891.8; 1.5)	NF	Q1LEG8 Outer membrane efflux protein
**cnr: not found *cnrY, cnrC, cnrA*.**								
Rmet_6206	*cnrX*	44	388±176; (8.8; 2.0)	NF				P37975 Nickel and cobalt resistance protein cnrR
Rmet_6207	*cnrH*	67		NF				P37978 RNA polymerase sigma factor cnrH
Rmet_6209	*cnrB*	207	1235±272; (6.0; 3.8)	NF				P37973 Nickel and cobalt resistance protein cnrB
Rmet_6211	*cnrT*	11	NF	NF				Q9L3G0 CnrT protein
**zni/zne region: not found *Rmet_5318, zniR, Rmet_5324, zneP, zneS*.**								
Rmet_5319	*zniA*	68±43	101; (1.5; 0.8)	1851±914; (27.1; 1.9)	NF	242; (241.6; 240.6)	181±117; (181.2; 1.5)	Q1LCE8 Heavy metal efflux pump CzcA
Rmet_5320	*zniB*	132±36	198±116; (1.5; 0.4)	2480±357; (18.8; 6.0)	131±76; (1.0; 0.0)	207±96; (1.6; 0.4)	454±247; (3.5; 1.0)	Q1LCE7 Secretion protein HlyD
Rmet_5321	*zniC*	39±24	35; (0.9; 0.2)	1246; (32.0; 51.1)	44±8; (1.1; 0.2)	55±33; (1.2; 0.3)	147±75; (3.3; 1.2)	Q1LCE6 RND efflux system, outer membrane lipoprotein, NodT
Rmet_5322	*zniS*	26	NF	NF	NF	NF	22; (21.5; 20.5)	Q1LCE5 Sensor protein
Rmet_5326	*zneR*	NF	37; (36.7; 35.7)	23±14; (22.7; 1.5)	14; (13.6; 12.6)	19±16; (1.4; 0.3)	NF	Q1LCE1 Two component transcriptional regulator, winged helix family
Rmet_5328	*zneC*	NF	NF	87±16; (86.7; 5.1)	NF	7; (7.4; 6.4)	NF	Q1LCD9 RND efflux system, outer membrane lipoprotein, NodT
Rmet_5329	*zneA*	14	NF	318±188; (23.0; 1.6)	NF	NF	NF	Q1LCD8 Heavy metal efflux pump ZneA
Rmet_5330	*zneB*	16	NF	464±52; (28.8; 8.5)	NF	NF	19; (18.6; 17.6)	Q1LCD7 Secretion protein ZneB
Rmet_5331	*zneR2*	28±16	64; (2.3; 2.3)	67; (2.4; 2.5)	39±3; (1.4; 0.6)	34; (0.9; 1.6)	43±27; (1.1; 0.2)	Q1LCD6 Two component transcriptional regulator, winged helix family
Rmet_5332	*zneS2*	324±195	202; (0.6; 0.6)	110; (0.3; 1.1)	NF	NF	165; (165.4; 164.4)	Q1LCD5 Sensor protein
**ncc: no proteins found**								
**hmv: not found hmvA**								
Rmet_3836	*hmvC*	170±137	92±56; (0.5; 0.4)	213±134; (1.3; 0.2)	113±59; (0.7; 0.3)	86; (0.8; 0.5)	71±33; (0.6; 0.5)	Q1LGM0 Outer membrane efflux protein
Rmet_3837	*hmvB*	372±243	381±223; (1.0; 0.0)	952±194; (2.6; 1.3)	541±111; (1.5; 0.5)	370±368; (0.7; 0.4)	398±17; (0.7; 1.1)	Q1LGL9 Secretion protein HlyD
**hmy: no hmy proteins found**								
**nim: not found *nimA1, nimA2*.**								
Rmet_5677	*nimC*	59	40±24; (0.7; 0.8)	NF	84±57; (1.4; 0.4)	60±41; (0.7; 0.2)	NF	Q1LBE0 Putative uncharacterized protein
Rmet_5682	*nimB*	873±214	1203±193; (1.4; 0.8)	283±103; (0.3; 1.9)	1412±436; (1.6; 0.8)	1320±325; (0.9; 0.1)	1034±697; (0.7; 0.3)	Q1LBD5 Secretion protein HlyD
**hmz: not found *hmzA, hmzB, yodB, hmzS*.**								
Rmet_3014		75	NF	NF	NF	NF	NF	A0HB61 Putative uncharacterized protein
Rmet_3016	*hmzR*	11±7	117; (10.3; 16.1)	23±5; (2.0; 0.9)	NF	NF	NF	Q1LIY7 Two component transcriptional regulator, winged helix family
**cus: not found *cusD, cusA*.**								
Rmet_5031	*cusC*	NF	NF	NF	NF	193±71; (192.9; 2.7)	NF	Q1LD83 Putative outer membrane cation efflux protein
Rmet_5032	*cusB*	NF	NF	NF	NF	156±80; (156.0; 1.9)	NF	Q1LD82 Secretion protein HlyD
Rmet_5034	*cusF*	NF	NF	NF	NF	203±147; (203.2; 1.4)	NF	Q1LD80 Conserved hypothetical signal peptide protein
**sil: no products found**								
**cusF2: not found**								
**cad: not found *Rmet_2299, 2300, 2301, cadA, cadC.***								
Rmet_2302	*cadR*	62±36	38; (0.6; 0.7)	122±53; (2.0; 0.7)	72±48; (1.2; 0.1)	75±53; (1.0; 0.0)	99; (1.4; 0.6)	A7HYK9 Putative transcriptional regulator, MerR family
**pbr: not found *pbrU, pbrR, pbrA, pbrB/C, pbrD*.**								
Rmet_5945	*pbrT*	206±156	223±47; (1.1; 0.1)	847±353; (4.1; 1.3)	NF	NF	NF	Q58AJ4 PbrT protein (Iron permease FTR1)
**cop: not found *copV, copT, copM, copK, copD1, copJ, copG, copL, copQ, copE, copW*.**								
Rmet_6109	*copN*	NF	112±25; (111.6; 4.2)	NF				Q1LA58 Putative uncharacterized protein
Rmet_6110	*copS1*	NF	467±471; (467.0; 1.0)	NF				Q58AD4 Sensor protein
Rmet_6111	*copR1*	21±9	322±125; (15.6; 2.2)	22±8; (1.0; 0.1)				Q58AD5 Two component response transcription regulator
Rmet_6112	*copA1*	NF	1054±623; (1054.4; 1.7)	NF				Q58AD6 Copper resistance transmembrane protein (Copper-resistance protein CopA)
Rmet_6113	*copB1*	NF	1559±870; (1559.4; 1.8)	NF				Q58AD7 CopB protein (Copper resistance B)
Rmet_6114	*copC1*	NF	704±120; (704.4; 5.8)	NF				Q1LA53 Copper resistance protein CopC
Rmet_6116	*copI*	NF	2274±982; (2273.7; 2.3)	NF				Q58AE0 Putative oxydoreductase (Blue (Type 1) copper domain)
Rmet_6119	*copF*	195±116	768±701; (3.9; 0.7)	46±21; (0.2; 1.1)				Q58AE3 Cation-transporting p-atpase (Heavy metal translocating P-type ATPase)
Rmet_6122	*copH*	NF	5301±2837; (5301.1; 1.9)	36; (36.3; 35.3)				Q58AE5 CopH protein (Putative uncharacterized protein)

**cup: not found: *ompW1*.**								
Rmet_3521	*betA2*	707±364	434±279; (0.6; 0.4)	513±132; (0.7; 0.4)	353±152; (0.5; 0.7)	206±73; (0.6; 0.7)	420±187; (1.2; 0.2)	Q1LHI3 Glucose-methanol-choline oxidoreductase
Rmet_3522	*pldB*	503±214	599±166; (1.2; 0.3)	522±202; (1.0; 0.0)	428±76; (0.9; 0.3)	560±261; (1.3; 0.4)	474±239; (1.1; 0.1)	Q1LHI2 Alpha/beta hydrolase fold
Rmet_3523	*cupR*	38	529±114; (13.8; 4.3)		25±15; (0.7; 0.9)	164±145; (6.5; 0.9)	36±22; (1.4; 0.3)	Q1LHI1 Transcriptional regulator, MerR family
Rmet_3524	*cupA*	309±184	2640±1113; (8.6; 1.8)	142; (0.5; 0.9)	185±103; (0.6; 0.4)	858±775; (4.6; 0.8)	182±114; (1.0; 0.0)	Q1LHI0 Heavy metal translocating P-type ATPase
Rmet_3525	*cupC*	77±20	457±265; (5.9; 1.3)	185±104; (2.4; 0.9)	55±24; (0.7; 0.5)	556±325; (10.2; 1.4)	107±65; (2.0; 0.6)	Q1LHH9 Heavy metal transport/detoxification protein
**cop2: not found: *copD2, cobB2*.**								
Rmet_5669	*copC2*	NF	37; (37.3; 36.3)	NF	10; (10.3; 9.3)	612±693; (59.4; 0.9)	NF	Q1LBE8 Copper resistance protein CopC
Rmet_5671	*copA2*	14±8	1053±1047; (74.7; 1.0)	19; (1.4; 0.6)	NF	1321±399; (1320.9; 3.3)	NF	Q1LBE6 Copper-resistance protein CopA
Rmet_5672	*copR2*	27±22	157±132; (5.7; 0.8)	31; (1.1; 0.2)	34±13; (1.3; 0.2)	412±238; (12.0; 1.5)	35±10; (1.0; 0.1)	Q1LBE5 Two component heavy metal response transcriptional regulator, winged helix family
Rmet_5673	*copS2*	39±26	287±218; (7.3; 1.0)	NF	NF	56; (55.5; 54.5)	13; (12.9; 11.9)	Q1LBE4 Sensor protein
**chr: not found *chrZ, chrP, chrF1, chrA1, chrI*.**								
Rmet_6195	*chrY*	NF	263±152; (262.7; 1.7)	NF				Q1L9X2 Putative uncharacterized protein
Rmet_6197	*chrN*	NF	129±77; (128.5; 1.6)	NF				Q1L9X0 Putative uncharacterized protein
Rmet_6198	*chrO*	NF	727±239; (727.4; 3.0)	17; (16.7; 15.7)				Q1L9W9 Putative uncharacterized protein
Rmet_6200	*chrE*	NF	314±199; (314.3; 1.6)	NF				Q5NUZ8 Superoxide dismutase SodM-like protein (Rhodanese-like protein)
Rmet_6201	*chrC*	104	1318±1154; (12.7; 1.1)	NF				P17550 Superoxide dismutase [Fe]
Rmet_6203	*chrB1*	NF	649±516; (648.9; 1.3)	NF				P17552 Protein chrB
**chr2: not found *chrF2, chrA2*.**								
Rmet_3866	*chrB2*	NF	307±47; (306.6; 6.3)	NF	NF	218±63; (217.9; 3.4)	NF	Q1LGJ2 Putative chromate resistance signal peptide protein
Rmet_3867		NF	44; (44.3; 43.3)	NF	35; (35.1; 34.1)	NF	NF	Q1LGJ1 Transcriptional regulator, AraC family
**ars: not found *arsP, arsB, arsM, Rmet_0335*.**								
Rmet_0328	*arsH*	NF	720±182; (720.0; 3.9)	NF	NF	699±324; (698.8; 2.1)	NF	Q1LRL2 NADPH-dependent FMN reductase
Rmet_0329	*arsC1*	NF	514±330; (513.9; 1.6)	NF	NF	424±137; (424.0; 3.1)	NF	Q1LRL1 Arsenate reductase
Rmet_0331	*arsC2*	34	1538±686; (45.2; 2.2)	38±22; (1.1; 0.2)	54±37; (1.6; 0.5)	2605±1802; (48.7; 1.4)	41±25; (0.8; 0.2)	Q1LRK9 Protein tyrosine phosphatase
Rmet_0332	*arsI*	NF	220±121; (220.4; 1.8)	NF	NF	410±380; (409.5; 1.1)	NF	Q1LRK8 Glyoxalase/bleomycin resistance protein/dioxygenase
Rmet_0333	*arsR*	22±13	1126±371; (51.0; 2.9)	26±1; (1.2; 0.3)	28±3; (1.3; 0.4)	578±315; (20.6; 1.7)	33±11; (1.2; 0.4)	Q1LRK7 Transcriptional regulator, ArsR family
Rmet_0336		104±72	119±59; (1.1; 0.1)	140±81; (1.3; 0.2)	99±43; (1.0; 0.0)	140±118; (1.4; 0.3)	150±88; (1.5; 0.4)	Q1LRK4 Transcriptional regulator, IclR family
Rmet_0337		95±57	151; (1.6; 1.0)	97; (1.0; 0.0)	178±94; (1.9; 0.6)	115±75; (0.6; 0.4)	172±114; (1.0; 0.0)	Q1LRK3 Transcriptional regulator, IclR family
**chromosomal mer: not found *merT*.**								
Rmet_2312	*merR*	18	NF	NF	106±84; (5.8; 1.0)	56±34; (0.5; 0.4)	90±52; (0.8; 0.1)	Q8GQ23 Organomercurial resistance regulatory protein MerR
Rmet_2314	*merP*	NF	513±149; (513.3; 3.4)	NF	NF	476±482; (476.1; 1.0)	NF	Q8GQ25 Periplasmic mercuric ion binding protein MerP
Rmet_2315	*merA''*	NF	172±54; (172.5; 3.1)	NF	NF	161±95; (160.8; 1.7)	NF	Q8GQ26 Mercuric (Hg(II)) reductase
**pMOL28 mer: not found *merR, merT, merD, merE, urf_2*.**								
Rmet_6346	*merP*	NF	3935±1757; (3934.8; 2.2)	48; (48.2; 47.2)				NA
Rmet_6183	*merA*	NF	11249±2250; (11248.6; 5.0)	NF				A6UXG5 Mercuric reductase
**pMOL30 mer1: no products found**								
**pMOL30 mer2: not found *merT, merD, merE, urf-2*.**								
Rmet_6171	*merR*	117±68	151±102; (1.3; 0.2)	146±38; (1.3; 0.3)				P69413 Mercuric resistance operon regulatory protein
Rmet_6173	*merP*	NF	3935±1757; (3934.8; 2.2)	48; (48.2; 47.2)				Q58AI1 Periplasmic mercuric-ion binding protein
Rmet_6174	*merA*	NF	249±147; (249.3; 1.7)	NF				Q1L9Z3 Mercuric reductase MerA
**gsh/bfr: not found *bfd*.**								
Rmet_0242	*gshA*	2190±780	1262±595; (0.6; 0.7)	979±670; (0.4; 0.8)	3597±470; (1.6; 1.1)	2887±1949; (0.8; 0.3)	3059±963; (0.9; 0.4)	Q1LRU8 Glutamate--cysteine ligase GshA
Rmet_0243	*gshB*	686±422	1086±223; (1.6; 0.6)	794; (1.2; 0.3)	854±392; (1.2; 0.2)	1014±607; (1.2; 0.2)	965±484; (1.1; 0.1)	Q1LRU7 Glutathione synthase
Rmet_0244		94±61	118±21; (1.3; 0.3)	132±20; (1.4; 0.5)	101±15; (1.1; 0.1)	150±101; (1.5; 0.4)	121±18; (1.2; 0.6)	Q1LRU6 PTS system fructose subfamily IIA component
Rmet_0245	*ptsH*	NF	548±221; (548.0; 2.5)	NF	255; (254.7; 253.7)	304±189; (1.2; 0.3)		Q1LRU5 HPrNtr
Rmet_0246	*ptsI*	417±186	456±439; (1.1; 0.1)	203±60; (0.5; 0.9)	663±431; (1.6; 0.4)	347±213; (0.5; 0.5)	347±40; (0.5; 0.7)	Q1LRU4 Phosphoenolpyruvate--protein phosphotransferase
Rmet_0248	*bfr*	14097±5106	14963±10787; (1.1; 0.1)	7327±2008; (0.5; 1.0)	13006±5947; (0.9; 0.1)	18664±4609; (1.4; 0.5)	9049±2206; (0.7; 0.5)	Q1LRU2 Bacterioferritin
**isc**								
Rmet_1024	*iscR*	177±98	950±226; (5.4; 2.4)	207±95; (1.2; 0.2)	211±67; (1.2; 0.2)	907±510; (4.3; 1.2)	137±49; (0.6; 0.6)	Q1LPL6 Transcriptional regulator, BadM/Rrf2 family
Rmet_1025	*iscS*	3030±1106	2704±663; (0.9; 0.2)	1211±227; (0.4; 1.4)	2424±1625; (0.8; 0.2)	3396±1591; (1.4; 0.3)	1496±420; (0.6; 0.5)	Q1LPL5 Cysteine desulfurase IscS
Rmet_1026	*iscU*	725±66	1724±499; (2.4; 1.8)	530±41; (0.7; 1.8)	535±202; (0.7; 0.7)	1577±718; (2.9; 1.1)	416±133; (0.8; 0.4)	Q1LPL4 FeS cluster assembly scaffold IscU
Rmet_1027	*iscA*	139±80	563±325; (4.1; 1.0)	70; (0.5; 0.9)	170±40; (1.2; 0.3)	492±286; (2.9; 1.0)	123±72; (0.7; 0.4)	Q1LPL3 Iron-sulfur cluster assembly protein IscA
Rmet_1028	*hscB*	62±50	183; (3.0; 2.4)	81±17; (1.3; 0.3)	41±24; (0.7; 0.3)	103±62; (2.5; 0.7)	59±45; (1.4; 0.3)	Q1LPL2 Co-chaperone Hsc20
Rmet_1029	*hscA*	490±97	611±63; (1.2; 0.8)	310±76; (0.6; 1.0)	592±183; (1.2; 0.4)	856±433; (1.4; 0.4)	333±171; (0.6; 0.7)	Q1LPL1 Chaperone protein hscA homolog
Rmet_1030	*fdx*	265±303	181±89; (0.7; 0.2)	252±128; (0.9; 0.0)	106±76; (0.4; 0.4)	232±212; (2.2; 0.4)	87±49; (0.8; 0.2)	Q1LPL0 Ferredoxin, 2Fe-2S type
Rmet_1031		NF	571±129; (571.2; 4.4)	373; (373.0; 372.0)	224±222; (224.4; 1.0)	487; (2.2; 1.2)	453; (2.0; 1.0)	Q1LPK9 Putative uncharacterized protein
**agr: not found *agrC, agrB, agrA, agrR, Rmet_1753*.**								
Rmet_1752	*agrS*	NF	26; (25.6; 24.6)	NF	28; (27.7; 26.7)	NF	NF	Q1LMJ5 Sensor protein
**pp/pst: not found *phoR, pstA*.**								
Rmet_2177	*ppx*	411	297±322; (0.7; 0.4)	515±232; (1.3; 0.4)	496±288; (1.2; 0.3)	472±333; (1.0; 0.0)	NF	Q1LLC0 Ppx/GppA phosphatase
Rmet_2178	*ppk*	168±75	257±203; (1.5; 0.3)	195±61; (1.2; 0.2)	167±97; (1.0; 0.0)	118±51; (0.7; 0.3)	183±105; (1.1; 0.1)	Q1LLB9 Polyphosphate kinase
Rmet_2180	*phoB*	120±38	441±107; (3.7; 2.2)	113±45; (0.9; 0.1)	230±107; (1.9; 0.8)	153±109; (0.7; 0.4)	119±50; (0.5; 0.7)	Q1LLB7 Two component transcriptional regulator, winged helix family
Rmet_2181	*phoU*	118±70	477±341; (4.0; 0.9)	134±28; (1.1; 0.2)	150; (1.3; 0.5)	96±57; (0.6; 0.9)	136±94; (0.9; 0.1)	Q1LLB6 Phosphate uptake regulator, PhoU

Rmet_2182	*pstB*	179±109	448±291; (2.5; 0.7)	98±56; (0.5; 0.5)	121; (0.7; 0.5)		108±37; (0.9; 0.3)	Q1LLB5 Phosphate import ATP-binding protein pstB
Rmet_2184	*pstC*	NF	68; (67.7; 66.7)	NF	NF	NF	NF	Q1LLB3 Phosphate ABC transporter, permease protein PstC
Rmet_2185	*pstS*	4474±2306	25695±2896; (5.7; 4.1)	2315±515; (0.5; 0.8)	4291±1346; (1.0; 0.1)	5647±1431; (1.3; 0.5)	5456±1936; (1.3; 0.4)	Q1LLB2 Periplasmic phosphate binding protein
Rmet_2186	*glmM*	651±39	451±20; (0.7; 3.4)	1113±564; (1.7; 0.8)	325±106; (0.5; 2.2)	720±680; (2.2; 0.5)	294±163; (0.9; 0.1)	Q1LLB1 Phosphoglucosamine mutase
Rmet_2187	*folP*	NF	NF	NF	207; (207.4; 206.4)	NF	NF	Q1LLB0 Dihydropteroate synthase
Rmet_2188	*ftsH*	3439±1146	1586±168; (0.5; 1.4)	2744±2094; (0.8; 0.2)	1982±927; (0.6; 0.7)	1156±825; (0.6; 0.5)	1502±1135; (0.8; 0.2)	Q1LLA9 ATP-dependent metalloprotease FtsH
Rmet_2189	*rrmJ*	308±193	361; (1.2; 0.3)	284±166; (0.9; 0.1)	758±463; (2.5; 0.7)	321±188; (0.4; 0.7)	305±177; (0.4; 0.7)	Q1LLA8 Ribosomal RNA large subunit methyltransferase J
**phoA: not found**								
**gig: not found *gigB, gigA, gigP, rsqA* .**								
Rmet_4682	*gigT*	NF	72; (71.9; 70.9)	NF	NF	123±113; (123.4; 1.1)	NF	Q1LE82 DoxX
Rmet_4686	*rpoQ*	27	NF	NF	NF	NF	NF	Q1LE78 Sigma-24 (FecI-like)
**zur operon**								
Rmet_0125	*cobW3*	467±138	276±84; (0.6; 0.9)	600±167; (1.3; 0.4)	329±157; (0.7; 0.5)	167±72; (0.5; 0.7)	397±48; (1.2; 0.3)	Q1LS65 Cobalamin synthesis CobW-like protein
Rmet_0126	*dksA*	3979±3308	6214±3922; (1.6; 0.3)	5796±2957; (1.5; 0.3)	1920±1218; (0.5; 0.5)	3043±2887; (1.6; 0.3)	1919±1313; (1.0; 0.0)	Q1LS64 Transcriptional regulators, TraR/DksA family
Rmet_0127	*cobW2*	1419±350	1357±815; (1.0; 0.1)	3108±1198; (2.2; 1.1)	1606±197; (1.1; 0.3)	1138±702; (0.7; 0.5)	2682±1095; (1.7; 0.8)	Q1LS63 Cobalamin synthesis protein, P47K
Rmet_0128	*zur*	374±115	347±196; (0.9; 0.1)	261±28; (0.7; 0.8)	332±126; (0.9; 0.2)	166±104; (0.5; 0.7)	321±32; (1.0; 0.1)	Q1LS62 Putative ferric uptake regulator, FUR family
**cobW1 operon: not found *cysS, Rmet_1101, allB, Rmet_1104* .**								
Rmet_1098	*cobW1*	NF	20; (19.9; 18.9)	967±374; (967.1; 2.6)	NF	NF	614±368; (614.4; 1.7)	Q1LPE2 Cobalamin synthesis protein, P47K
Rmet_1099	*folE_IB2*	NF	NF	77±45; (77.3; 1.7)	NF	NF	85; (85.3; 84.3)	Q1LPE1 UPF0343 protein Rmet_1099
Rmet_1102		NF	NF	NF	NF	NF	11±7; (11.0; 1.3)	Q1LPD8 Carbonic anhydrases/acetyltransferase isoleucine patch superfamily
**other folEs:**								
Rmet_3990	*folE_IA*	86±50	124±81; (1.4; 0.3)	120±44; (1.4; 0.4)	119±61; (1.4; 0.3)	116±67; (1.0; 0.0)	122±45; (1.0; 0.0)	Q1LG68 GTP cyclohydrolase
Rmet_2614	*folE_IB1*	914±356	1237±531; (1.4; 0.4)	1081±257; (1.2; 0.3)	738±54; (0.8; 0.4)	1551±383; (2.1; 1.9)	1042±632; (1.4; 0.4)	Q1LK35 UPF0343 protein Rmet_2614
**uptake systems systems: not found *zupT, hoxN, mgtB* .**								
Rmet_3052	*corA1*	394±242	NF	NF	371; (0.9; 0.1)	NF	NF	Q1LIV2 Magnesium and cobalt transport protein CorA
Rmet_0036	*corA2*	391	NF	NF	195±128; (0.5; 1.5)	NF	229; (1.2; 0.3)	Q1LSF4 Mg2+ transporter protein, CorA-like protein
Rmet_3287	*corA3*	162±96	180; (1.1; 0.2)	108±63; (0.7; 0.3)	67; (0.4; 1.0)	41±26; (0.6; 1.0)	127±77; (1.9; 0.8)	Q1LI67 Mg2+ transporter protein, CorA-like protein
Rmet_1973	*pitA*	NF	NF	NF	209; (208.6; 207.6)	NF	NF	Q1LLX4 Phosphate transporter
Rmet_5396	*mgtA*	29±17	32; (1.1; 0.2)	26±15; (0.9; 0.1)	22; (0.8; 0.4)	NF	28±17; (1.3; 0.4)	Q1LC71 ATPase, E1-E2 type
Rmet_0549	*zntB*	82±22	79; (1.0; 0.2)	68±19; (0.8; 0.3)	80±52; (1.0; 0.0)	186; (2.3; 2.0)	51±30; (0.6; 0.3)	Q1LQZ1 Mg2+ transporter protein, CorA-like protein
Rmet_5890	*feoB*	212±69	292±75; (1.4; 0.6)	1176±699; (5.6; 1.3)	311±118; (1.5; 0.5)	443±432; (1.4; 0.2)	588±216; (1.9; 0.8)	Q1LAS7 Ferrous iron transport protein B
Rmet_5891	*feoA*	194	NF	246±142; (1.3; 0.4)	NF	178; (177.5; 176.5)	125±72; (125.0; 1.7)	Q1LAS6 FeoA
**Siderophore: not found *Rmet_1114* .**								
Rmet_1110	*lysA*	NF	NF	458±265; (458.2; 1.7)	NF	290±261; (290.5; 1.1)	370±261; (370.4; 1.4)	Q1LPD0 Orn/DAP/Arg decarboxylase 2
Rmet_1111	*hpcH*	NF	19; (19.4; 18.4)	83±50; (83.4; 1.6)	NF	32; (32.0; 31.0)	105±99; (105.2; 1.0)	Q1LPC9 HpcH/HpaI aldolase
Rmet_1112		158±93	185; (1.2; 0.3)	4146±2512; (26.2; 1.5)	NF	1553±1645; (1552.5; 0.9)	781±917; (781.4; 0.8)	Q1LPC8 IucA/IucC
Rmet_1113		123±69	599; (4.9; 6.9)	2169±1641; (17.6; 1.2)	NF	831±567; (830.7; 1.5)	2115±2115; (2115.3; 1.0)	Q1LPC7 IucA/IucC
Rmet_1115		NF	NF	2454±1513; (2453.8; 1.6)	NF	1229; (1229.5; 1228.5)	1987; (1987.1; 1986.1)	Q1LPC5 IucA/IucC
Rmet_1116	*ocd*	169±61	378±214; (2.2; 0.8)	2224±1997; (13.2; 1.0)	205±117; (1.2; 0.2)	1153±116; (5.6; 4.1)	1932±1288; (9.4; 1.2)	Q1LPC4 Ornithine cyclodeaminase
Rmet_1117	*cysK*	67±15	492±436; (7.3; 0.9)	3331±2303; (49.8; 1.4)	107; (1.6; 2.6)	941±379; (8.8; 2.2)	3646±2887; (34.1; 1.2)	Q44004 Cysteine synthase
Rmet_1118	*aleB*	287±186	228±130; (0.8; 0.2)	1400±1011; (4.9; 0.9)	105±79; (0.4; 0.7)	596±581; (5.7; 0.7)	463±283; (4.4; 1.0)	Q1LPC2 TonB-dependent siderophore receptor
Rmet_1119	*rsiA*	NF	NF	87±51; (87.5; 1.7)	NF	NF	46; (46.3; 45.3)	Q1LPC1 Putative FecR
Rmet_1120	*rpoI*	NF	NF	111±67; (110.9; 1.6)	NF	17; (16.8; 15.8)	NF	Q1LPC0 Sigma-24 (FecI-like)
Rmet_1121	*acrD*	219	NF	NF	NF	NF	493; (493.1; 492.1)	Q1LPB9 Hydrophobe/amphiphile efflux-1 HAE1
**Efflux systems: not found *cadA, pbrA, czcD* .**								
Rmet_4594	*zntA*	NF	3425±2300; (3424.7; 1.5)	NF	502; (502.3; 501.3)	NF	NF	Q1LEH0 Heavy metal translocating P-type ATPase
Rmet_5970	*czcP*	31±19	76; (2.4; 2.4)	NF				Q1LAJ7 Heavy metal translocating P-type ATPase
Rmet_3524	*cupA*	309±184	2640±1113; (8.6; 1.8)	142; (0.5; 0.9)	185±103; (0.6; 0.4)	858±775; (4.6; 0.8)	182±114; (1.0; 0.0)	Q1LHI0 Heavy metal translocating P-type ATPase
Rmet_6119	*copF*	195±116	768±701; (3.9; 0.7)	46±21; (0.2; 1.1)				Q58AE3 Cation-transporting p-atpase (Heavy metal translocating P-type ATPase)
Rmet_2379	*ctpA1*	NF	NF	NF	NF	NF	NF	Q8GQ88 Putative metal transporter ATPase
Rmet_2046	*rdxI*	155	NF	NF	NF	NF	NF	Q1LLQ1 Heavy metal translocating P-type ATPase
Rmet_0198	*dmeF*	221±129	NF	NF	503; (2.3; 2.2)	NF	NF	Q1LRZ2 Cation diffusion facilitator family transporter
Rmet_3406	*fieF*	209±93	258±150; (1.2; 0.2)	201±116; (1.0; 0.0)	154±82; (0.7; 0.3)	103; (0.7; 0.6)	138±61; (0.9; 0.1)	Q1LHU8 Cation diffusion facilitator family transporter
Rmet_6211	*cnrT*	11	NF	NF				Q9L3G0 CnrT protein
Rmet_0391	*atmA*	54	NF	NF	NF	NF	NF	Q1LRE9 ABC transporter-related protein
**TonB-dependent OM-proteins: not found *Rmet_1104, 1108, 1819. 3077, 3999, ffcA2, ffcA1, Rmet_5373, fecA1, fecA2* .**								
Rmet_0123		154±34	**37; (0.2; 3.4)**	**550±114; (3.6; 2.7)**	90±81; (0.6; 0.6)	34; (0.4; 0.7)	**622±236; (6.9; 1.7)**	Q1LS67 TonB-dependent receptor
Rmet_0837		NF	71; (70.7; 69.7)	4189±3450; (4189.3; 1.2)	NF	590±554; (589.8; 1.1)	1384±968; (1384.2; 1.4)	Q1LQ53 TonB-dependent siderophore receptor
Rmet_1118	*aleB*	287±186	228±130; (0.8; 0.2)	1400±1011; (4.9; 0.9)	105±79; (0.4; 0.7)	596±581; (5.7; 0.7)	463±283; (4.4; 1.0)	Q1LPC2 TonB-dependent siderophore receptor
Rmet_2277	*tonB*	454	233±88; (0.5; 2.5)	2381±1933; (5.2; 1.0)		319±239; (1.7; 0.5)	664±399; (3.5; 1.2)	Q1LL20 TonB-like protein
Rmet_2676	*tolA*	116±54	NF	66; (0.6; 0.9)	91; (0.8; 0.5)	92±68; (1.0; 0.0)	75±58; (0.8; 0.3)	Q1LJX7 TonB-like protein
Rmet_2789	*btuB*	446±288	159±31; (0.4; 0.9)	437±84; (1.0; 0.0)	459±317; (1.0; 0.0)	457±241; (1.0; 0.0)	308±33; (0.7; 0.4)	Q1LJL4 TonB-dependent receptor, plug
Rmet_3055		70	NF	NF	190±113; (2.7; 1.1)	NF	126±76; (0.7; 0.3)	Q1LIU9 TonB-like protein
Rmet_4565		553	246±153; (0.4; 2.0)	369±274; (0.7; 0.7)		NF	NF	Q1LEJ9 TonB-dependent receptor

Rmet_4607	*oprC*	62±44	17; (0.3; 1.0)	158±79; (2.6; 0.8)	NF	72; (72.0; 71.0)	65; (65.3; 64.3)	Q1LEF7 TonB-dependent copper receptor
Rmet_4617	*piuA*	90		713±489; (7.9; 1.3)		396±301; (5.6; 1.1)	160±104; (2.3; 0.9)	Q1LEE7 TonB-dependent siderophore receptor
Rmet_5340	*hlyB*	NF	7; (7.4; 6.4)	NF	11; (10.7; 9.7)	NF		Q1LCC7 TonB box-like protein
**Identified porins (3 out of 33)**								
Rmet_1628		NF	218; (218.3; 217.3)	93; (93.5; 92.5)	156; (155.6; 154.6)	NF	NF	Q1LMW5 Porin, Gram-negative type
Rmet_3234		1793±571	1430±1469; (0.8; 0.2)	1181±903; (0.7; 0.4)	1743±1635; (1.0; 0.0)	2006±1541; (1.2; 0.1)	1472±336; (0.8; 0.1)	Q1LIC0 Porin, Gram-negative type
Rmet_4547		19±11	8; (0.4; 1.0)	NF	NF	20; (20.1; 19.1)	15; (15.1; 14.1)	Q1LEL4 Putative outer membrane porin signal peptide protein
**Identified proteins involved in chemotaxis and motility (22 out of 48)**								
Rmet_3678		NF	NF	NF	192; (192.4; 191.4)	NF	NF	Q1LH26 Methyl-accepting chemotaxis sensory transducer
Rmet_3681	*cheW*	63±41	26; (0.4; 0.9)	27; (0.4; 0.9)	174±80; (2.8; 0.9)	59±21; (0.3; 1.1)	119±12; (0.7; 0.6)	Q1LH23 CheW protein
Rmet_3682	*aer*	159	NF	NF	323±194; (2.0; 0.8)	NF	NF	Q1LH22 Methyl-accepting chemotaxis sensory transducer with Pas/Pac sensor
Rmet_3683		216	NF	NF	842±289; (3.9; 2.2)	345±226; (0.4; 1.0)	343±198; (0.4; 1.0)	Q1LH21 Methyl-accepting chemotaxis sensory transducer
Rmet_3690	*cheW*	NF	NF	NF	134; (133.9; 132.9)	NF	42±29; (0.3; 3.2)	Q1LH14 CheW protein
Rmet_3691	*cheR*	11	NF	NF	NF	NF	NF	Q1LH13 MCP methyltransferase, CheR-type
Rmet_3695	*cheZ*	72±67	78±19; (1.1; 0.1)	57±12; (0.8; 0.2)	278±137; (3.8; 1.0)	85±49; (0.3; 1.0)	81±57; (0.3; 1.0)	Q1LH09 Chemotaxis phosphatase, CheZ
Rmet_3732	*flgM*	NF	NF	NF	NF	NF	16; (15.6; 14.6)	Q1LGX2 Anti-sigma-28 factor, FlgM
Rmet_3733	*flgA*	NF	30±17; (29.9; 1.6)	20; (20.2; 19.2)	72±42; (72.2; 1.7)	27±16; (0.4; 0.8)	40±28; (0.5; 0.5)	Q1LGX1 Flageller protein FlgA
Rmet_3737	*flgE*	NF	NF	NF	59±3; (58.8; 13.5)	NF	16; (0.3; 13.1)	Q1LGW7 Putative uncharacterized protein
Rmet_3741	*flgI*	NF	NF	NF	131±83; (130.7; 1.5)	NF	NF	Q1LGW3 Flagellar P-ring protein
Rmet_3743	*flgK*	28±16	40; (1.4; 0.7)	NF	103±63; (3.6; 0.9)	NF	52±19; (0.5; 0.6)	Q1LGW1 Flagellar hook-associated protein
Rmet_3744	*flgL*	NF	10; (10.4; 9.4)	NF	36±23; (35.6; 1.4)	NF	41; (1.1; 0.2)	Q1LGW0 Flagellin-like protein
Rmet_4185	*cheD*	57±35	23; (0.4; 1.0)	NF	96±48; (1.7; 0.5)	27; (0.3; 1.4)	54±33; (0.6; 0.5)	Q1LFM4 Methyl-accepting chemotaxis sensory transducer
Rmet_4195	*cheD*	21	NF	NF	44±20; (2.1; 1.1)	NF	25±14; (0.6; 0.6)	Q1LFL4 Methyl-accepting chemotaxis sensory transducer
Rmet_5250		91±53	60; (0.7; 0.6)	NF	374±220; (4.1; 1.0)	NF	NF	Q1LCL7 Methyl-accepting chemotaxis sensory transducer
Rmet_5254	*fliD2*	NF	12; (12.4; 11.4)	NF	22±13; (21.7; 1.4)	NF	10; (0.5; 0.8)	Q1LCL3 Flagellar hook-associated 2-like protein
Rmet_5262	*fliG*	NF	NF	NF	1084±722; (1083.6; 1.5)	NF	NF	Q1LCK5 Flagellar motor switch protein FliG
Rmet_5297	*fliL*	73	NF	NF	144±145; (2.0; 0.5)	NF	NF	Q1LCH0 Flagellar basal body-associated protein FliL
Rmet_5612	*tar*	91±56	34; (0.4; 1.0)	23; (0.3; 1.2)	202±54; (2.2; 1.0)	60; (0.3; 2.6)	81±62; (0.4; 1.1)	Q1LBK5 Methyl-accepting chemotaxis sensory transducer
Rmet_5642	*fliD3*	328	NF	NF	NF	NF	NF	Q1LBH5 Flagellar hook-associated 2-like protein
Rmet_5935		NF	NF	NF	89±63; (88.9; 1.4)	34; (0.4; 0.9)	NF	Q1LAN2 Methyl-accepting chemotaxis sensory transducer
**membrane-bound hydrogenase: not found *hypC1, hypF1, hypA1, hoxT, hoxR, hoxL, hoxM, hoxK, Rmet_1294*.**								
Rmet_1281	*hypE1*	263±152	NF	NF	NF	NF	NF	Q1LNW0 Hydrogenase expression/formation protein HypE
Rmet_1282	*hypD1*	189±109	NF	NF	NF	NF	NF	Q1LNV9 Hydrogenase expression/formation protein HypD
Rmet_1285	*hypB1*	352±211	NF	NF	NF	NF	NF	Q1LNV6 Hydrogenase accessory protein HypB
Rmet_1287	*hoxV*	44±26	NF	NF	NF	NF	NF	Q1LNV4 Putative uncharacterized protein
Rmet_1290	*hoxQ*	598±444	NF	NF	NF	NF	NF	Q1LNV1 HupH hydrogenase expression protein
Rmet_1291	*hoxO*	434±297	NF	NF	NF	NF	NF	Q1LNV0 Hydrogenase-1 expression HyaE
Rmet_1295	*hoxZ*	2033±1187	NF	NF	NF	NF	NF	Q1LNU6 Nickel-dependent hydrogenase b-type cytochrome subunit
Rmet_1297	*hoxG*	3060±1859	NF	NF	NF	NF	NF	Q1LNU4 Nickel-dependent hydrogenase, large subunit
Rmet_1298	*hoxK*	2005±1232	NF	NF	NF	NF	NF	Q1LNU3 Hydrogenase (NiFe) small subunit (HydA)
**soluble hydrogenase and Calvin cycle proteins (17 out of 47 found)**								
Rmet_1498	*cbbO*	584±359	NF	NF	NF	NF	NF	Q1LN95 von Willebrand factor, type A
Rmet_1499	*cbbQ*	1412±829	NF	NF	NF	NF	NF	Q1LN94 ATPase associated with various cellular activities, AAA_5
Rmet_1500	*cbbS*	855±523	NF	NF	NF	NF	NF	Q1LN93 Ribulose bisphosphate carboxylase small chain
Rmet_1501	*cbbL*	8275±4780	NF	NF	NF	NF	NF	Q1LN92 Ribulose bisphosphate carboxylase large chain
Rmet_1512	*cbbP*	123±88	NF	NF	NF	NF	NF	Q1LN81 Phosphoribulokinase
Rmet_1513	*cbbT1*	133±86	NF	NF	NF	NF	NF	Q1LN80 Transketolase
Rmet_1518	*cbbA2*	253±148	NF	NF	NF	NF	NF	Q1LN75 Fructose-bisphosphate aldolase
Rmet_1522	*hoxF*	1396±849	NF	NF	NF	NF	NF	Q1LN71 Respiratory-chain NADH dehydrogenase domain, 51 kDa subunit
Rmet_1523	*hoxU*	1197±755	NF	NF	NF	NF	NF	Q1LN70 Ferredoxin
Rmet_1524	*hoxY*	1437±963	NF	NF	NF	NF	NF	Q1LN69 NADH ubiquinone oxidoreductase, 20 kDa subunit
Rmet_1525	*hoxH*	2553±1474	NF	NF	NF	NF	NF	Q1LN68 Nickel-dependent hydrogenase, large subunit
Rmet_1526	*hoxW*	517±354	NF	NF	NF	NF	NF	Q1LN67 HoxW protein
Rmet_1535	*hypA2*	199±127	NF	NF	NF	NF	NF	Q1LN58 Hydrogenase nickel insertion protein HypA
Rmet_1537	*hypF2*	882±570	NF	NF	NF	NF	NF	Q1LN56 (NiFe) hydrogenase maturation protein HypF
Rmet_1539	*hypD2*	1946±1344	NF	NF	NF	NF	NF	Q1LN54 Hydrogenase expression/formation protein HypD
Rmet_1540	*hypE2*	1286±743	NF	NF	NF	NF	NF	Q1LN53 Hydrogenase expression/formation protein HypE
Rmet_1541	*hoxX*	188±108	NF	NF	NF	NF	NF	Q1LN52 Formyl transferase-like protein
**Oxidative phosphorylation (37 our of 57 found)**								
Rmet_0261	*coxB*	3007±2014	2488±1589; (0.8; 0.1)	1229±1255; (0.4; 0.5)	2350±288; (0.8; 0.3)	1770±869; (0.8; 0.5)	2425±721; (1.0; 0.1)	Q1LRS9 Cytochrome c oxidase subunit 2
Rmet_0262	*coxA*	1005±648	984; (1.0; 0.0)	NF	932; (0.9; 0.1)	NF	NF	Q1LRS8 Cytochrome c oxidase, subunit I
Rmet_0263	*ctaG*	NF	NF	NF	NF	NF	11; (11.1; 10.1)	Q1LRS7 Cytochrome c oxidase assembly protein CtaG/Cox11
Rmet_0265	*coxC*	291±211	421±276; (1.4; 0.3)	537±514; (1.8; 0.3)	880; (3.0; 2.8)	NF	NF	Q1LRS5 Cytochrome c oxidase, subunit III
Rmet_0269	*ctaA*	137	NF	523±315; (3.8; 1.2)	108±70; (0.8; 0.4)	556±517; (5.1; 0.8)	NF	Q1LRS1 Cytochrome oxidase assembly

Rmet_0270	*ctaB*	NF	NF	NF	NF	NF	NF	Q1LRS0 Protoheme IX farnesyltransferase
Rmet_0678	*ppa*	1151±478	1166±280; (1.0; 0.0)	883±88; (0.8; 0.5)	697±360; (0.6; 0.5)	928±244; (1.3; 0.4)	654±87; (0.9; 0.1)	Q1LQL2 Inorganic pyrophosphatase
Rmet_0927	*nuoA*	881±268	515±310; (0.6; 0.6)	1278±640; (1.5; 0.4)	1094±990; (1.2; 0.2)	907±827; (0.8; 0.1)	428±108; (0.4; 0.6)	Q1LPW3 NADH-quinone oxidoreductase subunit
Rmet_0928	*nuoB*	2669±755	1104±727; (0.4; 1.1)	1983±47; (0.7; 0.9)	1543±63; (0.6; 1.4)	665±297; (0.4; 2.4)	1460±407; (0.9; 0.2)	Q1LPW2 NADH-quinone oxidoreductase subunit B
Rmet_0929	*nuoC*	4050±2160	2919±1694; (0.7; 0.3)	5030±625; (1.2; 0.4)	3710±1004; (0.9; 0.1)	3074±1089; (0.8; 0.3)	5743±4537; (1.5; 0.4)	Q1LPW1 NADH-quinone oxidoreductase subunit C
Rmet_0930	*nuoD*	4564±2033	2557±1218; (0.6; 0.6)	2489±134; (0.5; 1.0)	4004±2089; (0.9; 0.1)	1308±415; (0.3; 1.1)	3189±672; (0.8; 0.3)	Q1LPW0 NADH dehydrogenase I, D subunit
Rmet_0931	*nuoE*	1566±782	915±155; (0.6; 0.7)	1149±229; (0.7; 0.4)	1471±1038; (0.9; 0.1)	840±453; (0.6; 0.4)	842±124; (0.6; 0.5)	Q1LPV9 NADH-quinone oxidoreductase, E subunit
Rmet_0932	*nuoF*	3069±2288	1255±641; (0.4; 0.6)	1468; (0.5; 0.7)	2144±1343; (0.7; 0.3)	1329±819; (0.6; 0.4)	2045±1007; (1.0; 0.0)	Q1LPV8 NADH-quinone oxidoreductase, F subunit
Rmet_0933	*nuoG*	2952±1192	1981±671; (0.7; 0.5)	2187±241; (0.7; 0.5)	2769±1496; (0.9; 0.1)	1675±166; (0.6; 0.7)	2105±75; (0.8; 0.4)	Q1LPV7 NADH-quinone oxidoreductase
Rmet_0934	*nuoH*	1972±1562	858±403; (0.4; 0.6)	1544±886; (0.8; 0.2)	684±437; (0.3; 0.6)	801±708; (1.2; 0.1)	411±297; (0.6; 0.4)	Q1LPV6 NADH-quinone oxidoreductase subunit H
Rmet_0935	*nuoI*	1012±153	908; (0.9; 0.7)	430±316; (0.4; 1.2)	670±225; (0.7; 0.9)	330±156; (0.5; 0.9)	730±217; (1.1; 0.1)	Q1LPV5 NADH-quinone oxidoreductase subunit I
Rmet_0936	*nuoJ*	171±179	275±226; (1.6; 0.3)	592±357; (3.5; 0.8)	129±121; (0.8; 0.1)	254±341; (2.0; 0.3)	93±9; (0.7; 0.3)	Q1LPV4 NADH-ubiquinone/plastoquinone oxidoreductase, chain 6
Rmet_0937	*nuoK*	447	NF	NF	NF	NF	NF	Q1LPV3 NADH-ubiquinone oxidoreductase, chain 4L
Rmet_0938	*nuoL*	1329±471	755±307; (0.6; 0.7)	1358±452; (1.0; 0.0)	1294±1211; (1.0; 0.0)	956±911; (0.7; 0.2)	764±262; (0.6; 0.4)	Q1LPV2 Proton-translocating NADH-quinone oxidoreductase, chain L
Rmet_0939	*nuoM*	383±356	153±53; (0.4; 0.6)	347±240; (0.9; 0.1)	573; (1.5; 0.5)	NF	77±47; (0.1; 10.6)	Q1LPV1 Proton-translocating NADH-quinone oxidoreductase, chain M
Rmet_0940	*nuoN*	832±795	354±258; (0.4; 0.5)	566±576; (0.7; 0.2)	271±257; (0.3; 0.5)	798; (2.9; 2.1)	83; (0.3; 0.7)	Q1LPV0 Proton-translocating NADH-quinone oxidoreductase, chain N
Rmet_0948	*cyoA*	1547±455	1314±415; (0.8; 0.3)	1059±834; (0.7; 0.4)	1370±453; (0.9; 0.2)	1052±543; (0.8; 0.3)	1148±179; (0.8; 0.4)	Q1LPU2 Ubiquinol oxidase, subunit II
Rmet_0949	*cyoB*	1166±630	690±400; (0.6; 0.5)	1533±729; (1.3; 0.3)	1105±889; (0.9; 0.0)	873±687; (0.8; 0.1)	614±306; (0.6; 0.4)	Q1LPU1 Cytochrome c oxidase, subunit I
Rmet_2041	*ccoP*	821±461	364±197; (0.4; 0.7)	928±448; (1.1; 0.1)	819±550; (1.0; 0.0)	187; (0.2; 1.1)	447±285; (0.5; 0.4)	Q1LLQ6 Cytochrome c oxidase, cbb3-type, subunit III
Rmet_2043	*ccoO*	3580±1652	2013±1845; (0.6; 0.4)	1639±1522; (0.5; 0.6)	3730±2462; (1.0; 0.0)	1614; (0.4; 0.9)	3006±939; (0.8; 0.2)	Q1LLQ4 Cytochrome c oxidase, cbb3-type, subunit II
Rmet_2044	*ccoN*	311±339	238±140; (0.8; 0.2)	191; (0.6; 0.4)	671±711; (2.2; 0.3)	93; (0.1; 0.8)	192±115; (0.3; 0.6)	Q1LLQ3 Cytochrome c oxidase, cbb3-type, subunit I
Rmet_2375	*ccoN*	NF	NF	388±156; (388.1; 2.5)	49; (49.0; 48.0)	NF	NF	Q8GQ84 Putative cytochrome-c oxidase
Rmet_2376	*ccoO*	225±146	65; (0.3; 1.1)	862±54; (3.8; 3.2)	197±116; (0.9; 0.1)	220; (1.1; 0.2)	137±38; (0.7; 0.4)	Q8GQ85 Putative cytochrome-c oxidase
Rmet_2623	*ndh*	NF	NF	NF	2175; (2175.1; 2174.1)	NF	NF	Q1LK26 FAD-dependent pyridine nucleotide-disulphide oxidoreductase
Rmet_3228	*petC*	2246±1020	1089±374; (0.5; 0.8)	1945±608; (0.9; 0.2)	1192±315; (0.5; 0.8)	983±801; (0.8; 0.2)	985±400; (0.8; 0.3)	Q1LIC6 Cytochrome c1
Rmet_3229	*petB*	4110±3022	3159±334; (0.8; 0.3)	3390±1832; (0.8; 0.1)	2552±1991; (0.6; 0.3)	1736±1475; (0.7; 0.2)	1893±910; (0.7; 0.2)	Q1LIC5 Cytochrome b
Rmet_3230	*petA*	2472±1554	2041±399; (0.8; 0.2)	1758±544; (0.7; 0.3)	1989±662; (0.8; 0.2)	982±294; (0.5; 1.1)	1387±179; (0.7; 0.7)	Q1LIC4 Ubiquinol-cytochrome c reductase, iron-sulfur subunit
Rmet_4310		NF	225; (225.2; 224.2)	NF	191±121; (190.5; 1.6)	72; (0.4; 1.0)	213±133; (1.1; 0.1)	Q1LFA0 FAD-dependent pyridine nucleotide-disulphide oxidoreductase
Rmet_4955	*appC*	NF	NF	43±26; (43.1; 1.6)	NF	NF	NF	Q1LDF9 Cytochrome bd ubiquinol oxidase, subunit I
Rmet_5232	*cydA*	293±328	82±48; (0.3; 0.6)	232±170; (0.8; 0.1)	114±70; (0.4; 0.5)	NF	111; (1.0; 0.0)	Q1LCN5 Cytochrome bd ubiquinol oxidase, subunit I
Rmet_5609	*cyoB*	8	NF	NF	NF	NF	9; (9.4; 8.4)	Q1LBK8 Cytochrome c oxidase, subunit I
Rmet_5792	*cyoB*	216	NF	NF	NF	NF	369; (369.2; 368.2)	Q1LB25 Cytochrome c oxidase, subunit I
**F1Fo ATPase: not found *atpI* .**								
Rmet_3493	*atpC*	1103±392	992±112; (0.9; 0.2)	1352±188; (1.2; 0.4)	1158±136; (1.1; 0.1)	1082±548; (0.9; 0.1)	1129±319; (1.0; 0.1)	Q1LHL1 ATP synthase epsilon chain
Rmet_3494	*atpD*	5662±1011	4079±1580; (0.7; 0.6)	3842±1851; (0.7; 0.6)	5966±2166; (1.1; 0.1)	4612±1838; (0.8; 0.3)	7781±2939; (1.3; 0.4)	Q1LHL0 ATP synthase subunit beta
Rmet_3495	*atpG*	3332±1665	2179±617; (0.7; 0.5)	1516±788; (0.5; 0.7)	3480±2009; (1.0; 0.0)	2013±1179; (0.6; 0.5)	2926±1691; (0.8; 0.1)	Q1LHK9 ATP synthase gamma chain
Rmet_3496	*atpA*	4613±909	4300±279; (0.9; 0.3)	5601±1692; (1.2; 0.4)	4048±1795; (0.9; 0.2)	3486±1201; (0.9; 0.2)	4834±1358; (1.2; 0.2)	Q1LHK8 ATP synthase subunit alpha
Rmet_3497	*atpH*	139	NF	NF	253±151; (1.8; 0.8)	272; (1.1; 0.1)	227; (0.9; 0.2)	Q1LHK7 ATP synthase F1, delta subunit
Rmet_3498	*atpF*	4970±4279	2571±389; (0.5; 0.5)	5052±1840; (1.0; 0.0)	2752±1895; (0.6; 0.4)	3514±4060; (1.3; 0.1)	2830±1173; (1.0; 0.0)	Q1LHK6 ATP synthase B chain
Rmet_3499	*atpE*	3061±3128	2717±1597; (0.9; 0.1)	4208±3298; (1.4; 0.2)	1998±1972; (0.7; 0.2)	2191±2322; (1.1; 0.0)	511±198; (0.3; 0.7)	Q1LHK5 ATP synthase F0, C subunit
Rmet_3500	*atpB*	645±821	365±129; (0.6; 0.3)	502; (0.8; 0.2)	410±249; (0.6; 0.2)	132; (0.3; 1.1)	370±221; (0.9; 0.1)	Q1LHK4 ATP synthase A chain
**Protein export: not found *secD* .**								
Rmet_0254	*secB*	1662±414	1640±287; (1.0; 0.0)	1360±60; (0.8; 0.6)	1526±109; (0.9; 0.3)	1360±366; (0.9; 0.3)	1471±262; (1.0; 0.1)	Q1LRT6 Protein-export protein secB
Rmet_0281	*ftsY*	696±409	358±175; (0.5; 0.6)	401; (0.6; 0.7)	795±289; (1.1; 0.1)	862±567; (1.1; 0.1)	1120±269; (1.4; 0.6)	Q1LRQ9 Signal recognition particle-docking protein FtsY
Rmet_0926	*secG*	95	NF	245±150; (2.6; 1.0)	NF	NF	NF	Q1LPW4 Protein translocase subunit secG
Rmet_2420	*lepB*	900±334	571±66; (0.6; 0.8)	826±557; (0.9; 0.1)	580±395; (0.6; 0.4)	666±646; (1.1; 0.1)	511±139; (0.9; 0.1)	Q1LKM9 Signal peptidase I
Rmet_2886	*lspA*	29	NF	NF	NF	NF	NF	Q1LJB7 Lipoprotein signal peptidase
Rmet_2945	*yajC*	7194±3550	4947±393; (0.7; 0.6)	7869±2127; (1.1; 0.1)	5174±1031; (0.7; 0.4)	5643±5050; (1.1; 0.1)	3871±496; (0.7; 0.9)	Q1LJ58 Protein translocase subunit yajC
Rmet_2947	*secF*	1407	537±357; (0.4; 2.4)	1124±695; (0.8; 0.4)	NF	NF	NF	Q1LJ56 Protein translocase subunit secF
Rmet_3094	*ffh*	870±315	453±35; (0.5; 1.2)	780±157; (0.9; 0.2)	620±192; (0.7; 0.5)	816±250; (1.3; 0.4)	649±91; (1.0; 0.1)	Q1LIR0 Signal recognition particle subunit FFH/SRP54 (Srp54)
Rmet_3118	*secA*	1846±1022	1982±1093; (1.1; 0.1)	1496±954; (0.8; 0.2)	2562±330; (1.4; 0.5)	1682±520; (0.7; 1.0)	2227±139; (0.9; 0.7)	Q1LIN6 Protein translocase subunit secA
Rmet_3235	*tatC*	111±78	NF	130±76; (1.2; 0.1)	258; (2.3; 1.9)	NF	NF	Q1LIB9 Sec-independent protein translocase TatC
Rmet_3236	*tatB*	397	NF	116±77; (0.3; 3.7)	NF	NF	NF	Q1LIB8 Sec-independent protein translocase protein tatB homolog
Rmet_3237	*tatA*	1892±1260	1580±330; (0.8; 0.2)	2347±1496; (1.2; 0.2)	1238±374; (0.7; 0.4)	895±157; (0.7; 0.6)	1065±376; (0.9; 0.2)	Q1LIB7 Sec-independent protein translocase protein tatA/E homolog
Rmet_3297	*secY*	4949±3366	3175±157; (0.6; 0.5)	2324; (0.5; 0.8)	NF	1324±1069; (1323.9; 1.2)	1732±1039; (1732.0; 1.7)	Q1LI57 Preprotein translocase secY subunit
Rmet_3340	*secE*	182±106	NF	426±313; (2.3; 0.6)	49; (0.3; 1.3)	NF	NF	Q1LI14 Protein translocase subunit secE/sec61 gamma
Rmet_3613	*yidC*	1346±319	835±150; (0.6; 1.1)	1564±388; (1.2; 0.3)	896±107; (0.7; 1.1)	1184±942; (1.3; 0.3)	905±234; (1.0; 0.0)	Q1LH91 Inner membrane protein oxaA
**Transcription: not all membrane-bound anti-sigma factors found, nor *rpoH, rpoJ, rpoK, rpoM, rpoP* .**								
Rmet_3291	*rpoA*	18074±5306	31130±7883; (1.7; 1.0)	33710±5802; (1.9; 1.4)	39210±8089; (2.2; 1.6)	23419±7969; (0.6; 1.0)	32079±5609; (0.8; 0.5)	Q1LI63 DNA-directed RNA polymerase subunit alpha
Rmet_3334	*rpoB*	5563±1567	2652±302; (0.5; 1.6)	4998±351; (0.9; 0.3)	3967±281; (0.7; 0.9)	3896±249; (1.0; 0.1)	4622±816; (1.2; 0.6)	Q1LI20 DNA-directed RNA polymerase subunit beta
Rmet_3333	*rpoC*	6749±1655	3800±1681; (0.6; 0.9)	6678±2210; (1.0; 0.0)	6686±2534; (1.0; 0.0)	4897±2089; (0.7; 0.4)	5837±2330; (0.9; 0.2)	Q1LI21 DNA-directed RNA polymerase subunit beta'
Rmet_0857	*rpoZ*	155	NF	404±79; (2.6; 3.2)	302±180; (2.0; 0.8)	345±254; (1.1; 0.1)	401±238; (1.3; 0.2)	B3R3N5 DNA-DIRECTED RNA POLYMERASE (OMEGA CHAIN) PROTEIN
Rmet_2606	*rpoD1*	1588±190	1280±326; (0.8; 0.6)	2082±876; (1.3; 0.5)	1205±193; (0.8; 1.0)	1392±644; (1.2; 0.2)	1204±268; (1.0; 0.0)	Q1LK43 RNA polymerase sigma factor
Rmet_4661	*rpoD2*	25	NF	NF	NF	NF	116; (116.1; 115.1)	Q1LEA3 RNA polymerase sigma factor

Rmet_2425	*rpoE*	422±244	279±181; (0.7; 0.3)	529±191; (1.3; 0.2)	278±66; (0.7; 0.5)	609±248; (2.2; 1.1)	376±84; (1.4; 0.7)	Q1LKM4 RNA polymerase sigma factor
Rmet_2423	*rseB*	910±383	474±97; (0.5; 0.9)	720±178; (0.8; 0.3)	502±218; (0.6; 0.7)	695±29; (1.4; 0.8)	581±176; (1.2; 0.2)	Q1LKM6 Sigma E regulatory protein, MucB/RseB
Rmet_1120	*rpoI*	NF	NF	111±67; (110.9; 1.6)	NF	17; (16.8; 15.8)	NF	Q1LPC0 Sigma-24 (FecI-like)
Rmet_1119	*rsiA*	NF	NF	87±51; (87.5; 1.7)	NF	NF	46; (46.3; 45.3)	Q1LPC1 Putative FecR
Rmet_4498	*rsjA*	NF	NF	81±48; (81.0; 1.6)	NF	17; (17.0; 16.0)	NF	Q1LER3 Putative FecR
Rmet_4000	*rskA*	NF	NF	14±8; (14.4; 1.4)	NF	NF	NF	Q1LG58 FecR family protein
Rmet_3280	*rpoL*	45±16	34±23; (0.7; 0.3)	32±10; (0.7; 0.5)	19±11; (0.4; 1.0)	15; (0.8; 0.3)	27±18; (1.4; 0.3)	Q1LI74 Sigma-24 (FecI-like)
Rmet_3279	*rslA*	101±61	NF	59±12; (0.6; 0.6)	53±38; (0.5; 0.5)	26; (0.5; 0.7)	52; (1.0; 0.0)	Q1LI75 Putative transmembrane transcriptional regulator (Anti-sigma factor)
Rmet_0303	*rpoN*	476±307	276±272; (0.6; 0.3)	656±409; (1.4; 0.3)	629±364; (1.3; 0.2)	486±199; (0.8; 0.3)	488±282; (0.8; 0.2)	Q1LRN7 Sigma-54 (RpoN)
Rmet_0597	*rpoO*	53±38	66; (1.2; 0.3)	NF	68; (1.3; 0.4)	NF	NF	Q1LQU3 RNA polymerase sigma factor
Rmet_0596	*rsoA*	97±59	53±34; (0.5; 0.5)	61±35; (0.6; 0.4)	84±53; (0.9; 0.1)	28; (0.3; 1.0)	46; (0.6; 0.7)	Q1LQU4 Putative transmembrane transcriptional regulator (Anti-sigma factor)
Rmet_4686	*rpoQ*	27	NF	NF	NF	NF	NF	Q1LE78 Sigma-24 (FecI-like)
Rmet_0910	*rpoR*	174±93	275±230; (1.6; 0.3)	138±40; (0.8; 0.3)	254±232; (1.5; 0.2)	100; (0.4; 0.7)	110±18; (0.4; 0.6)	Q1LPY0 Sigma-24 (FecI-like)
Rmet_0909	*rsrA*	NF	257; (256.8; 255.8)	NF	155; (155.1; 154.1)	NF		Q1LPY1 Putative transmembrane protein
Rmet_2115	*rpoS*	649±379	769; (1.2; 0.3)	407±197; (0.6; 0.4)	802±512; (1.2; 0.2)	686±400; (0.9; 0.1)	399±274; (0.5; 0.5)	Q1LLI2 RNA polymerase sigma factor
Rmet_3702	*fliA*	75	NF	NF	257±140; (3.4; 1.3)	100±42; (0.4; 0.9)	118±110; (0.5; 0.6)	Q1LH02 RNA polymerase sigma factor
Rmet_6207	*cnrH*	67	NF	NF				P37978 RNA polymerase sigma factor cnrH
Rmet_6206	*cnrX*	44	388±176; (8.8; 2.0)					P37975 Nickel and cobalt resistance protein cnrR
Rmet_2192	*greA*	1305±904	1506±952; (1.2; 0.1)	2212±1272; (1.7; 0.4)	1302±1584; (1.0; 0.0)	1320±1024; (1.0; 0.0)	1455±1168; (1.1; 0.1)	Q1LLA5 Transcription elongation factor
Rmet_0859	*greB*	102±60	158; (1.5; 0.9)	207; (2.0; 1.7)	NF	NF	237; (237.4; 236.4)	Q1LQ31 Transcription elongation factor
Rmet_1981	*lexA*	86±50	76±61; (0.9; 0.1)	144±95; (1.7; 0.4)	75±44; (0.9; 0.1)	102; (1.3; 0.6)	85±12; (1.1; 0.2)	Q1LLW6 LexA repressor
Rmet_2032	*nusA*	4839±3303	4735±3966; (1.0; 0.0)	6423; (1.3; 0.5)	8676±2644; (1.8; 0.6)	6074±3867; (0.7; 0.4)	7500±2432; (0.9; 0.2)	Q1LLR5 NusA antitermination factor
Rmet_2695	*nusB*	762±134	504±153; (0.7; 0.9)	671±121; (0.9; 0.4)	514±109; (0.7; 1.0)	558±267; (1.1; 0.1)	595±125; (1.2; 0.3)	Q1LJV8 N utilization substance protein B homolog
Rmet_3339	*nusG*	3583±4077	7000±1485; (2.0; 0.6)	4872; (1.4; 0.3)	4244±619; (1.2; 0.1)	5490±3380; (1.3; 0.3)	6413±4039; (1.5; 0.5)	Q1LI15 Transcription antitermination protein nusG
Rmet_2135	*rho*	2642±572	1074±1017; (0.4; 1.0)	1639±83; (0.6; 1.5)	2313±942; (0.9; 0.2)	1733±483; (0.7; 0.4)	1836±463; (0.8; 0.3)	Q1LLG2 Transcription termination factor Rho
**Ribosome (51 ouf of 54 found, zinc repository components in bold)**								
Rmet_0410	*rplM*	2284±1476	1329±546; (0.6; 0.5)	2308±1398; (1.0; 0.0)	2799±601; (1.2; 0.2)	4775±3567; (1.7; 0.5)	2304±423; (0.8; 0.5)	Q1LRC9 30S ribosomal protein S9
Rmet_0722	*rpsA*	20078±8677	20357±1930; (1.0; 0.0)	20224±6664; (1.0; 0.0)	40263±6777; (2.0; 1.3)	41184±22486; (1.0; 0.0)	35539±18079; (0.9; 0.2)	Q1LQG8 SSU ribosomal protein S1P
Rmet_0748	*rpsP*	2557±1509	526±312; (0.2; 1.1)	988; (0.4; 1.0)	4653±6038; (1.8; 0.3)	1867±1329; (0.4; 0.4)	1641±415; (0.4; 0.5)	Q1LQE2 SSU ribosomal protein S16P
Rmet_0751	*rplS*	10056±8691	11337±4155; (1.1; 0.1)	11246; (1.1; 0.1)	19497±6173; (1.9; 0.6)	27768±16178; (1.4; 0.4)	13370±3563; (0.7; 0.6)	Q1LQD9 50S ribosomal protein L19
Rmet_0921	***rpsO***	10292±2899	8150±1108; (0.8; 0.5)	8159±3032; (0.8; 0.4)	10970±2491; (1.1; 0.1)	11932±4206; (1.1; 0.1)	8684±2595; (0.8; 0.4)	Q1LPW9 30S ribosomal protein S15
Rmet_1162	*rpmI*	NF	NF	NF	36; (36.1; 35.1)	NF	NF	Q1LP78 50S ribosomal protein L35
Rmet_1163	*rplT*	9880±9037	9384±5015; (0.9; 0.0)	4903±3249; (0.5; 0.4)	11076±7529; (1.1; 0.1)	8972±6200; (0.8; 0.2)	3036±1279; (0.3; 0.9)	Q1LP77 50S ribosomal protein L20
Rmet_1435	***rpsB***	11789±5508	5855±1240; (0.5; 0.9)	12423±2996; (1.1; 0.1)	10049±3151; (0.9; 0.2)	10080±2693; (1.0; 0.0)	8071±1006; (0.8; 0.5)	Q1LNF8 30S ribosomal protein S2
Rmet_1976	*rplI*	17115±4280	17619±7963; (1.0; 0.0)	18454±3934; (1.1; 0.2)	20714±12005; (1.2; 0.2)	29012±20295; (1.4; 0.3)	13866±486; (0.7; 0.5)	Q1LLX1 50S ribosomal protein L9
Rmet_1977	*rpsR*	6861±3990	2459±1594; (0.4; 0.8)	3622; (0.5; 0.8)	6888±3957; (1.0; 0.0)	4322±2610; (0.6; 0.4)	3139±1211; (0.5; 0.7)	Q1LLX0 30S ribosomal protein S18
Rmet_1979	*rpsF*	6127±4139	2641±1841; (0.4; 0.6)	5813; (0.9; 0.1)	7831±3192; (1.3; 0.2)	2451±1417; (0.3; 1.2)	5297±2728; (0.7; 0.4)	Q1LLW8 30S ribosomal protein S6
Rmet_2137		3977±1639	3860±1729; (1.0; 0.0)	4264±517; (1.1; 0.1)	5045±2917; (1.3; 0.2)	4433; (0.9; 0.2)	2881; (0.6; 0.7)	Q1LLG0 50S ribosomal protein L31 type B
Rmet_2432	*rpmF*	286±165	904±134; (3.2; 2.1)	788±289; (2.8; 1.1)	888±631; (3.1; 0.8)	645±204; (0.7; 0.3)	613±335; (0.7; 0.3)	Q1LKL7 50S ribosomal protein L32
Rmet_2455	*rpsU1*	1252±245	882±181; (0.7; 0.9)	1124±586; (0.9; 0.2)	1109±591; (0.9; 0.2)	1426±483; (1.3; 0.3)	1145±269; (1.0; 0.0)	Q46YX7 30S ribosomal protein S21
Rmet_2609	*rpsU2*	109	105±47; (1.0; 0.1)	NF	143±85; (1.3; 0.4)	128±74; (0.9; 0.1)	140±112; (1.0; 0.0)	Q1LK40 30S ribosomal protein S21 2
Rmet_2870	*rpmB*	6505±2093	3862±1358; (0.6; 0.8)	6787±1687; (1.0; 0.1)	5626±1245; (0.9; 0.3)	7217±1987; (1.3; 0.5)	5654±382; (1.0; 0.0)	Q1LJD3 50S ribosomal protein L28
Rmet_2871	*rpmG*	1535±1201	2244±245; (1.5; 0.5)	1677; (1.1; 0.1)	2068±815; (1.3; 0.3)	2145; (1.0; 0.1)	2301±1238; (1.1; 0.1)	Q1LJD2 50S ribosomal protein L33
Rmet_2904	*rpsT*	2691±1205	4064±2418; (1.5; 0.4)	4095±2291; (1.5; 0.4)	3249±1729; (1.2; 0.2)	3494±1795; (1.1; 0.1)	1763±926; (0.5; 0.6)	Q1LJ99 30S ribosomal protein S20
Rmet_3105	*rpmA*	1601±842	656±304; (0.4; 0.8)	1441±620; (0.9; 0.1)	2552±1473; (1.6; 0.4)	2676±480; (1.0; 0.1)	1039±505; (0.4; 0.8)	Q1LIP9 50S ribosomal protein L27
Rmet_3106	*rplU*	7768±3501	11068±8203; (1.4; 0.3)	9945±4540; (1.3; 0.3)	5224±1969; (0.7; 0.5)	9712±2477; (1.9; 1.0)	3426±633; (0.7; 0.7)	Q1LIP8 50S ribosomal protein L21
Rmet_3290	*rplQ*	13808±3143	14835±8634; (1.1; 0.1)	11501±218; (0.8; 0.7)	12067±3426; (0.9; 0.3)	17048±4138; (1.4; 0.7)	7748±1305; (0.6; 0.9)	Q1LI64 50S ribosomal protein L17
Rmet_3292	*rpsD*	17552±6918	14645±5570; (0.8; 0.2)	13991±8252; (0.8; 0.2)	22950±7242; (1.3; 0.4)	27163±16455; (1.2; 0.2)	12874±7277; (0.6; 0.7)	Q1LI62 30S ribosomal protein S4
Rmet_3293	*rpsK*	7432±479	3884±722; (0.5; 3.0)	5874±1648; (0.8; 0.7)	5638±2141; (0.8; 0.7)	5628±2624; (1.0; 0.0)	3679±456; (0.7; 0.8)	Q1LI61 30S ribosomal protein S11
Rmet_3294	*rpsM*	9164±5200	4900±2568; (0.5; 0.5)	13547; (1.5; 0.8)	6896±758; (0.8; 0.4)	11586±6844; (1.7; 0.6)	11103±3481; (1.6; 1.0)	Q1LI60 30S ribosomal protein S13
Rmet_3298	*rplO*	4266±2847	2319±825; (0.5; 0.5)	3512±1082; (0.8; 0.2)	4565±640; (1.1; 0.1)	6066±2282; (1.3; 0.5)	3766±3082; (0.8; 0.2)	Q1LI56 50S ribosomal protein L15
Rmet_3299	*rpmD*	2280±286	1850±414; (0.8; 0.6)	2248±56; (1.0; 0.1)	2268±945; (1.0; 0.0)	2628±209; (1.2; 0.3)	2151±760; (0.9; 0.1)	Q1LI55 50S ribosomal protein L30
Rmet_3300	*rpsE*	2766±1223	2256±106; (0.8; 0.4)	3597±646; (1.3; 0.4)	4554±3566; (1.6; 0.4)	3747±1416; (0.8; 0.2)	3416±530; (0.8; 0.3)	Q1LI54 30S ribosomal protein S5
Rmet_3301	*rplR*	8099±4687	5027±2802; (0.6; 0.4)	8443±1244; (1.0; 0.1)	7781±2510; (1.0; 0.0)	10119±2538; (1.3; 0.5)	5345±95; (0.7; 0.9)	Q1LI53 50S ribosomal protein L18
Rmet_3302	*rplF*	21869±4167	13207±1096; (0.6; 1.6)	15261±1524; (0.7; 1.2)	20965±5644; (1.0; 0.1)	22521±8627; (1.1; 0.1)	15087±5591; (0.7; 0.5)	Q1LI52 50S ribosomal protein L6
Rmet_3303	*rpsH*	6329±3245	6516±275; (1.0; 0.1)	5387; (0.9; 0.3)	6251±2492; (1.0; 0.0)	5349±4059; (0.9; 0.1)	4996±1537; (0.8; 0.3)	Q1LI51 30S ribosomal protein S8
Rmet_3304	*rpsN*	17061±9909	17374±9630; (1.0; 0.0)	21576; (1.3; 0.5)	21644±1877; (1.3; 0.4)	26478±15530; (1.2; 0.3)	21280±10558; (1.0; 0.0)	Q1LI50 SSU ribosomal protein S14P
Rmet_3305	*rplE*	7908±5956	8712±3201; (1.1; 0.1)	4369±4237; (0.6; 0.3)	12210±2510; (1.5; 0.5)	12596±9837; (1.0; 0.0)	8578±475; (0.7; 1.2)	Q1LI49 50S ribosomal protein L5
Rmet_3306	*rplX*	5864±1401	12759±8245; (2.2; 0.7)	8072±1769; (1.4; 0.7)	6293±783; (1.1; 0.2)	9186±2476; (1.5; 0.9)	4722±1411; (0.8; 0.7)	Q1LI48 50S ribosomal protein L24
Rmet_3307	*rplN*	4651±2395	4981±950; (1.1; 0.1)	8756±2015; (1.9; 0.9)	6733±1436; (1.4; 0.5)	8420±3749; (1.3; 0.3)	6576±2477; (1.0; 0.0)	Q1LI47 50S ribosomal protein L14
Rmet_3308	***rpsQ***	6866±2609	5594±1536; (0.8; 0.3)	5248±3796; (0.8; 0.3)	7682±2534; (1.1; 0.2)	9808±4530; (1.3; 0.3)	7349±2288; (1.0; 0.1)	Q1LI46 30S ribosomal protein S17
Rmet_3309	*rpmC*	741±198	3876±2166; (5.2; 1.3)	4375±2016; (5.9; 1.6)	2736±1840; (3.7; 1.0)	4177±1611; (1.5; 0.4)	1428±1035; (0.5; 0.5)	Q1LI45 50S ribosomal protein L29
Rmet_3310	*rplP*	18477±1983	6459±4029; (0.3; 2.0)	9963±3164; (0.5; 1.7)	16532±4872; (0.9; 0.3)	16025±8813; (1.0; 0.0)	10690±3510; (0.6; 0.7)	Q1LI44 50S ribosomal protein L16
Rmet_3311	*rpsC*	16386±6913	11108±4126; (0.7; 0.5)	10975±3696; (0.7; 0.5)	11107±8979; (0.7; 0.3)	7773±2345; (0.7; 0.3)	10107±1187; (0.9; 0.1)	Q1LI43 30S ribosomal protein S3

Rmet_3312	*rplV*	5391±1843	4245±1077; (0.8; 0.4)	5077±1901; (0.9; 0.1)	7324±4116; (1.4; 0.3)	5471±1480; (0.7; 0.3)	3937±987; (0.5; 0.7)	Q1LI42 50S ribosomal protein L22
Rmet_3313	*rpsS*	7756±3283	3311±1071; (0.4; 1.0)	5840±199; (0.8; 0.5)	5813±1121; (0.7; 0.4)	5341±2719; (0.9; 0.1)	4315±500; (0.7; 0.9)	Q1LI41 30S ribosomal protein S19
Rmet_3314	***rplB***	15921±4016	9341±4511; (0.6; 0.8)	9886±2738; (0.6; 0.9)	10855±1193; (0.7; 1.0)	12363±3925; (1.1; 0.3)	7501±788; (0.7; 1.7)	Q1LI40 50S ribosomal protein L2
Rmet_3315	*rplW*	10335±4131	6023±967; (0.6; 0.8)	9612±2623; (0.9; 0.1)	13774±6668; (1.3; 0.3)	22280±9394; (1.6; 0.5)	12362±174; (0.9; 0.2)	B3R7S1 50S ribosomal subunit protein L23
Rmet_3316	*rplD*	28245±2178	23173±10602; (0.8; 0.4)	21349±11896; (0.8; 0.5)	30713±12541; (1.1; 0.2)	46686±28516; (1.5; 0.4)	26245±15047; (0.9; 0.2)	Q1LI38 50S ribosomal protein L4
Rmet_3317	*rplC*	10357±4047	8509±2531; (0.8; 0.3)	9430±911; (0.9; 0.2)	12240±3171; (1.2; 0.3)	19345±5581; (1.6; 0.8)	9324±3362; (0.8; 0.4)	Q1LI37 50S ribosomal protein L3
Rmet_3323	*rpsJ*	4724±1070	5039±1403; (1.1; 0.1)	4979±1363; (1.1; 0.1)	4120±858; (0.9; 0.3)	3638±1096; (0.9; 0.2)	3434±1501; (0.8; 0.3)	B3R7S9 30S ribosomal subunit protein S10
Rmet_3326	*rpsG*	18279±736	6874±3706; (0.4; 2.6)	11420±786; (0.6; 4.5)	11403±887; (0.6; 4.2)	15938±3686; (1.4; 1.0)	12672±3381; (1.1; 0.3)	Q1LI28 30S ribosomal protein S7
Rmet_3327	*rpsL*	7225±2396	5449±592; (0.8; 0.6)	7099±1923; (1.0; 0.0)	6046±3267; (0.8; 0.2)	6185±2730; (1.0; 0.0)	5199±3109; (0.9; 0.1)	Q1LI27 30S ribosomal protein S12
Rmet_3335	*rplL*	3867±2795	4197±2776; (1.1; 0.1)	4252±1439; (1.1; 0.1)	4259±2118; (1.1; 0.1)	3205±2834; (0.8; 0.2)	3306±2717; (0.8; 0.2)	Q1LI19 50S ribosomal protein L7/L12
Rmet_3336	*rplJ*	9453±4067	12583±6638; (1.3; 0.3)	9536±2474; (1.0; 0.0)	9417±4522; (1.0; 0.0)	13800±6222; (1.5; 0.4)	11256±7978; (1.2; 0.1)	Q1LI18 50S ribosomal protein L10
Rmet_3337	*rplA*	14562±2158	10838±3025; (0.7; 0.7)	12692±4333; (0.9; 0.3)	23675±11593; (1.6; 0.7)	24421±12249; (1.0; 0.0)	15932±6917; (0.7; 0.4)	Q1LI17 50S ribosomal protein L1
Rmet_3338	*rplK*	13672±1276	8884±4550; (0.6; 0.8)	10606±1055; (0.8; 1.3)	10467±4804; (0.8; 0.5)	14984±1978; (1.4; 0.7)	7839±1006; (0.7; 0.5)	Q1LI16 50S ribosomal protein L11
**Initiation and release: not found *infA3* .**								
Rmet_3324	*tuf*	29841±1461[illegible]	26177±8758; (0.9; 0.2)	26946±10195; (0.9; 0.1)	30728±7035; (1.0; 0.0)	28871±6391; (0.9; 0.1)	34057±9028; (1.1; 0.2)	Q1LI13 Elongation factor Tu
Rmet_3341	*tuf*	29841±1461[illegible]	26177±8758; (0.9; 0.2)	26946±10195; (0.9; 0.1)	30728±7035; (1.0; 0.0)	28871±6391; (0.9; 0.1)	34057±9028; (1.1; 0.2)	Elongation factor Tu
Rmet_3325	*fusA1*	8016±2136	5513±317; (0.7; 1.0)	7160±943; (0.9; 0.3)	4635±1096; (0.6; 1.0)	6646±2422; (1.4; 0.6)	6596±733; (1.4; 1.1)	Q1LI29 Elongation factor G 1
Rmet_5930	*fusA2*	2784±1805	3143±189; (1.1; 0.2)	1814±421; (0.7; 0.4)	2053±1470; (0.7; 0.2)	1904±824; (0.9; 0.1)	2421±976; (1.2; 0.2)	Q1LAN7 Elongation factor G 2
Rmet_1436	*tsf*	5820±3790	2752±876; (0.5; 0.7)	3815; (0.7; 0.5)	7519±3087; (1.3; 0.2)	6200±3696; (0.8; 0.2)	6192±978; (0.8; 0.3)	Q1LNF7 Elongation factor Ts
Rmet_1161	*infC*	5708±3960	3932±1405; (0.7; 0.3)	4607; (0.8; 0.3)	6943±1354; (1.2; 0.2)	6737±4751; (1.0; 0.0)	6578±3042; (0.9; 0.1)	Q1LP79 Translation initiation factor IF-3
Rmet_2031	*infB*	2170±473	1462±68; (0.7; 1.3)	1615±419; (0.7; 0.6)	1770±797; (0.8; 0.3)	1474±260; (0.8; 0.3)	1442±116; (0.8; 0.4)	Q1LLR6 Translation initiation factor IF-2
Rmet_2174	*infA*	1139±594	1314±226; (1.2; 0.2)	1249±457; (1.1; 0.1)	702±169; (0.6; 0.6)	1367±701; (1.9; 0.8)	1033±167; (1.5; 1.0)	Q1LLC3 Translation initiation factor IF-1 1
Rmet_3296	*infA*	44	233±118; (5.3; 1.6)	149±85; (3.4; 1.2)	71±48; (1.6; 0.6)	169±179; (2.4; 0.4)	72±17; (1.0; 0.0)	Q1LI58 Translation initiation factor IF-1 2
Rmet_1036	*prfB*	660±87	410±73; (0.6; 1.6)	618±46; (0.9; 0.3)	726±127; (1.1; 0.3)	606±181; (0.8; 0.4)	876±326; (1.2; 0.3)	Q1LPK4 Bacterial peptide chain release factor 2 (BRF-2)
Rmet_3201	*prfA*	463±136	354±150; (0.8; 0.4)	434±274; (0.9; 0.1)	466±182; (1.0; 0.0)	414±136; (0.9; 0.2)	401±213; (0.9; 0.2)	Q1LIF3 Peptide chain release factor 1
Rmet_3468		328	NF	NF	NF	945; (944.7; 943.7)	580; (579.5; 578.5)	Q1LHN6 Class I peptide chain release factor
Rmet_5808	*prfC*	NF	873±93; (872.9; 9.2)	1311±79; (1311.4; 16.4)	832±534; (831.9; 1.6)	1423±1263; (1.7; 0.3)	2156±865; (2.6; 0.9)	Q1LB09 Bacterial peptide chain release factor 3 (BRF-3)

Supplementary Table S3. Components of the F_1F_0 ATPase[a].

Locus Tag	Gene	Control (Q, D)	Metal-shocked (Q, D)	Metal-starved (Q, D)	Subunit
**Strain CH34**					
Rmet_3493	*atpC*	1,103±392	992±112; (0.9; 0.2)	1,352±188; (1.2; 0.4)	epsilon chain
Rmet_3494	*atpD*	5,662±1,011	4,079±1,580; (0.7; 0.6)	3,842±1,851; (0.7; 0.6)	subunit beta
Rmet_3495	*atpG*	3,332±1,665	2,179±617; (0.7; 0.5)	1,516±788; (0.5; 0.7)	gamma chain
Rmet_3496	*atpA*	4,613±909	4,300±279; (0.9; 0.3)	5,601±1,692; (1.2; 0.4)	subunit alpha
Rmet_3497	*atpH*	139±0	77	NF	delta subunit
Rmet_3498	*atpF*	4,970±4279	2,571±389; (0.5; 0.5)	5,052±1,840; (1.0; 0.0)	B chain
Rmet_3499	*atpE*	3,061±3128	2,717±1597; (0.9; 0.1)	4,208±3,298; (1.4; 0.2)	C subunit
Rmet_3500	*atpB*	645±821	365±129; (0.6; 0.3)	502±0; (0.8; 0.2)	A chain
**Strain AE104**					
Rmet_3493	*atpC*	1,158±136; (1.1; 0.1)	1,082±548; (0.9; 0.1)	1,129±319; (1.0; 0.1)	epsilon chain
Rmet_3494	*atpD*	5,966±2,166; (1.1; 0.1)	4,612±1,838; (0.8; 0.3)	7,781±2,939; (1.3; 0.4)	subunit beta
Rmet_3495	*atpG*	3,480±2,009; (1.0; 0.0)	2,013±1,179; (0.6; 0.5)	2,926±1,691; (0.8; 0.1)	gamma chain
Rmet_3496	*atpA*	4,048±1,795; (0.9; 0.2)	3,486±1,201; (0.9; 0.2)	4,834±1,358; (1.2; 0.2)	subunit alpha
Rmet_3497	*atpH*	253±151; (1.8; 0.8)	272±0; (1.1; 0.1)	227±0; (0.9; 0.2)	delta subunit
Rmet_3498	*atpF*	2,752±1,895; (0.6; 0.4)	3,514±4,060; (1.3; 0.1)	2,830±1,173; (1.0; 0.0)	B chain
Rmet_3499	*atpE*	1,998±1,972; (0.7; 0.2)	2,191±2,322; (1.1; 0.0)	511±198; (0.3; 0.7)	C subunit
Rmet_3500	*atpB*	410±249; (0.6; 0.2)	132±0; (0.3; 1.1)	370±221; (0.9; 0.1)	A chain

[a]The Table gives the number of the products per cell of the respective gene with deviations, plus the comparisons CH34 with metal mix to without it, and CH34 with EDTA to without treatment. The same representation is given for strain AE104 and for non-challenged control cells AE104/CH34. The Q ratios and the distance values follow these numbers in parentheses. Single values indicate a result only in one out of the three determinations. NF is „Not found“ in any of the three replicates. Comparisons to NF values were not done. Bold-faced would have been Q ratios with D > 1 but none was found.

Supplementary Table S4. Top ten of the appearing or dis-appearing proteins sorted by the copy number before dis-appearance[a]

Locus tag	gene	CH34_0	KO	Description
		**CMO app. in C_M**		
Rmet_3529	*pbpC*	21,895±19,434	NA	Q1LHH5 Penicillin-binding protein 1C
Rmet_6183	*merA*	11,249±2,250	NA	A6UXG5 Mercuric reductase
Rmet_6122	*copH*	5,301±2,837	NA	Q58AE5 CopH protein (Putative uncharacterized protein)
Rmet_6173	*merP*	3,935±1,757	EIP-TRA-Other	Q58AI1 Periplasmic mercuric-ion binding protein
Rmet_6346	*merP*	3,935±1,757	NA	Periplasmic mercuric-ion binding protein
Rmet_4594	*zntA*	3,425±2,300	MET-EN-P_ATPase	Q1LEH0 Heavy metal translocating P-type ATPase
Rmet_0901		2,747	NA	Q1LPY9 Transcriptional regulator, TetR family
Rmet_6116	*copI*	2,274±982	NA	Q58AE0 Putative oxydoreductase (Blue (Type 1) copper domain)
Rmet_2129	*dnaX*	1,784	GIP-REP-DNA_Pol	Q1LLG8 DNA polymerase III, gamma subunit
Rmet_6113	*copB1*	1,559±870	NA	Q58AD7 CopB protein (Copper resistance B)
		**CMO dis. in C_O**		
Rmet_1501	*cbbL*	8,275±4,780	MET-CAH-DiCarb	Q1LN92 Ribulose bisphosphate carboxylase large chain
Rmet_5425		3,998	NA	Q1LC42 Acyl-CoA dehydrogenase-like protein
Rmet_1297	*hoxG*	3,060±1,859	NA	Q1LNU4 Nickel-dependent hydrogenase, large subunit
Rmet_3257	*yrbD*	3,039	EIP-TRA-ABC	Q1LI97 Mammalian cell entry related
Rmet_1525	*hoxH*	2,553±1,474	MET-CAH-DiCarb	Q1LN68 Nickel-dependent hydrogenase, large subunit
Rmet_1295	*hoxZ*	2,033±1,187	NA	Q1LNU6 Nickel-dependent hydrogenase b-type cytochrome subunit
Rmet_1298	*hoxK*	2,005±1,232	NA	Q1LNU3 Hydrogenase (NiFe) small subunit (HydA)
Rmet_1539	*hypD2*	1,946±1,344	GIP-PTL-Fold	Q1LN54 Hydrogenase expression/formation protein HypD
Rmet_1524	*hoxY*	1,437±963	MET-CAH-DiCarb	Q1LN69 NADH ubiquinone oxidoreductase, 20 kDa subunit
Rmet_1499	*cbbQ*	1,412±829	MET-EN-Nitrog	Q1LN94 ATPase associated with various cellular activities, AAA_5
		**CE0 app. in C_E**		
Rmet_3529	*pbpC*	14,078±10,116	NA	Q1LHH5 Penicillin-binding protein 1C
Rmet_0673	*pilL2*	6,285	GIP-PTL-T2S	Q1LQL7 CheA signal transduction histidine kinases
Rmet_0837		4,189±3,450	EIP-TRA-Pores	Q1LQ53 TonB-dependent siderophore receptor
Rmet_0838	*piuC*	2,899±1,687	NA	Q1LQ52 PKHD-type hydroxylase Rmet_0838
Rmet_1115		2,454±1,513	NA	Q1LPC5 IucA/IucC
Rmet_0591		2,382	NA	Q1LQU9 Diguanylate cyclase/phosphodiesterase with PAS/PAC sensor(S)
Rmet_4656		1,992	NA	Q1LEA8 Antibiotic biosynthesis monooxygenase
Rmet_1747		1,872	NA	Q1LMK0 Putative uncharacterized protein
Rmet_5376	*hmuT*	1,642±1,427	EIP-TRA-ABC	Q1LC91 Periplasmic binding protein
Rmet_2281		1,492±989	NA	Q1LL16 Putative uncharacterized protein
		**CE0 dis. in C_E**		
Rmet_1501	*cbbL*	8,275±4,780	MET-CAH-DiCarb	Q1LN92 Ribulose bisphosphate carboxylase large chain
Rmet_5425		3,998	NA	Q1LC42 Acyl-CoA dehydrogenase-like protein
Rmet_1297	*hoxG*	3,060±1,859	NA	Q1LNU4 Nickel-dependent hydrogenase, large subunit
Rmet_1525	*hoxH*	2,553±1,474	MET-CAH-DiCarb	Q1LN68 Nickel-dependent hydrogenase, large subunit

Rmet_1295	*hoxZ*	2,033±1,187	NA	Q1LNU6 Nickel-dependent hydrogenase b-type cytochrome subunit
Rmet_1298	*hoxK*	2,005±1,232	NA	Q1LNU3 Hydrogenase (NiFe) small subunit (HydA)
Rmet_1539	*hypD2*	1,946±1,344	GIP-PTL-Fold	Q1LN54 Hydrogenase expression/formation protein HypD
Rmet_1524	*hoxY*	1,437±963	MET-CAH-DiCarb	Q1LN69 NADH ubiquinone oxidoreductase, 20 kDa subunit
Rmet_1499	*cbbQ*	1,412±829	MET-EN-Nitrog	Q1LN94 ATPase associated with various cellular activities, AAA_5
Rmet_1522	*hoxF*	1,396±849	MET-CAH-DiCarb	Q1LN71 Respiratory-chain NADH dehydrogenase domain, 51 kDa subunit
		**0AC app. in A_0**		
Rmet_4941		4,068	NA	Q1LDH3 Transcriptional regulator, IclR family
Rmet_2129	*dnaX*	2,585±640	GIP-REP-DNA_Pol	Q1LLG8 DNA polymerase III, gamma subunit
Rmet_2623	*ndh*	2,175±	MET-EN-OxPhos	Q1LK26 FAD-dependent pyridine nucleotide-disulphide oxidoreductase
Rmet_0591		1,857	NA	Q1LQU9 Diguanylate cyclase/phosphodiesterase with PAS/PAC sensor(S)
Rmet_4386		1,734	NA	Q1LF25 Putative lipoprotein transmembrane
Rmet_5226		1,212	NA	Q1LCP1 Putative uncharacterized protein
Rmet_5262	*fliG*	1,084±722	DIV-MOT-Chemotax	Q1LCK5 Flagellar motor switch protein FliG
Rmet_0300	*ptsN*	956	EIP-TRA-Pts	Q1LRP0 PTS IIA-like nitrogen-regulatory protein PtsN
Rmet_2379	*ctpA1*	917	MET-EN-P_ATPase	Q8GQ88 Putative metal transporter ATPase
Rmet_5808	*prfC*	832±534	NA	Q1LB09 Bacterial peptide chain release factor 3 (BRF-3)
		**0AC dis. in C_0**		
Rmet_1501	*cbbL*	8,275±4,780	MET-CAH-DiCarb	Q1LN92 Ribulose bisphosphate carboxylase large chain
Rmet_6191	*bph2*	8,000±1,047	NA	Q5NUZ1 Putative uncharacterized protein bph2
Rmet_0697	*pilA*	7,956±1,079	GIP-PTL-T2S	Q1LQJ3 Pilus assembly protein major pilin PilA
Rmet_6090		6,697±3,170	NA	Q1LA77 Histone-like DNA-binding protein
Rmet_3297	*secY*	4,949±3,366	GIP-PTL-Exp	Q1LI57 Preprotein translocase secY subunit
Rmet_5425		3,998	NA	Q1LC42 Acyl-CoA dehydrogenase-like protein
Rmet_1297	*hoxG*	3,060±1,859	NA	Q1LNU4 Nickel-dependent hydrogenase, large subunit
Rmet_1525	*hoxH*	2,553±1,474	MET-CAH-DiCarb	Q1LN68 Nickel-dependent hydrogenase, large subunit
Rmet_1295	*hoxZ*	2,033±1,187	NA	Q1LNU6 Nickel-dependent hydrogenase b-type cytochrome subunit
Rmet_1298	*hoxK*	2,005±1,232	NA	Q1LNU3 Hydrogenase (NiFe) small subunit (HydA)
		**AM0 app. in A_M**		
Rmet_0673	*pilL2*	3346	GIP-PTL-T2S	Q1LQL7 CheA signal transduction histidine kinases
Rmet_0471		1584	NA	Q1LR69 Integral membrane protein TerC
Rmet_3297	*secY*	1324±1069	GIP-PTL-Exp	Q1LI57 Preprotein translocase secY subunit
Rmet_5671	*copA2*	1321±399	NA	Q1LBE6 Copper-resistance protein CopA
Rmet_1115		1229	NA	Q1LPC5 IucA/IucC, siderophore synthesis
Rmet_3468		945	NA	Q1LHN6 Class I peptide chain release factor
Rmet_2281		933	NA	Q1LL16 Putative uncharacterized protein
Rmet_4596	*czcC2*	892±583	NA	Q1LEG8 Outer membrane efflux protein
Rmet_1113		831±567	NA	Q1LPC7 IucA/IucC, siderophore synthesis
Rmet_5106		717	NA	Q1LD11 Transcriptional regulator, LysR family

		**AM0 dis. in A_0**		
Rmet_4941		4,068	NA	Q1LDH3 Transcriptional regulator, IclR family
Rmet_2623	*ndh*	2,175	MET-EN-OxPhos	Q1LK26 FAD-dependent pyridine nucleotide-disulphide oxidoreductase
Rmet_4386		1,734	NA	Q1LF25 Putative lipoprotein transmembrane
Rmet_5226		1,212	NA	Q1LCP1 Putative uncharacterized protein
Rmet_2716	*cpsG*	1,187±871	MET-CAH-FbP	Q1LJT7 Phosphomannomutase
Rmet_5262	*fliG*	1,084±722;	DIV-MOT-Chemotax	Q1LCK5 Flagellar motor switch protein FliG
Rmet_2379	*ctpA1*	917	MET-EN-P_ATPase	Q8GQ88 Putative metal transporter ATPase
Rmet_1609		690	NA	Q1LMY4 Transcriptional regulator, GntR family
Rmet_4590	*arnC*	662±447	NA	Q1LEH4 Glycosyl transferase, family 2
Rmet_4467	*ubiG*	629	NA	Q1LEU4 3-demethylubiquinone-9 3-methyltransferase
		**AE0 app. in A_E**		
Rmet_0673	*pilL2*	5,241	GIP-PTL-T2S	Q1LQL7 CheA signal transduction histidine kinases
Rmet_5425		2,148±1,483	NA	Q1LC42 Acyl-CoA dehydrogenase-like protein
Rmet_1115		1,987	NA	Q1LPC5 IucA/IucC
Rmet_5376	*hmuT*	1,947±1,222;	EIP-TRA-ABC	Q1LC91 Periplasmic binding protein
Rmet_3297	*secY*	1,732±1,039;	GIP-PTL-Exp	Q1LI57 Preprotein translocase secY subunit
Rmet_0837		1,384±968;	EIP-TRA-Pores	Q1LQ53 TonB-dependent siderophore receptor
Rmet_5374		1,077±957;	NA	Q1LC93 Putative uncharacterized protein
Rmet_2830		908±576;	NA	Q1LJH3 Putative uncharacterized protein
Rmet_1426	*smc*	791	DIV-Division	Q1LNG7 Condensin subunit Smc
Rmet_5639		723±519	NA	Q1LBH8 Putative uncharacterized protein
		**AE0 dis. in A_0**		
Rmet_2623	*ndh*	2,175	MET-EN-OxPhos	Q1LK26 FAD-dependent pyridine nucleotide-disulphide oxidoreductase
Rmet_4386		1,734	NA	Q1LF25 Putative lipoprotein transmembrane
Rmet_5226		1,212	NA	Q1LCP1 Putative uncharacterized protein
Rmet_0849		1,202	NA	Q1LQ41 17 kDa surface antigen
Rmet_5262	*fliG*	1,084±722	DIV-MOT-Chemotax	Q1LCK5 Flagellar motor switch protein FliG
Rmet_2379	*ctpA1*	917	MET-EN-P_ATPase	Q8GQ88 Putative metal transporter ATPase
Rmet_1609		690	NA	Q1LMY4 Transcriptional regulator, GntR family
Rmet_5819	*parA*	578±275	DIV-Division	Q1LAZ8 Cobyrinic acid a,c-diamide synthase
Rmet_0198	*dmeF*	503	EIP-TRA-Ion	Q1LRZ2 Cation diffusion facilitator family transporter
Rmet_4594	*zntA*	502	MET-EN-P_ATPase	Q1LEH0 Heavy metal translocating P-type ATPase

The table gives the copy numbers of the protein with the highest abundance appearing (app.) or disappearing (dis.) in CH34 or AE104 cells (C_, A_) under metal-shock or -starvation conditions or control cells (_M, _E, _0) in the comparisons of CH34 cells metal-shocked against control cells (CM0), metal-starved against control cells (CE0), similarly in AE104 cells (AM0, AE0) or comparison of AE104 to CH34 control cells (0AC).

Supplementary Table S5. Top ten of the regulated proteins

locus tag	gene	Copy number (Q, D)		KO	Description
**CM0 up**		**CH34_0**	**CH34_M**		
Rmet_0333	*arsR*	22±13	1,126±371; (51.0; 2.9)	NA	Q1LRK7 Transcriptional regulator, ArsR
Rmet_3620	*degP*	80±48	3,824±194; (47.9; 15.5)	NA	Q1LH84 Peptidase S1C, Do
Rmet_0331	*arsC2*	34±0	1,538±686; (45.2; 2.2)	NA	Q1LRK9 Protein tyrosine phosphatase
Rmet_5982	*czcC*	17±0	391±207; (22.7; 1.8)	EIP-TRA-Ion	P13509 Cobalt-zinc-cadmium resistance protein czcC
Rmet_6111	*copR1*	21±9	322±125; (15.6; 2.2)	EIP-SIG-2Comp	Q58AD5 Two component response transcription regulator
Rmet_3523	*cupR*	38±0	529±114; (13.8; 4.3)	NA	Q1LHI1 Transcriptional regulator, MerR family
Rmet_6201	*chrC*	104±0	1,318±1154; (12.7; 1.1)	NA	P17550 Superoxide dismutase [Fe]
Rmet_3380		18±11	222±139; (12.1; 1.4)	NA	Q1LHX4 Putative signal transduction protein
Rmet_5499		49±38	531	MET-CAH-Pyr	Q1LBW8 Acetyl-CoA acetyltransferase (Thiolase)
Rmet_5981	*czcB*	146±104	1,509±607; (10.4; 1.9)	EIP-TRA-Ion	P13510 Cobalt-zinc-cadmium resistance protein czcB
**CM0 down**		**CH34_0**	**CH34_M**		
Rmet_1970		1,817±1,539	31	NA	Q1LLX7 PhoH-like protein
Rmet_0886		134±110	12	NA	Q1LQ04 Putative uncharacterized protein
Rmet_5777		157±54	21	NA	Q1LB40 Cupin 2, conserved barrel
Rmet_2128		6,901	956±892; (0.1; 6.7)	NA	Q1LLG9 UPF0133 protein Rmet_2128
Rmet_5231		1,086±777	152±21; (0.1; 1.2)	NA	Q1LCN6 Transcriptional regulator, ArsR family
Rmet_5252	*fliC2*	2,561±419	386±77; (0.2; 4.4)	EIP-SIG-2Comp	Q1LCL5 Flagellin-like protein
Rmet_4168	*hpnH*	1,895±783	311±43; (0.2; 1.9)	NA	Q1LFP1 Radical SAM
Rmet_3663	*sbcD*	73±44	13	GIP-REP-Recomb	Q1LH41 Exodeoxyribonuclease I subunit D
Rmet_2206	*gloB*	679	121±33; (0.2; 16.9)	MET-CAH-Pyr	Q1LL91 Beta-lactamase-like protein
Rmet_2131	*addA*	3,133±1864	575±0; (0.2; 1.4)	GIP-REP-Recomb	Q1LLG6 DNA helicase/exodeoxyribonuclease V
**CE0 up**		**CH34_0**	**CH34_E**		
Rmet_1117	*cysK*	67±15	3,331±2303; (49.8; 1.4)	MET-EN-Sulf	Q44004 Cysteine synthase
Rmet_5321	*zniC*	39±24	1,246	NA	Q1LCE6 RND efflux system, outer membrane lipoprotein, Nod[illegible]
Rmet_5330	*zneB*	16	464±52; (28.8; 8.5)	EIP-TRA-Ion	Q1LCD7 Secretion protein HlyD
Rmet_5319	*zniA*	68±43	1,851±914; (27.1; 1.9)	EIP-TRA-Ion	Q1LCE8 Heavy metal efflux pump CzcA
Rmet_1112		158±93	4,146±2512; (26.2; 1.5)	NA	Q1LPC8 IucA/IucC
Rmet_5329	*zneA*	14	318±188; (23.0; 1.6)	EIP-TRA-Ion	Q1LCD8 Heavy metal efflux pump CzcA
Rmet_5320	*zniB*	132±36	2,480±357; (18.8; 6.0)	EIP-TRA-Ion	Q1LCE7 Secretion protein HlyD
Rmet_1113		123±69	2,169±1641; (17.6; 1.2)	NA	Q1LPC7 IucA/IucC
Rmet_5638		364±150	5,968±4260; (16.4; 1.3)	NA	Q1LBH9 Putative uncharacterized protein
Rmet_2278	*exbB1*	829±594	1,2841±9724; (15.5; 1.2)	NA	Q1LL19 MotA/TolQ/ExbB proton channel
**CE0 down**		**CH34_0**	**CH34_E**		
Rmet_1970		1,817±1,539	82	NA	Q1LLX7 PhoH-like protein
Rmet_0016		271±161	17	NA	Q1LSH4 Putative signal-transduction protein with CBS domain[illegible]
Rmet_2206	*gloB*	679±0	104±22; (0.2; 25.6)	MET-CAH-Pyr	Q1LL91 Beta-lactamase-like protein

Rmet_2501	*gstF*	81±47	14	MET-OAA-GSH	Q1LKE8 Glutathione S-transferase-like
Rmet_0383		567±0	103±59; (0.2; 7.8)	MET-LIP-GlycPLip	Q1LRF7 Transphosphatidylase
Rmet_0858	*spoT*	311±182	62	MET-NUC-Pur	Q1LQ32 SpoT
Rmet_1160	*thrS*	724±420	150	MET-AA-GSTr	Q1LP80 Threonyl-tRNA synthetase
Rmet_3002	*mmfD*	539±290	113	NA	Q1LDD4 Conserved hypothetical
Rmet_6119	*copF*	195±116	46±21; (0.2; 1.1)	MET-EN-PATPase	Q58AE3 Cation-transporting P-type ATPase
Rmet_1148	*dctQ*	576±0	139±99; (0.2; 4.4)	NA	Q1LP92 Tripartite ATP-independent periplasmic transporter
**0AC up**		**CH34_0**	**AE104_0**		
Rmet_2982		30±17	238	NA	Q1LJ21 Glyoxalase
Rmet_4275	*bug*	52±49	311	NA	Q1LFD5 Uncharacterized protein UPF0065
Rmet_0092		74	446±268; (6.0; 1.4)	NA	Q1LS98 Histidine kinase
Rmet_2312	*merR*	18	106±84; (5.8; 1.0)	NA	Q8GQ23 Regulatory protein MerR
Rmet_0595	*iorB*	102±64	567±361; (5.6; 1.1)	NA	Q1LQU5 TAT signal
Rmet_5692		3	16±13; (5.3; 1.0)	NA	Q1LBC5 Putative uncharacterized protein
Rmet_4400		120±85	583±305; (4.9; 1.2)	NA	Q1LF11 Putative uncharacterized protein
Rmet_3900	*bug*	46	192±143; (4.2; 1.0)	NA	Q1LGF8 Uncharacterized protein UPF0065
Rmet_3680		26±15	108±42; (4.2; 1.4)	EIP-SIG-2Comp	Q1LH24 CheY-like
Rmet_5250		91±53	374±220; (4.1; 1.0)	DIV-MOT-Chem	Q1LCL7 Chemotaxis sensor
**0AC down**		**CH34_0**	**AE104_0**		
Rmet_2832		373±161	60	NA	Q1LJH1 Putative uncharacterized protein
Rmet_2040	*rdxB*	280±162	47	NA	Q1LLQ7 4Fe-4S ferredoxin
Rmet_0964	*ureG*	614	111±64; (0.2; 7.9)	GIP-PTL-Fold	Q1LPS6 Urease accessory protein ureG
Rmet_2575		432±187	81±47; (0.2; 1.5)	NA	Q1LK74 Peptidase M48, Ste24p
Rmet_0273		957±553	217±19; (0.2; 1.3)	NA	Q1LRR7 Pirin-like protein
Rmet_0947		341±198	78	NA	Q1LPU3 MaoC-like dehydratase
Rmet_5473		238±18	58	NA	Q1LBZ4 Mandelate racemase/
Rmet_2938	*proC*	1349±790	333±160; (0.2; 1.1)	MET-AA-ArgPro	Q1LJ65 Pyrroline-5-carboxylate reductase
Rmet_3340	*secE*	182±106	49	GIP-PTL-Exp	Q1LI14 Protein translocase subunit secE/sec61 gamma
Rmet_0129	*dehH2*	135	37±12; (0.3; 8.1)	MET-XEN-Hex	Q1LS61 Alpha/beta hydrolase fold
**AM0 up**		**AE104_0**	**AE104_M**		
Rmet_0331	*arsC2*	54±37; (1.6; 0.5)	2,605±1,802; (48.7; 1.4)	NA	Q1LRK9 Arsenate reductase
Rmet_0333	*arsR*	28±3; (1.3; 0.4)	578±315; (20.6; 1.7)	NA	Q1LRK7 Transcriptional regulator, ArsR
Rmet_3620	*degP*	70±40; (0.9; 0.1)	1,009±418; (14.5; 2.1)	NA	Q1LH84 Peptidase S1C, Do
Rmet_5672	*copR2*	34±13; (1.3; 0.2)	412±238; (12.0; 1.5)	EIP-SIG-2Comp	Q1LBE5 Two component regulator
Rmet_3525	*cupC*	55±24; (0.7; 0.5)	556±325; (10.2; 1.4)	NA	Q1LHH9 Heavy metal transport/detoxification protein
Rmet_1117	*cysK*	107±0; (1.6; 2.6)	941±379; (8.8; 2.2)	MET-EN-Sulf	Q44004 Cysteine synthase
Rmet_1116	*ocd*	205±117; (1.2; 0.2)	1,153±116; (5.6; 4.1)	MET-AA-ArgPro	Q1LPC4 Ornithine cyclodeaminase
Rmet_4617	*piuA*	71	396±301; (5.6; 1.1)	EIP-TRA-Pores	Q1LEE7 TonB-dependent receptor
Rmet_1600		467±300	2,162	MET-AA-Urea	Q1LMZ3 Twin-arginine translocation pathway signal
Rmet_0273		217±19; (0.2; 1.3)	1,000	NA	Q1LRR7 Pirin-like protein

**AM0 down**		**AE104_0**	**AE104_M**		
Rmet_1870	*purL*	2,521±1,739; (3.4; 0.8)	215	MET-NUC-Pur	Q1LM77 PRGGA synthase
Rmet_0233		797±464; (0.6; 0.6)	73	NA	Q1LRV7 Thioredoxin-like protein
Rmet_0689		150±119; (1.0; 0.0)	17	NA	Q1LQK1 Putative uncharacterized protein
Rmet_0092		446±268; (6.0; 1.4)	57	NA	Q1LS98 Histidine kinase
Rmet_0896		427±250; (2.3; 0.6)	55	NA	Q1LPZ4 Transcriptional regulator of Mo metabolism
Rmet_5252	*fliC2*	1,0216±2,781; (4.0; 2.4)	1,344±658; (0.1; 2.6)	EIP-SIG-2Comp	Q1LCL5 Flagellin-like protein
Rmet_3520	*livG5*	841	112±70; (0.1; 10.4)	EIP-TRA-ABC	Q1LHI4 ABC transporter-related protein
Rmet_3689	*cheA*	498±355; (4.3; 0.9)	67	EIP-SIG-2Comp	Q1LH15 CheA histidine kinases
Rmet_4275	*bug*	311)	45±46; (0.1; 5.7)	NA	Q1LFD5 Uncharacterized protein UPF0065
Rmet_2257	*bcsG*	120±69; (1.0; 0.1)	20	NA	Q1LL40 Putative membrane protein
**AE0 up**		**AE104_0**	**AE104_E**		
Rmet_1117	*cysK*	107	3,646±2,887; (34.1; 1.2)	MET-EN-Sulf	Q44004 Cysteine synthase
Rmet_1116	*ocd*	205±117; (1.2; 0.2)	1,932±1,288; (9.4; 1.2)	MET-AA-ArgPro	Q1LPC4 Ornithine cyclodeaminase
Rmet_0123		90±81; (0.6; 0.6)	622±236; (6.9; 1.7)	EIP-TRA-Pores	Q1LS67 TonB-dependent receptor
Rmet_5638		430±154; (1.2; 0.2)	2,566±1,249; 6.0; 1.5)	NA	Q1LBH9 Putative uncharacterized protein
Rmet_3172	*nirS*	25±20; (0.3; 2.6)	112	MET-EN-Nitrog	Q1LII2 Cytochrome d1, heme region
Rmet_2161		63±37; (0.5; 0.5)	263	NA	Q1LLD6 Putative uncharacterized protein
Rmet_2618		159	591±362; (3.7; 1.2)	NA	Q1LK31 Rieske (2Fe-2S) region
Rmet_2277	*tonB*	189	664±399; (3.5; 1.2)	NA	Q1LL20 TonB-like protein
Rmet_5321	*zniC*	44±8; (1.1; 0.2)	147±75; (3.3; 1.2)	NA	Q1LCE6 =uter membrane lipoprotein, NodT
Rmet_4378	*acpP*	36	112±75; (3.1; 1.0)	MET-LIP-FASyn	Q1LF33 Putative acyl carrier protein
**AE0 down**		**AE104_0**	**AE104_E**		
Rmet_6408		1,387±1251; (0.9; 0.1)	124	NA	NA
Rmet_1812		261±180; (1.3; 0.2)	31±18; (0.1; 1.2)	NA	Q1LMD5 Rieske (2Fe-2S) region
Rmet_0939	*nuoM*	573	77±47; (0.1; 10.6)	MET-EN-OxPhos	Q1LPV1 NADH-quinone oxidoreductase, chain M
Rmet_4590	*arnC*	662±447; (1.8; 0.7)	95±55; (0.1; 1.1)	NA	Q1LEH4 Glycosyl transferase, family 2
Rmet_0092		446±268; (6.0; 1.4)	71	NA	Q1LS98 Histidine kinase
Rmet_5251		80±55; (79.5; 1.4)	16	NA	Q1LCL6 Host factor-I protein
Rmet_3688	*motB*	66±38; (66.0; 1.7)	14±10; (0.2; 1.1)	EIP-TRA-Pores	Q1LH16 OmpA/MotB
Rmet_2857	*tauB*	208	46±27; (0.2; 6.1)	EIP-TRA-ABC	Q1LJE6 ABC transporter-related protein
Rmet_4201		967	219±133; (0.2; 5.6)	NA	Q1LFK8 Aminotransferase
Rmet_3737	*flgE*	59±3; (58.8; 13.5)	16	DIV-MOT-Flagel	Q1LGW7 Putative uncharacterized protein

The proteins with the highest degrees of up- or down-regulation in the comparisons CM0 (CH34 metal-shocked to control), CE0 (metal starvation to control); 0AC (AE104 to CH34 under non-challenging conditions), AM0 (AE104 metal-shocked to control) and AE0 (metal-starvation to control) were listed. The rows give the the respective copy numbers per cell for the compared conditions, the Q ratios and the distance value D. The untreated AE104 control cells were compared to untreated CH34 cells. Copy numbers without deviation indicates a single appearance in the triplicate measurement. In these cases, the Q and D values were not indicated. KO gives the KEGG orthology levels. First levels are division (DIV), Envelope and information processing (EIP), Genetic information processing (GIP) and Metabolism (MET), the second abbreviation the second level such as transport (TRA), carbohydrate metabolism (CAH), motility (MOT), and the third the third level such a P-type ATPase (P_ATPase).

Supplementary Table S6. Prediction of the copy number of proteins from the abundance of the sense mRNA and the antisense asRNA.

The first data set are the NPKM values for the mRNA and asRNA. The next table provides the lg10(a) and lg10(b) values for the linear curve fitting shown in Suppl. Fig. S3 and subsequently the a- and b-values. These were grouped into the AST classes as indicated by the color doe. The copy number of a protein is the product of a * b^lg10(mRNA). The lg(a) aand lg/b) values for AE104_E and AST4 (outlyer in Fig. 1) were the mean values from the AE104_E data points for AST3 and AST5 and are in italics (simulated value).

**RNA abundance (NPKM)**		CH34_0	CH34_0	CH34_M	CH34_M	CH34_E	CH34_E	AE104_0	AE104_0	AE104_M	AE104_M	AE104_E	AE104_E
**Uptake systems systems**		**mRNA**	**asRNA**	**mRNA2**	**asRNA2**	**mRNA3**	**asRNA3**	**mRNA4**	**asRNA4**	**mRNA5**	**asRNA5**	**mRNA6**	**asRNA6**
Rmet_3052	*corA1*	55.00	124.67	30.33	2.00	57.67	129.33	57.00	103.67	53.67	100.00	59.67	118.67
Rmet_0036	*corA2*	66.33	373.00	53.67	137.33	62.33	349.33	61.00	1.33	51.67	201.00	60.33	304.33
Rmet_3287	*corA3*	114.00	853.33	104.67	409.67	110.00	896.33	137.33	943.67	124.00	567.67	108.54	264.67
Rmet_2621	*zupT*	151.00	61.00	108.00	52.33	246.67	71.67	154.33	72.33	119.67	54.67	193.00	61.67
Rmet_1533	*hoxN*	183.33	5.67	248.67	3.33	179.33	5.33	150.00	4.67	142.00	5.00	126.67	5.33
Rmet_1973	*pitA*	280.33	99.33	95.33	165.00	295.67	100.00	360.00	81.33	232.67	144.00	322.33	125.67
Rmet_5396	*mgtA*	12.33	1.67	17.00	1.00	13.00	0.33	11.67	0.00	11.67	2.67	12.67	0.67
Rmet_2211	*mgtB*	19.67	2.00	20.67	29.33	22.00	4.33	19.33	3.33	20.33	33.67	21.33	5.33
Rmet_0549	*zntB*	12.67	2.00	7.33	3.33	12.33	1.67	12.67	2.00	7.67	1.00	11.67	2.33
Rmet_5890	*feoB*	110.33	14.33	157.33	11.67	134.00	13.00	114.33	13.33	149.33	9.00	122.00	0.00
Rmet_5891	*feoA*	131.67	14.33	174.67	11.67	162.67	13.00	121.67	13.33	185.33	9.00	136.67	15.33
**Efflux systems**													
Rmet_2299	*cdfX*	36.67	0.00	242.00	0.00	12.47	0.00	31.53	0.00	99.22	0.00	10.72	0.00
Rmet_4594	*zntA*	41.67	77.33	3049.00	428.33	16.78	90.67	51.33	42.67	774.33	147.67	23.27	71.60
Rmet_2303	*cadA*	10.33	50.00	641.67	94.33	5.00	50.33	12.33	40.33	51.00	71.00	6.33	43.67
Rmet_5947	*pbrA*	8.00	1.67	240.00	5.00	6.00	1.33						
Rmet_5970	*czcP*	7.67	0.67	74.67	560.00	8.67							
Rmet_3524	*cupA*	15.00	47.33	1897.33	473.33	16.00	38.67	15.00	36.33	802.00	1144.33	15.00	31.67
Rmet_6119	*copF*	41.33	10.00	552.33	89.67	41.33	20.00						
Rmet_2379	*ctpA1*	43.00		42.14		45.15		38.70		34.55		37.47	
Rmet_2046	*rdxI*	250.33	5.67	103.33	7.33	250.67	6.00	271.33	3.33	113.67	6.33	271.67	4.33
Rmet_5979	*czcD*	13.67	1.33	381.33	158.33	6.33	1.67						
Rmet_0198	*dmeF*	44.33	173.67	58.67	133.33	39.67	176.67	44.33	161.67	61.67	135.67	36.34	164.33
Rmet_3406	*fieF*	108.00	30.67	262.00	164.67	132.69	30.34	106.33	24.00	139.00	73.33	125.17	29.26
Rmet_6211	*cnrT*	47.33	21.33	230.00	20.00	49.33	16.67						
Rmet_0391	*atmA*	58.00	42.33	213.33	20.00	63.33	38.00	58.67	25.00	84.67	33.67	55.67	42.00

Functions

**AST class**	**lg(a)-values**	**lg(a)**	**lg(b)**	**lg(a)**	**lg(b)**	**lg(a)**	**lg(b)**	**lg(a)**	**lg(b)**	**lg(a)**	**lg(b)**	**lg(a)**	**lg(b)**	
AST0		1.2921	0.4936	1.5106	0.305	1.6501	0.223	1.9708	0.051973	2.0247	0.086196	1.8958	0.095919	
AST1		1.2347	0.5678	1.3729	0.4793	1.3726	0.4843	1.241	0.557	1.3926	0.4856	1.2048	0.5624	
AST2		1.0573	0.6966	1.2007	0.618	1.1545	0.6326	1.1638	0.6397	1.2714	0.5871	1.0682	0.6726	
AST3		0.8847	0.7733	1.1259	0.6745	0.9287	0.7586	0.8825	0.7618	1.2623	0.5978	1.0465	0.6913	
AST4		0.6916	0.8604	1.1681	0.6078	0.799	0.8156	0.7168	0.8197	0.9545	0.7393	***0.83565***	***0.7795***	sim val
AST5		0.9304	0.7469	1.2757	0.5709	1.0108	0.7179	0.965	0.7299	1.1732	0.6323	1.0124	0.703	
		**a**	**b**	**a**	**b**	**a**	**b**	**a**	**b**	**a**	**b**	**a**	**b**	
AST0		19.6	3.12	32.4	2.02	44.7	1.67	93.5	1.13	105.9	1.22	78.7	1.25	
AST1		17.2	3.70	23.6	3.02	23.6	3.05	17.4	3.61	24.7	3.06	16.0	3.65	
AST2		11.4	4.97	15.9	4.15	14.3	4.29	14.6	4.36	18.7	3.86	11.7	4.71	
AST3		7.7	5.93	13.4	4.73	8.5	5.74	7.6	5.78	18.3	3.96	11.1	4.91	
AST4		4.9	7.25	14.7	4.05	6.3	6.54	5.2	6.60	9.0	5.49	6.8	***6.02***	
AST5		8.5	5.58	18.9	3.72	10.3	5.22	9.2	5.37	14.9	4.29	10.3	5.05	

**Relevant factors for preduction**		a	b	a	b	a	b	a	b	a	b	a	b
**Uptake systems systems**													
Rmet_3052	*corA1*	8.5	5.58	23.6	3.02	10.3	5.22	9.2	5.37	14.9	4.29	10.3	5.05
Rmet_0036	*corA2*	8.5	5.58	18.9	3.72	10.3	5.22	17.4	3.61	14.9	4.29	10.3	5.05
Rmet_3287	*corA3*	8.5	5.58	18.9	3.72	10.3	5.22	9.2	5.37	14.9	4.29	10.3	5.05
Rmet_2621	*zupT*	4.9	7.25	14.7	4.05	6.3	6.54	5.2	6.60	9.0	5.49	6.8	***6.02***
Rmet_1533	*hoxN*	11.4	4.97	15.9	4.15	14.3	4.29	14.6	4.36	18.7	3.86	11.7	4.71
Rmet_1973	*pitA*	4.9	7.25	18.9	3.72	10.3	5.22	5.2	6.60	14.9	4.29	10.3	5.05
Rmet_5396	*mgtA*	17.2	3.70	23.6	3.02	23.6	3.05	93.5	1.13	24.7	3.06	16.0	3.65
Rmet_2211	*mgtB*	17.2	3.70	13.4	4.73	14.3	4.29	14.6	4.36	18.3	3.96	11.7	4.71
Rmet_0549	*zntB*	17.2	3.70	15.9	4.15	23.6	3.05	17.4	3.61	24.7	3.06	16.0	3.65
Rmet_5890	*feoB*	7.7	5.93	13.4	4.73	8.5	5.74	7.6	5.78	18.7	3.86	78.7	1.25
Rmet_5891	*feoA*	7.7	5.93	13.4	4.73	8.5	5.74	7.6	5.78	18.7	3.86	11.1	4.91
**Efflux systems**													
Rmet_2299	*cdfX*	19.6	3.12	32.4	2.02	44.7	1.67	93.5	1.13	105.9	1.22	78.7	1.25
Rmet_4594	*zntA*	4.9	7.25	18.9	3.72	6.3	6.54	5.2	6.60	14.9	4.29	6.8	***6.02***
Rmet_2303	*cadA*	4.9	7.25	14.7	4.05	6.3	6.54	5.2	6.60	9.0	5.49	6.8	***6.02***
Rmet_5947	*pbrA*	17.2	3.70	15.9	4.15	23.6	3.05						
Rmet_5970	*czcP*	17.2	3.70	18.9	3.72	44.7	1.67						
Rmet_3524	*cupA*	4.9	7.25	18.9	3.72	6.3	6.54	5.2	6.60	14.9	4.29	6.8	***6.02***
Rmet_6119	*copF*	7.7	5.93	14.7	4.05	8.5	5.74						
Rmet_2379	*ctpA1*	19.6	3.12	32.4	2.02	44.7	1.67	93.5	1.13	105.9	1.22	78.7	1.25
Rmet_2046	*rdxI*	11.4	4.97	15.9	4.15	14.3	4.29	14.6	4.36	18.7	3.86	11.7	4.71
Rmet_5979	*czcD*	17.2	3.70	18.9	3.72	23.6	3.05						
Rmet_0198	*dmeF*	8.5	5.58	18.9	3.72	10.3	5.22	9.2	5.37	14.9	4.29	10.3	5.05
Rmet_3406	*fieF*	4.9	7.25	18.9	3.72	6.3	6.54	7.6	5.78	9.0	5.49	11.1	4.91
Rmet_6211	*cnrT*	7.7	5.93	13.4	4.73	8.5	5.74						
Rmet_0391	*atmA*	7.7	5.93	13.4	4.73	6.3	6.54	7.6	5.78	9.0	5.49	6.8	***6.02***

log10(mRNA)

**Uptake systems systems**							
Rmet_3052	*corA1*	1.74	1.48	1.76	1.76	1.73	1.78
Rmet_0036	*corA2*	1.82	1.73	1.79	1.79	1.71	1.78
Rmet_3287	*corA3*	2.06	2.02	2.04	2.14	2.09	2.04
Rmet_2621	*zupT*	2.18	2.03	2.39	2.19	2.08	2.29
Rmet_1533	*hoxN*	2.26	2.40	2.25	2.18	2.15	2.10
Rmet_1973	*pitA*	2.45	1.98	2.47	2.56	2.37	2.51
Rmet_5396	*mgtA*	1.09	1.23	1.11	1.07	1.07	1.10
Rmet_2211	*mgtB*	1.29	1.32	1.34	1.29	1.31	1.33
Rmet_0549	*zntB*	1.10	0.87	1.09	1.10	0.88	1.07
Rmet_5890	*feoB*	2.04	2.20	2.13	2.06	2.17	2.09
Rmet_5891	*feoA*	2.12	2.24	2.21	2.09	2.27	2.14
**Efflux systems**							
Rmet_2299	*cdfX*	1.56	2.38	1.10	1.50	2.00	1.03
Rmet_4594	*zntA*	1.62	3.48	1.22	1.71	2.89	1.37
Rmet_2303	*cadA*	1.01	2.81	0.70	1.09	1.71	0.80
Rmet_5947	*pbrA*	0.90	2.38	0.78			
Rmet_5970	*czcP*	0.88	1.87	0.94			
Rmet_3524	*cupA*	1.18	3.28	1.20	1.18	2.90	1.18
Rmet_6119	*copF*	1.62	2.74	1.62			
Rmet_2379	*ctpA1*	1.63	1.62	1.65	1.59	1.54	1.57
Rmet_2046	*rdxI*	2.40	2.01	2.40	2.43	2.06	2.43
Rmet_5979	*czcD*	1.14	2.58	0.80			
Rmet_0198	*dmeF*	1.65	1.77	1.60	1.65	1.79	1.56
Rmet_3406	*fieF*	2.03	2.42	2.12	2.03	2.14	2.10
Rmet_6211	*cnrT*	1.68	2.36	1.69			
Rmet_0391	*atmA*	1.76	2.33	1.80	1.77	1.93	1.75

Estimated protein copy number

**Uptake systems systems**							
Rmet_3052	*corA1*	170	121	188	176	185	182
Rmet_0036	*corA2*	195	183	199	172	180	184
Rmet_3287	*corA3*	293	268	299	335	314	278
Rmet_2621	*zupT*	368	254	562	324	310	414
Rmet_1533	*hoxN*	430	480	380	360	343	304
Rmet_1973	*pitA*	627	254	609	649	467	597
Rmet_5396	*mgtA*	71	92	82	106	81	67
Rmet_2211	*mgtB*	93	103	101	97	111	92
Rmet_0549	*zntB*	73	54	80	72	66	64
Rmet_5890	*feoB*	291	405	349	282	353	125
Rmet_5891	*feoA*	334	435	404	296	401	333
**Efflux systems**							
Rmet_2299	*cdfX*	116	173	78	112	157	99
Rmet_4594	*zntA*	122	1840	63	131	1000	80
Rmet_2303	*cadA*	37	749	23	41	165	29
Rmet_5947	*pbrA*	56	470	56			
Rmet_5970	*czcP*	55	221	72			
Rmet_3524	*cupA*	51	1403	60	48	1022	57
Rmet_6119	*copF*	136	684	143			
Rmet_2379	*ctpA1*	125	101	104	113	144	111
Rmet_2046	*rdxI*	535	279	470	525	301	507
Rmet_5979	*czcD*	76	562	58			
Rmet_0198	*dmeF*	145	193	144	147	202	129
Rmet_3406	*fieF*	276	453	339	267	346	314
Rmet_6211	*cnrT*	151	523	163			
Rmet_0391	*atmA*	177	498	186	170	240	157

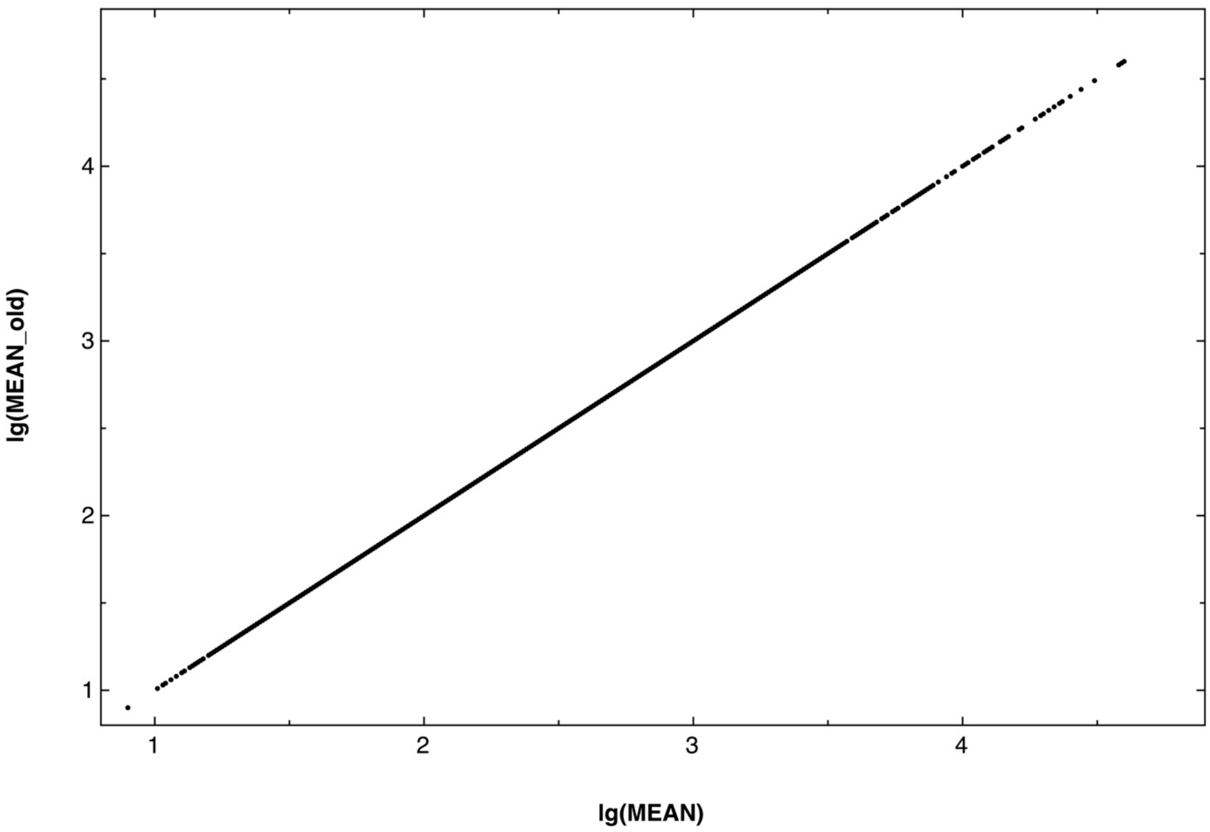

Supplementary Figure S1. Comparison of the copy numbers of the proteins in the proteome of *C. metallidurans* AE104 under non-challenging conditions. The copy numbers of the proteins determined here and published (Mean:old, [2]) were plotted against each other in a double-log10-plot. Regression coefficient 100%, function lg(Mean_old) = -3.995 ± 0.076 * 10^-14 + 1.0000 ± 3.16*10^-16 * lg(Mean). The data of the published proteome determination and the newly determined results were comparable.

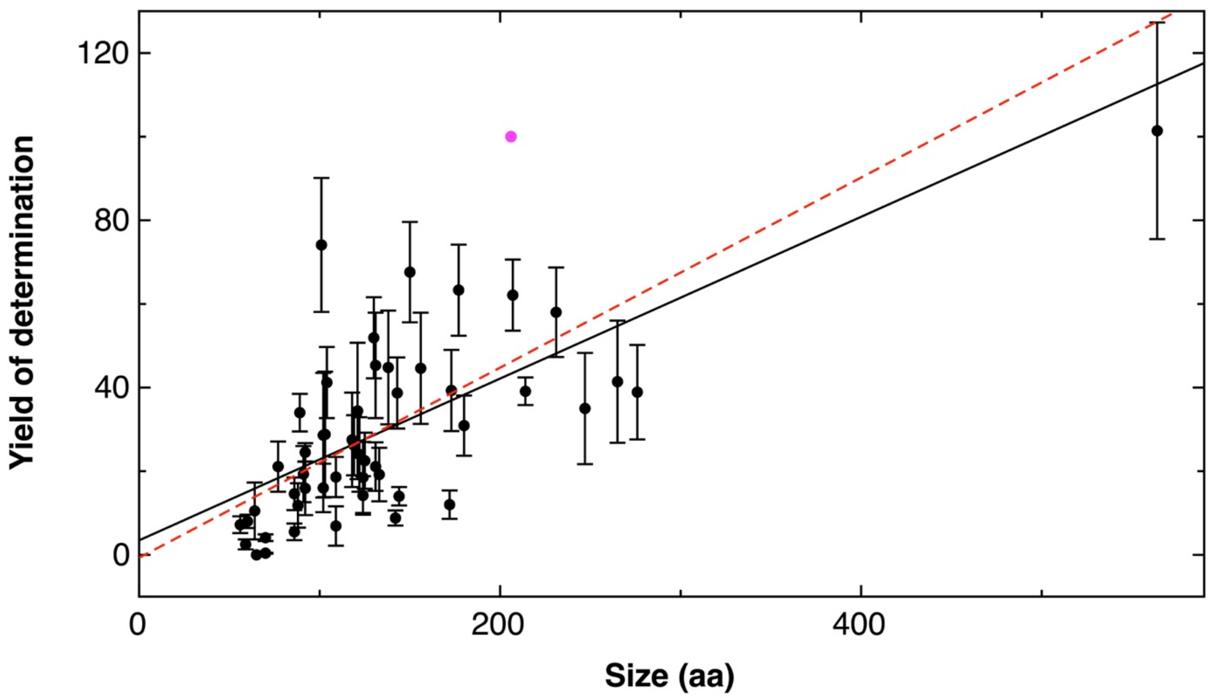

Supplementary Figure S2. Detection efficiency of ribosomal proteins in comparison to RplD. The mean values of the copy numbers of the ribosomal proteins were normalized to RplD (magenta, 100%) and plotted against the size. The determination yield increases with the size for proteins up to 200 aa with a regression coefficient of 45.8% and a slope of 0.193 ± 0.030 % per aa. Omitting RpsA (upper right corner) did not change the function (58.4%, slope 0.227 ± 0.046 %/aa, red dashed line).

Supplementary Figure S3. Dependence of the protein abundance from that of the transcript of its gene. The abundance of proteins (copy number per cell) was plotted as decadic logarithm against that of the abundance of its transcript (NPKM values as published [3]). Closed circles are CH34 control cells, open circles metal-, squares EDTA-treated cells of strain CH34. Diamonds are AE104 control cells, triangles metal- and inverted triangles EDTA-treated AE104 cells. The data points were grouped according to the abundance (NPKM values as published [3]) of the respective asRNA into six groups: no asRNA (AST0, blue), NPKM ≤ 3 (AST1, green), 3 < NPKM ≤ 10 (AST2, grey), 10 < NPKM ≤ 30 (AST3, black), 30 < NPKM ≤ 100 (AST4, red) and NPKM > 100 (AST5, magenta and smaller symbols). A linear curve fit was performed for the six groups to the equation "lg_{10}(protein) = lg_{10}(a) + lg_{10}(b) * lg_{10}(senseRNA)". The resulting parameters are given in the Suppl. Table S6 and the functions shown as lines in the respective color. Please note that in all panels and condition the slope of the function increased with the increasing abundance of the asRNA.

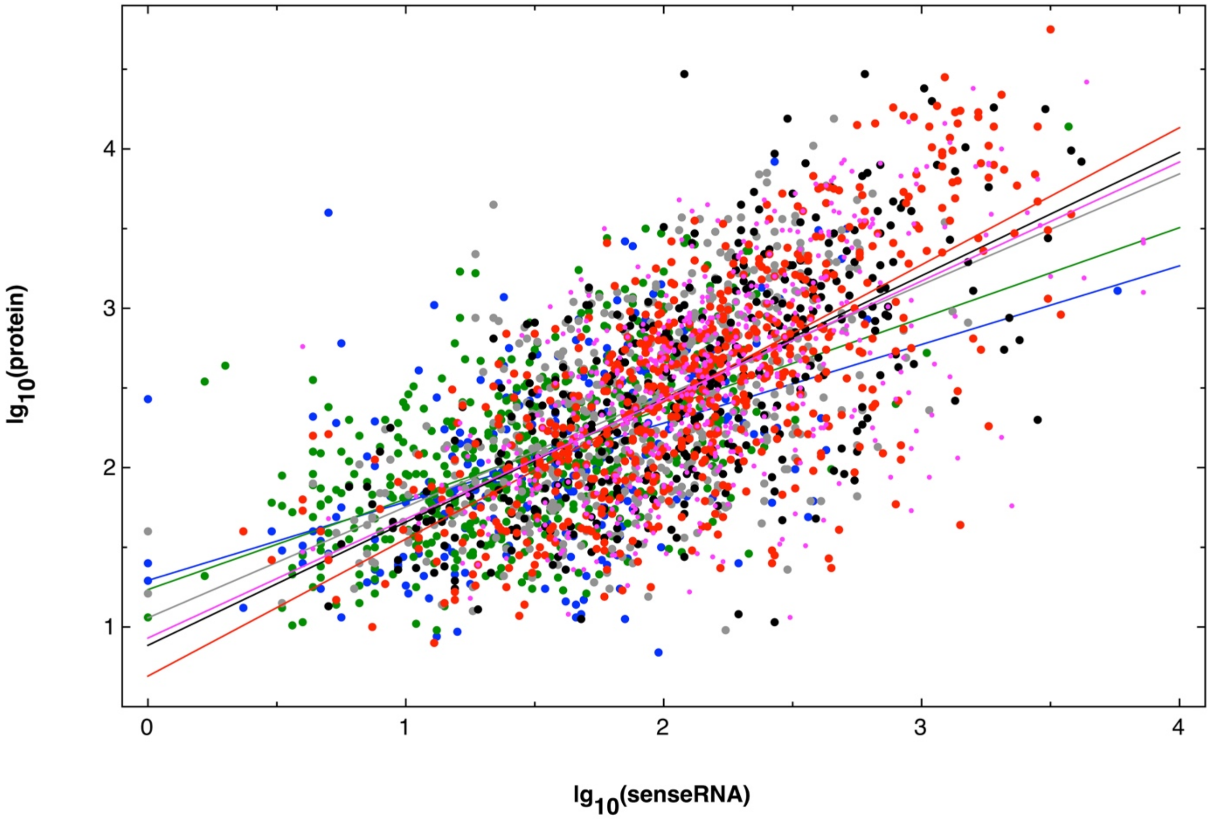

Supplementary Figure S3a. Unchallenged control cells of *C. metallidurans* strain CH34.

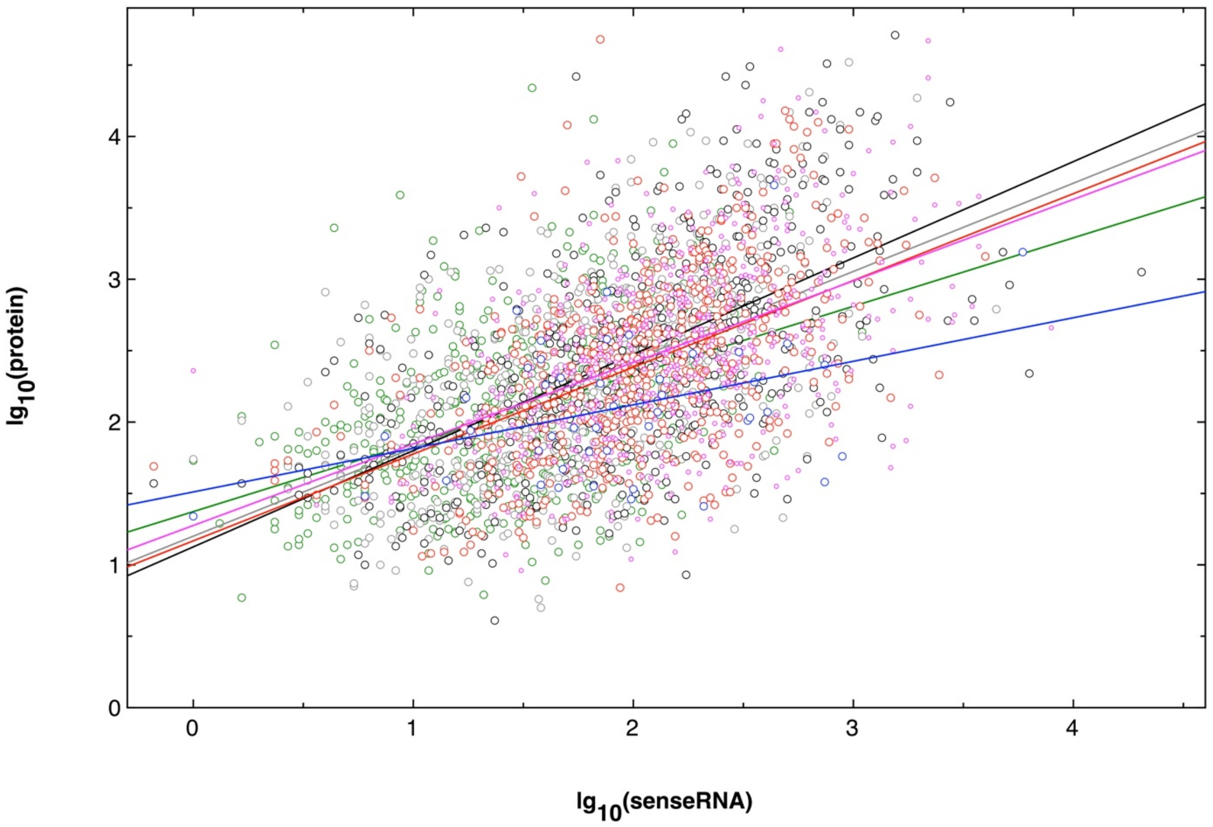

Supplementary Figure S3b. Metal-stressed cells of *C. metallidurans* strain CH34.

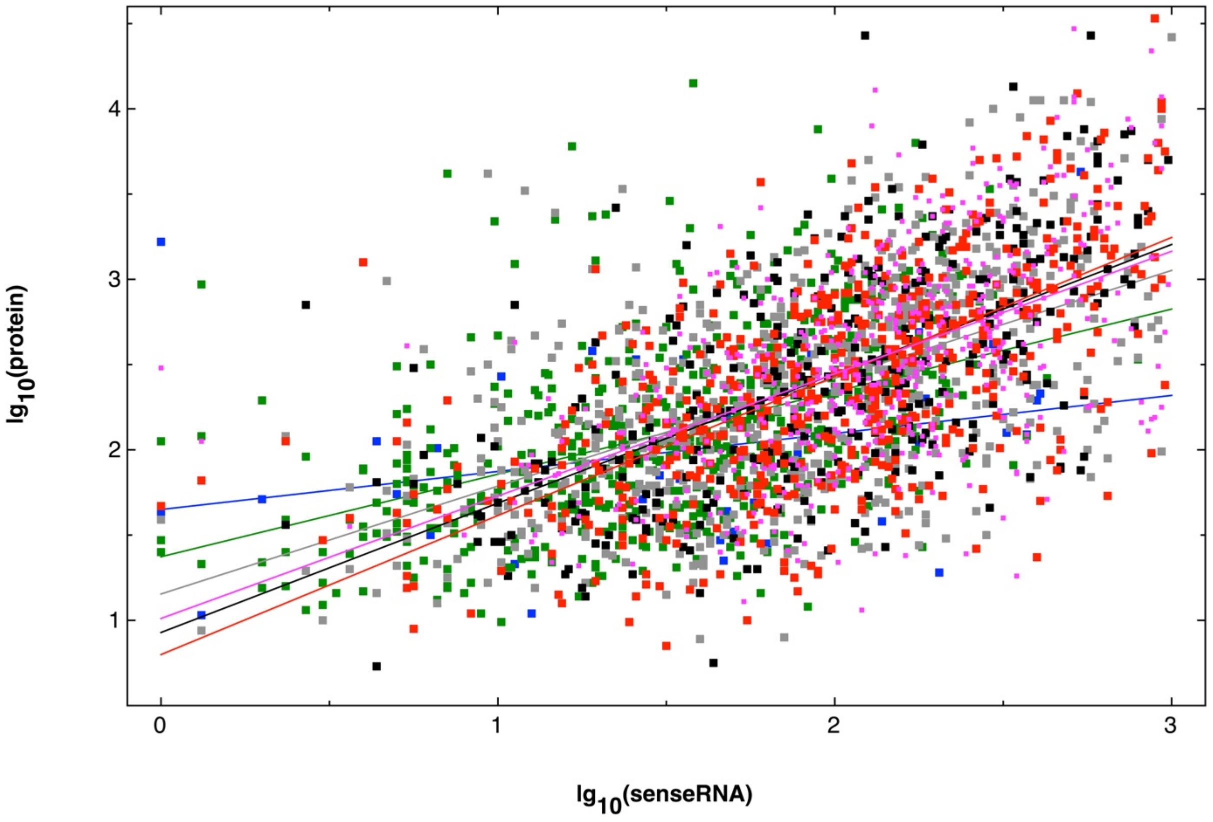

Supplementary Figure S3c. Metal-starved cells of *C. metallidurans* strain CH34.

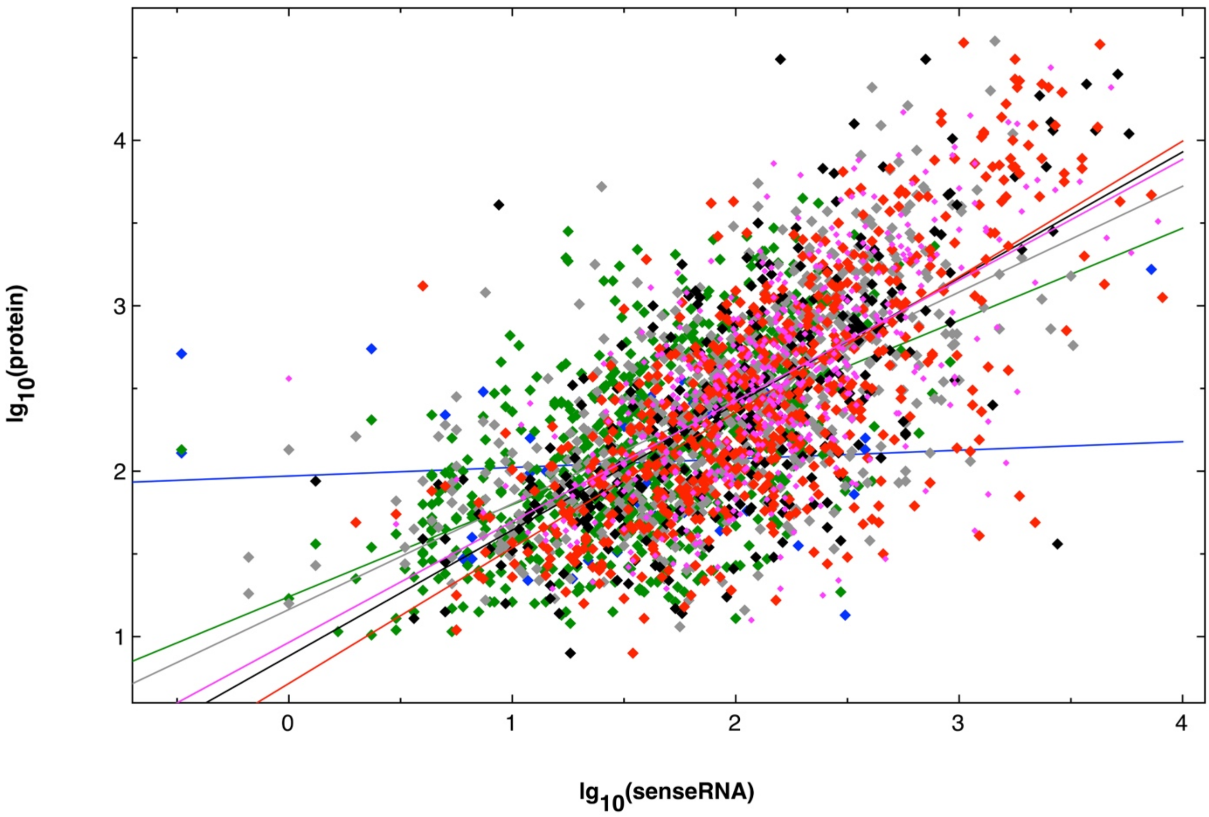

Supplementary Figure S3d. Unchallenged control cells of *C. metallidurans* strain AE104.

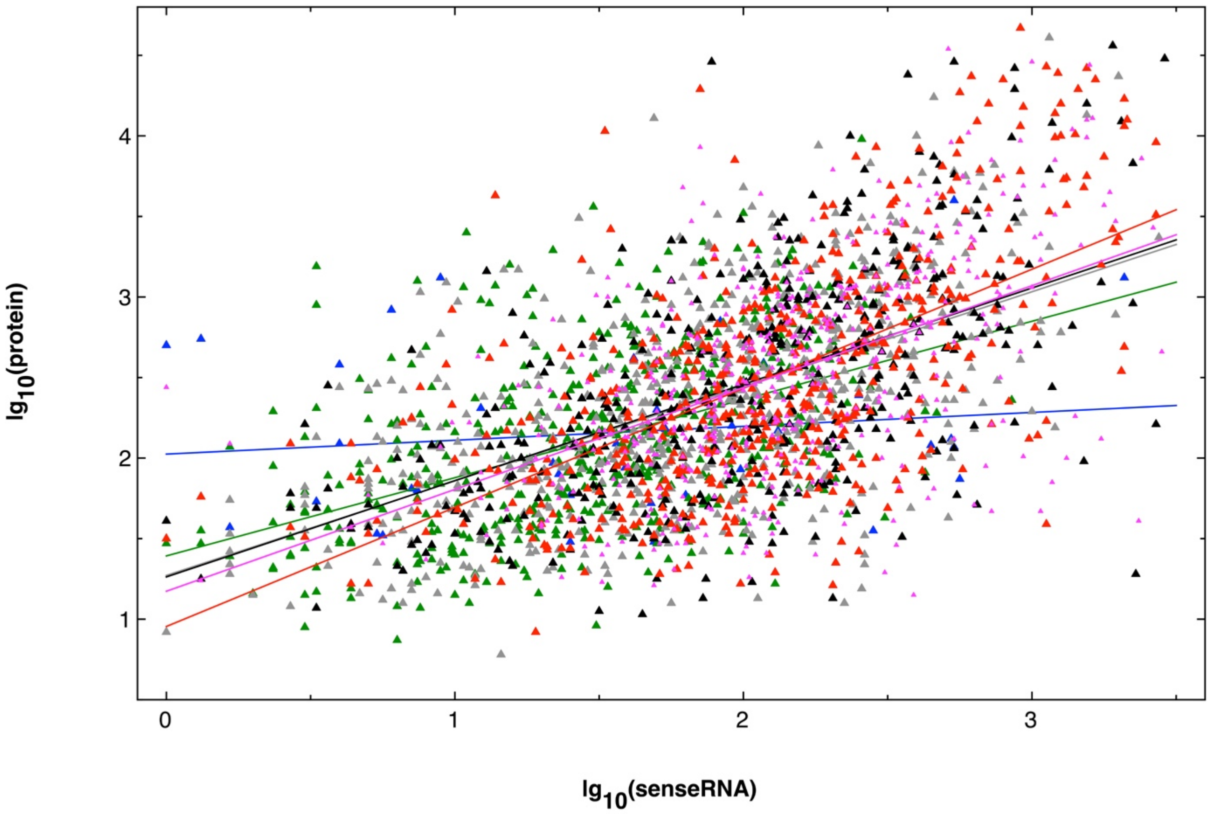

Supplementary Figure S3e. Metal-stressed cells of *C. metallidurans* strain AE104.

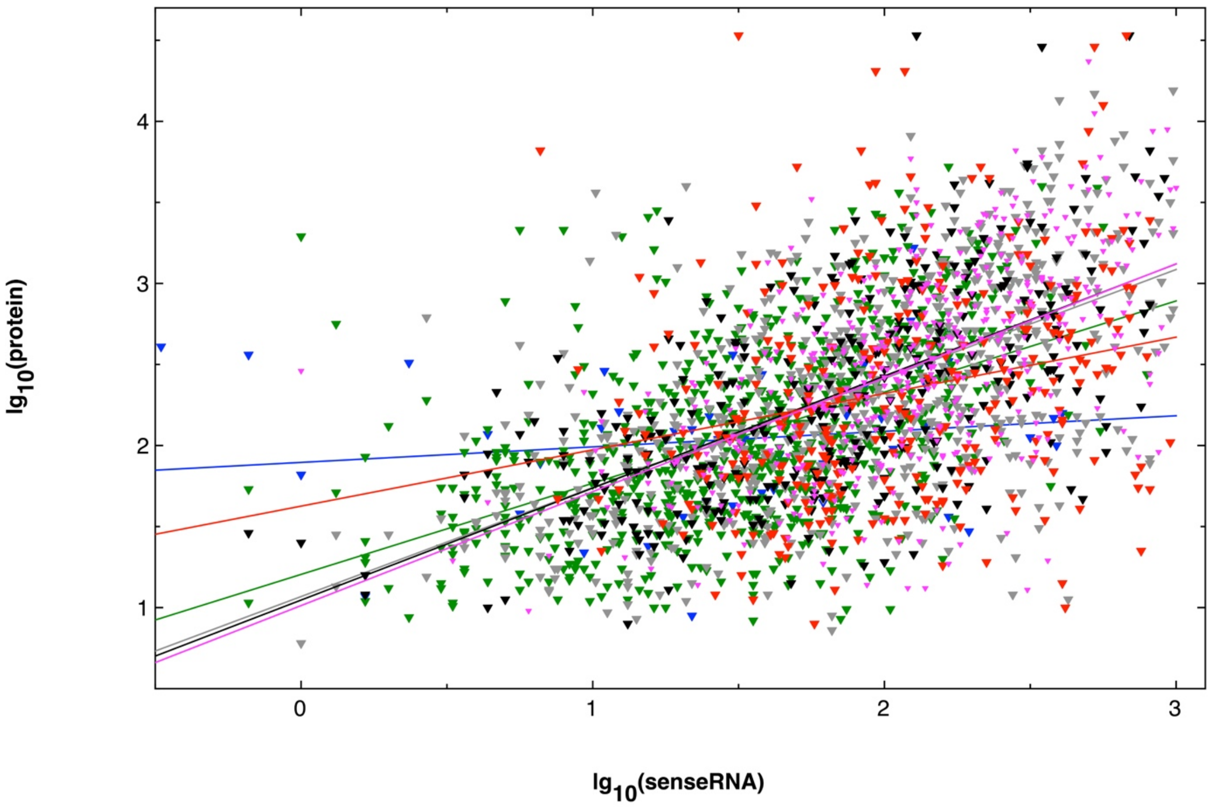

Supplementary Figure S3f. Metal-starved cells of *C. metallidurans* strain AE104.

Literature of the Supplement

1. Junge W, Sielaff H, Engelbrecht S. Torque generation and elastic power transmission in the rotary F(O)F(1)-ATPase. *Nature* 2009;459(7245):364-70. doi: 10.1038/nature08145
2. Herzberg M, Dobritzsch D, Helm S *et al.* The zinc repository of *Cupriavidus metallidurans*. *Metallomics* 2014;6:2157-65 doi: DOI: 10.1039/C4MT00171K
3. Große C, Grau J, Herzberg M *et al.* Antisense transcription is associated with expression of metal resistance determinants in *Cupriavidus metallidurans* CH34. in revision
